# Proceedings of the 7th Biannual International Symposium on Nasopharyngeal Carcinoma 2015: Yogyakarta, Indonesia. 4-6 June 2015

**DOI:** 10.1186/s12919-016-0001-5

**Published:** 2016-04-13

**Authors:** IB Tan, Ellen T. Chang, Chien-Jen Chen, Wan-Lun Hsu, Yin-Chu Chien, Allan Hildesheim, James D. McKay, Valerie Gaborieau, Mohamed Arifin Bin Kaderi, Dewajani Purnomosari, Catherine Voegele, Florence LeCalvez-Kelm, Graham Byrnes, Paul Brennan, Beena Devi, L. Li, Y. Zhang, Y. Fan, K. Sun, Z. Du, H. Sun, A. T. Chan, S. W. Tsao, Y. X. Zeng, Q. Tao, Pierre Busson, Claire Lhuillier, Olivier Morales, Dhafer Mrizak, Aurore Gelin, Nikiforos Kapetanakis, Nadira Delhem, Sheila Mansouri, Jennifer Cao, Anup Vaidya, Lori Frappier, Lo Kwok Wai, Sui-Hong Chen, Jin-lin Du, Ming-Fang Ji, Qi-Hong Huang, Qing Liu, Su-Mei Cao, Denise L. Doolan, Anna Coghill, Jason Mulvenna, Carla Proietti, Lea Lekieffre, Jeffrey Bethony, and Allan Hildesheim, Renske Fles, Sagung Rai Indrasari, Camelia Herdini, Santi Martini, Atoillah Isfandiari, Achmad Rhomdoni, Marlinda Adham, Ika Mayangsari, Erik van Werkhoven, Maarten Wildeman, Bambang Hariwiyanto, Bambang Hermani, Widodo Ario Kentjono, Sofia Mubarika Haryana, Marjanka Schmidt, IB Tan, Brian O’Sullivan, Enis Ozyar, Anne W. M. Lee, Mu-Sheng Zeng, Xiaojiang Gao, Minzhong Tang, Pat Martin, Yi Zeng, Mary Carrington, Anna E. Coghill, Wei Bu, Hanh Nguyen, Wan-Lun Hsu, Kelly J. Yu, Pei-Jen Lou, Cheng-Ping Wang, Chien-Jen Chen, Allan Hildesheim, Jeffrey I. Cohen, Ann D. King, Yin-Chu Chien, Wan-Lun Hsu, Kelly J. Yu, Tseng-Cheng Chen, Ching-Yuan Lin, Yung-An Tsou, Yi-Shing Leu, Li-Jen Laio, Yen-Liang Chang, Cheng-Ping Wang, Chun-Hun Hua, Ming-Shiang Wu, Chu-Hsing Kate Hsiao, Jehn-Chuan Lee, Ming-Hsui Tsai, Skye Hung-Chun Cheng, Pei-Jen Lou, Allan Hildesheim, Chien-Jen Chen, Wan-Lun Hsu, Kelly J. Yu, Yin-Chu Chien, Tseng-Cheng Chen, Ching-Yuan Lin, Yung-An Tsou, Yi-Shing Leu, Li-Jen Liao, Yen-Liang Chang, Tsung-Lin Yang, Chun-Hun Hua, Ming-Shiang Wu, Chu-Hsing Kate Hsiao, Jehn-Chuan Lee, Ming-Hsui Tsai, Skye Hung-Chun Cheng, Jenq-Yuh Ko, Allan Hildesheim, Chien-Jen Chen, Josephine Mun Yee Ko, Wei Dai, Dora Kwong, Wai Tong Ng, Anne Lee, Roger Kai Cheong Ngan, Chun Chung Yau, Stewart Tung, Maria Li Lung, Mingfang Ji, Wei Sheng, Mun Hon Ng, Weimin Cheng, Xia Yu, Biaohua Wu, Kuangrong Wei, Jun Zhan, Yi Xin Zeng, Su Mei Cao, Ningshao Xia, Yong Yuan, Qian Cui, Miao Xu, Jin-Xin Bei, Yi-Xin Zeng, B Şahin, A Dizman, M Esassolak, A Saran İkizler, HC Yıldırım, M Çaloğlu, B Atalar, F Akman, C Demiroz, BM Atasoy, E Canyilmaz, S Igdem, G Ugurluer, T Kütük, M Akmansoy, E Ozyar, Kiattisa Sommat, Fu Qiang Wang, Li-Lian Kwok, Terence Tan, Kam Weng Fong, Yoke Lim Soong, Shie Lee Cheah, Joseph Wee, M Casanova, E Özyar, C Patte, D Orbach, A Ferrari, VF Cristine, H Errihani, J Pan, L Zhang, S Liji, K Grzegorzewski, L Gore, A Varan, Susanna Hilda Hutajulu, Guntara Khuzairi, Camelia Herdini, Henry Kusumo, Mardiah Suci Hardianti, Kartika Widayati Taroeno-Hariadi, Ibnu Purwanto, Johan Kurnianda, Troy E. Messick, Kimberly Malecka, Lois Tolvinski, Samantha Soldan, Julianna Deakyne, Hui Song, Antonio van den Heuvel, Baiwei Gu, Joel Cassel, Mark McDonnell, Garry R. Smith, Venkata Velvadapu, Haiyan Bian, Yan Zhang, Marianne Carlsen, Shuai Chen, Alastair Donald, Christian Lemmen, Allen B. Reitz, Paul M. Lieberman, King Chi Chan, Lai Sheung Chan, Kwok Wai Lo, Timothy Tak Chun Yip, Roger Kai Cheong Ngan, Michael Kahn, Maria Li Lung, Nai Ki Mak, Fei-Fei Liu, Wafa Khaali, Juliette Thariat, Laurence Fantin, Flavia Spirito, Meriem Khyatti, El Khalil Ben Driss, Sylvain Olivero, Janet Maryanski, Alain Doglio, Mengxue Xia, Yunfei Xia, Hui Chang, Rachel Shaw, Pudji Rahaju, Mardiah Suci Hardianti, Sindhu Wisesa, Kartika Widayati Taroeno-Harijadi, Ibnu Purwanto, Bambang Hariwiyanto, Wigati Dhamiyati, Johan Kurnianda, Sang-Nee Tan, Sai-Peng Sim, Muhtarum Yusuf, Ahmad C. Romdhoni, Widodo Ario K, Fedik Abdul Rantam, Sugiyanto, Lina Aryati, Fajar Adi-Kusumo, Mardiah Suci Hardianti, SY Bintoro, R. Oktriani, C. Herawati, A. Surono, Sofia M. Haryana, L. Zhong, L. Li, B. B. Ma, A. T. Chan, Q. Tao, M. Kalra, M. Ngo, S. Perna, A. Leen, N. Lapteva, C. M. Rooney, S. Gottschalk, Elida Mustikaningtyas, Sri Herawati, Achmad C. Romdhoni, Mingfang Ji, Yarui Xu, Weimin Cheng, Shengxiang Ge, Fugui Li, M. H. Ng, Louise SY Tan, Benjamin Wong, C. M. Lim, Achmad C. Romdhoni, Fedik A. Rantam, Widodo Ario Kentjono, Deasy Z. Madani, Nur Akbar, Agung Dinasti Permana, Camelia Herdini, Sagung Rai Indrasari, Jajah Fachiroh, Dwi Hartati, T. Baning Rahayudjati, Iswandi Darwis, Susanna Hilda Hutajulu, Bambang Hariwiyanto, Wigati Dhamiyati, Ibnu Purwanto, Kartika Widayati Taroeno-Hariadi, Johan Kurnianda, Sindhu Wisesa, Mardiah Suci Hardianti, Susanna Hilda Hutajulu, Kartika Widayati Taroeno-Harijadi, Ibnu Purwanto, Camelia Herdini, Wigati Dhamiyati, Johan Kurnianda, Khoirul Anwar, Susanna Hilda Hutajulu, Sagung Rai Indrasari, Sri Retna Dwidanarti, Ibnu Purwanto, Kartika Widayati Taroeno-Hariadi, Johan Kurnianda, Dominicus Wendhy Pramana, Susanna Hilda Hutajulu, Bambang Hariwiyanto, Wigati Dhamiyati, Ibnu Purwanto, Kartika Widayati Taroeno-Hariadi, Johan Kurnianda, Diah Ari Safitri, Susanna Hilda Hutajulu, Camelia Herdini, Sri Retna Dwi Danarti, Ibnu Purwanto, Kartika Widayati Taroeno-Hariadi, Johan Kurnianda, Suryo A Taroeno, Sindhu Wisesa, Kartika Widayati Taroeno-Hariadi, Ibnu Purwanto, Bambang Hariwiyanto, Wigati Dhamiyati, Johan Kurnianda, I. Wijaya, A. Oehadian, D. Prasetya, Wan-Lun Hsu, Yin-Chu Chien, Kelly J Yu, Cheng-Ping Wang, Ching-Yuan Lin, Yung-An Tsou, Yi-Shing Leu, Li-Jen Liao, Yen-Liang Chang, Jenq-Yuh Ko, Chun-Hun Hua, Ming-Shiang Wu, Chu-Hsing Kate Hsiao, Jehn-Chuan Lee, Ming-Hsui Tsai, Skye Hung-Chun Cheng, Pei-Jen Lou, Allan Hildesheim, Chien-Jen Chen, Sukri Rahman, Bestari J. Budiman, Novialdi, Rahmadona, Dewi Yuri Lestari, C. Yin, A. Foussadier, E. Blein, C. Chen, N. Bournet Ammour, M. Khiatti, S. Cao, Dewi Syafriyetti Soeis Marzaini, Dwi Hartati, Baning Rahayujati, Camelia Herdini, Jajah Fachiroh, L. Gunawan, S. Mubarika Haryana, A. Surono, C. Herawati, Michael Hartono, Jajah Fachiroh, Umi Intansari, Dewi Kartikawati Paramita, Akmal Akbar, Jajah Fachiroh, Dewi Kartikawati Paramita, Benny Hermawan, T. Baning Rahayudjati, Dewi K. Paramita, Jajah Fachiroh, Gabriella Argy, Jajah Fachiroh, Dewi Kartikawati Paramita, Susanna Hilda Hutajulu, Theodora Caroline Sihotang, Jajah Fachiroh, Umi Intansari, Dewi Kartikawati Paramita, Daniel Joko Wahyono, Purnomo Soeharso, Dwi Anita Suryandari, Lisnawati, Zanil Musa, Bambang Hermani, Maelinda Daker, Yeo Jiun Tzen, Norhasimah Bakar, Asma’ Saiyidatina Aishah Abdul Rahman, Munirah Ahmad, Yeo Tiong Chia, Alan Khoo Soo Beng, Widyandani Sasikirana, Tirta Wardana, Muhammad Radifar, Cita Herawati, Agus Surono, Sofia Mubarika Haryana

**Affiliations:** The Netherlands Cancer Institute, Amsterdam, The Netherlands; Faculty of Medicine, Universitas Gadjah Mada/Dr Sardjito General Hospital, Yogyakarta, Indonesia; Health Sciences Practice, Exponent, Inc., ᅟ, ᅟ; Division of Epidemiology, Department of Health Research and Policy, Stanford University School of Medicine, ᅟ, CA USA; Genomics Research Center, Academia Sinica, Taipei, Taiwan; Graduate Institute of Epidemiology and Preventive Medicine College of Public Health, National Taiwan University, Taipei, Taiwan; Infections & Immunoepidemiology Branch, DCEG, NCI, ᅟ, USA; International Agency for Research on Cancer, Lyon, France; International Islamic University Malaysia, Kuantan, Pahang Malaysia; Faculty of Medicine, Universitas Gadjah Mada, Yogyakarta, Indonesia; Umum Hospital Sarawak, Sarawak, Malaysia; Cancer Epigenetics Laboratory, Department of Clinical Oncology, State Key Laboratory of Oncology in South China, Sir YK Pao Center for Cancer, Li Ka Shing Institute of Health Sciences, The Chinese University of Hong Kong, ᅟ, ᅟ; Department of Anatomy, The University of Hong Kong, ᅟ, Hong Kong; Université Paris-sud, CNRS UMR8126 and Gustave Roussy, Villejuif, France; Université de Lille, CNRS UMR 8161 and Institut Pasteur de Lille, Lille, France; Department of Molecular Genetics, University of Toronto, Toronto, Ontario Canada; The Chinese University of Hong Kong, ᅟ, Hong Kong; Department of Cancer Prevention Research, Sun Yat-sen University Cancer Center, State Key Laboratory of Oncology in Southern China, Guangzhou, 510060 Guangdong P.R. China; Infectious Diseases Program, QIMR Berghofer Institute of Medical Research, Brisbane, QLD Australia; Division of Cancer Epidemiology and Genetics, National Cancer Institute, Bethesda, MD USA; Department of Microbiology and Tropical Medicine, George Washington University, Washington, DC USA; Department of Head and Neck Surgery, Netherlands Cancer Institute, Amsterdam, The Netherlands; Department of Otorhinolaryngology Head and Neck Surgery, Faculty of Medicine, Universitas Gadjah Mada/Dr Sardjito General Hospital, Yogyakarta, Indonesia; Department of Public Health, Faculty of Medicine, Universitas Airlangga, Surabaya, Indonesia; Department of Otorhinolaryngology Head and Neck Surgery, Faculty of Medicine, Universitas Airlangga, Surabaya, Indonesia; Department of Otorhinolaryngology Head and Neck Surgery, Faculty of Medicine, Universitas Indonesia, Jakarta, Indonesia; Department of Biometrics, Netherlands Cancer Institute, Amsterdam, The Netherlands; Department of Head and Neck Surgery, Amsterdam Medical Center, Amsterdam, The Netherlands; Department of Histology and Cell Biology, Faculty of Medicine, Universitas Gadjah Mada, Yogyakarta, Indonesia; Division of Psychosocial Research and Epidemiology, Division of Molecular Pathology, Netherlands Cancer Institute, Amsterdam, The Netherlands; University of Toronto, Toronto, Canada; Department of Radiation Oncology, Acibadem University School of Medicine, Maslak, Istanbul Turkey; University of Hong Kong, Shenzhen Hospital, ᅟ, China; State Key Laboratory of Oncology in South China; Collaborative Innovation Center for Cancer Medicine, Sun Yat-sen University Cancer Center, Guangzhou, China; Cancer & Inflammation Program, National Cancer Institute, Frederick, Maryland USA; College of Life Sciences, Beijing University of Technology, Beijing, China; Wuzhou Red Cross Hospital, Guangxi, China; Infections and Immunoepidemiology Branch, Division of Cancer Epidemiology and Genetics, National Cancer Institute, Bethesda, Maryland USA; Laboratory of Infectious Diseases, National Institute of Allergy and Infectious Diseases, Bethesda, Maryland USA; Graduate Institute of Epidemiology, College of Public Health, National Taiwan University, Taipei, Taiwan; Genomics Research Center, Academia Sinica, Taipei, Taiwan; Division of Cancer Prevention, National Cancer Institute, Bethesda, Maryland USA; Department of Otolaryngology, National Taiwan University Hospital and College of Medicine, Taipei, Taiwan; The Chinese University of Hongkong, ᅟ, China; Genomics Research Center, Academia Sinica, Taipei, Taiwan; Division of Cancer Prevention, National Cancer Institute, Bethesda, MD USA; Department of Otolaryngology, National Taiwan University Hospital, ᅟ, ᅟ; National Taiwan University College of Medicine, Taipei, Taiwan; Department of Head and Neck Surgery, Koo Foundation Sun Yat-Sen Cancer Center, Taipei, Taiwan; Department of Otorhinolaryngology-Head and Neck Surgery, China Medical University Hospital, Taichung, Taiwan; Department of Otolaryngology-Head & Neck Surgery, Mackay Memorial Hospital, Taipei, Taiwan; Department of Otolaryngology, Far Eastern Memorial Hospital, Taipei, Taiwan; Department of Otolaryngology Head and Neck Surgery, Cathay General Hospital, Taipei, Taiwan; School of Medicine, Fu Jen Catholic University, Taipei, Taiwan; Department of Internal Medicine, National Taiwan University Hospital, Taipei, Taiwan; Health Management Center, National Taiwan University Hospital, Taipei, Taiwan; Graduate Institute of Epidemiology and Preventive Medicine College of Public Health, National Taiwan University, Taipei, Taiwan; Department of Radiation Oncology, Koo Foundation Sun Yat-Sen Cancer Center, Taipei, Taiwan; Division of Cancer Epidemiology and Genetics, National Cancer Institute, Bethesda, MD USA; Genomics Research Center, Academia Sinica, Taipei, Taiwan; Division of Cancer Prevention, National Cancer Institute, Bethesda, MD USA; Department of Otolaryngology, National Taiwan University Hospital and National Taiwan University College of Medicine, Taipei, Taiwan; Department of Head and Neck Surgery, Koo Foundation Sun Yat-Sen Cancer Center, Taipei, ᅟ; Department of Otorhinolaryngology-Head and Neck Surgery China Medical University Hospital, Taichung, Taiwan; Department of Otolaryngology-Head & Neck Surgery, Mackay Memorial Hospital, Taipei, ᅟ; Department of Otolaryngology, Far Eastern Memorial Hospital, Taipei, Taiwan; Department of Otolaryngology Head and Neck Surgery, Cathay General Hospital, Taipei, Taiwan; School of Medicine, Fu Jen Catholic University, Taipei, Taiwan; Department of Internal Medicine, National Taiwan University Hospital, Taipei, Taiwan; Health Management Center, National Taiwan University Hospital, Taipei, Taiwan; Graduate Institute of Epidemiology and Preventive Medicine College of Public Health, National Taiwan University, Taipei, Taiwan; Department of Radiation Oncology, Koo Foundation Sun Yat-Sen Cancer Center, Taipei, ᅟ; Division of Cancer Epidemiology and Genetics, National Cancer Institute, ᅟ, MD USA; Department of Clinical Oncology, University of Hong Kong, Pokfulam, Hong Kong SAR, People’s Republic of China; Center for Cancer Research, University of Hong Kong, Pokfulam, Hong Kong SAR, People’s Republic of China; Center for Nasopharyngeal Carcinoma Research, University of Hong Kong, Pokfulam, Hong Kong SAR, People’s Republic of China; Department of Clinical Oncology, Pamela Youde Nethersole Eastern Hospital, Chai Wan, Hong Kong SAR, People’s Republic of China; Department of Clinical Oncology, Hong Kong University-Shenzhen Hospital, Shenzhen, People’s Republic of China; Department of Clinical Oncology, Queen Elizabeth Hospital, Kowloon, Hong Kong SAR, People’s Republic of China; Department of Oncology, Princess Margaret Hospital, Kwai Chung, Hong Kong SAR, People’s Republic of China; Department of Clinical Oncology, TuenMun Hospital, TuenMun, Hong Kong SAR, People’s Republic of China; Cancer Research Institute of Zhongshan, Zhongshan, Guangdong province 528403 China; National Institute of Diagnostics and Vaccine Development in Infectious Disease, State Key Laboratory of Molecular Vaccinology and Molecular Diagnostics, School of Public Health, Xiamen University, Xiamen, 361005 China; Sun Yat-sen University Cancer Center, 651 Dongfeng Road East, Guangzhou, 510060 China; Sun Yat-sen University Cancer Center; State Key Laboratory of Oncology in South China; Collaborative Innovation Center for Cancer Medicine, Guanzhou, 510060 China; Departments of Radiation Oncology of Acibadem University, ᅟ, Turkey; Ankara Oncology Hospital, ᅟ, Turkey; Ege University, ᅟ, Turkey; Acibadem Bursa Hospital, ᅟ, Turkey; Cerrahpaşa University, ᅟ, Turkey; Trakya University, ᅟ, Turkey; Eylül University, ᅟ, Turkey; Uludağ University, ᅟ, Turkey; Marmara University, ᅟ, Turkey; Karadeniz Technical University, ᅟ, Turkey; Bilim University, ᅟ, Turkey; Acibadem Adana Hospital, ᅟ, Turkey; Ankara University, ᅟ, Turkey; Gazi University, ᅟ, Turkey; National Cancer Centre Singapore, ᅟ, Singapore; Fondazione IRCCS Istituto Nazionale dei Tumori, Milan, Italy; Acibadem University, School of Medicine, Istanbul, Turkey; Institut Gustave Roussy, Villejuif Cedex, France; Institut Curie, Paris, France; Sanofi, Chilly-Mazarin, France; National Institute of Oncology, University Mohammed V Souissi, Rabat, Morocco; Cancer Hospital of Fujian Provi, Fujian, China; State Key Laboratory of Oncology in South China, Collaborative Innovation Center for Cancer Medicine, Sun Yat-Sun University Cancer Center, East Guangzhou, China; Sanofi, Bridgewater, NJ USA; Novartis, East Hanover, NJ USA; The Children’s Hospital, Colorado, USA; Hacettepe University, Institute of Oncology, Ankara, Turkey; Division of Hematology and Medical Oncology, Department of Internal Medicine, Faculty of Medicine, Universitas Gadjah Mada/Dr Sardjito General Hospital, Yogyakarta, Indonesia; Study Program of Medicine, Faculty of Medicine, Universitas Gadjah Mada, Yogyakarta, Indonesia; Department of Otorhinolaryngology Head and Neck Surgery, Faculty of Medicine, Universitas Gadjah Mada/Dr Sardjito General Hospital, Yogyakarta, Indonesia; Department of Radiology, Faculty of Medicine, Universitas Gadjah Mada/Dr Sardjito General Hospital, Yogyakarta, Indonesia; The Wistar Institute, 3601 Spruce Street, Philadelphia, PA 19104 USA; Vironika, LLC, 3805 Old Easton Road, Doylestown, PA 18902 USA; Fox Chase Chemical Diversity Center, 3805 Old Easton Road, Doylestown, PA 18902 USA; Bio Solve IT GmbH, An der Ziegelei 79, 53757 Sankt Augustin, Germany; Department of Biology, Hong Kong Baptist University, Hong Kong, P.R. China; Department of Anatomical and Cellular Pathology, State Key Laboratory in Oncology in South China, The Chinese University of Hong Kong, Hong Kong, P.R. China; Department of Clinical Oncology, Queen Elizabeth Hospital, Hong Kong, P.R. China; Center for Nasopharyngeal Carcinoma Research, University of Hong Kong, Hong Kong, P.R. China; Center for Molecular Pathways and Drug Discovery, University of Southern California, Los Angeles, CS USA; Department of Clinical Oncology, University of Hong Kong, Hong Kong, P.R. China; Department of Radiation Oncology, Princess Margaret Cancer Center, University of Toronto, ᅟ, Canada; Centre Hospitalier Universitaire de Nice, Unité de Thérapie Cellulaire et Génique, Nice, France; Université Nice-Sophia Antipolis, UFR Médecine, MICORALIS, Nice, France; Institut Pasteur du Maroc, Laboratoire Onco-virologie, Casablanca, Morocco; Université Abdelmalek Essaâdi, Faculté des sciences, Laboratoire Ecologie, Biodiversité et Environnement, Tétouan, Morocco; Département de Radiothérapie, Centre Antoine-Lacassagne, Nice, France; Aston University, Birmingham, UK; Cancer Hospital of Sun Yat-Sen University, Guangzhou, China; Faculty of Medicine Brawijaya University, Malang, Indonesia; Division of Hematology and Medical Oncology, Department of Internal Medicine, Faculty of Medicine Universitas Gadjah Mada, Yogyakarta, Indonesia; Department of Anatomy, Faculty of Medicine, Jenderal Soedirman University, Purwokerto, Indonesia; Department of Otorhinolaryngology Head and Neck Surgery, Faculty of Medicine, Universitas Gadjah Mada, Yogyakarta, Indonesia; Division of Radiotherapy, Department of Radiology, Faculty of Medicine, Universitas Gadjah Mada, Yogyakarta, Indonesia; Faculty of Medicine and Health Sciences, Universiti Malaysia Sarawak, Sarawak, Malaysia; Oncology Division Departement of Otolaryngology Head and Neck sugery Faculty of Medicine Airlangga University/Dr. Soetomo Hospital, Surabaya, Indonesia; Institute of Tropical Disease Center, Universitas Airlangga, Surabaya, Indonesia; Department of Mathematics, Universitas Gadjah Mada, Yogyakarta, Indonesia; Faculty of Medicine, Universitas Gadjah Mada, Yogyakarta, Indonesia; Department of Molecular Biology, Faculty of Medicine, Universitas Gadjah Mada, Yogyakarta, Indonesia; Depatment of Otorhinolaryngology Head and Neck Surgery, Dharmais Cancer Hospital, Jakarta, Indonesia; Department of Otorhinolaryngology Head and Neck Surgery, Faculty of Medicine, Universitas Gadjah Mada/Dr Sardjito Hospital, Yogyakarta, Indonesia; Cancer Epigenetics Laboratory, Department of Clinical Oncology, State Key Laboratory of Oncology in South China, Sir YK Pao Center for Cancer, Li Ka Shing Institute of Health Sciences, The Chinese University of Hong Kong, ᅟ, Hong Kong; Center for Cell and Gene Therapy, Texas Children’s Hospital, Houston Methodist Hospital, Baylor College of Medicine, Houston, Texas 77030 USA; Department of Otorhinolaryngology Head and Neck Surgery, Faculty of Medicine, Universitas Airlangga, Surabaya, Indonesia; Stem Cell Laboratory, Institute of Tropical Disease, Universitas Airlangga, Surabaya, Indonesia; Cancer Research Institute of Zhongshan City, Zhongshan People’s Hospital, Zhongshan City, Guangdong Province PRC; National University Health System, ᅟ, Singapore; Department of Otorhinolaryngology Head and Neck Surgery, Faculty of Medicine, Universitas Airlangga, Surabaya, Indonesia; Stem Cell Laboratory, Institute of Tropical Disease, Universitas Airlangga, Surabaya, Indonesia; Department of Virology, Faculty of Veterinary, Universitas Airlangga, Surabaya, Indonesia; Department of Otorhinolaringology-Head and Neck Surgery, Faculty of Medicine, Padjajaran University/Dr.Hasan Sadikin General Hospital, Bandung, Indonesia; Department of Otolaryngology Head and Neck Surgery, Faculty of Medicine, Universitas Gadjah Mada/Dr Sardjito General Hospital, Yogyakarta, Indonesia; Department of Histology and Cell Biology, Faculty of Medicine, Universitas Gadjah Mada, Yogyakarta, Indonesia; Molecular Biology Laboratory, Faculty of Medicine, Universitas Gadjah Mada, Yogyakarta, Indonesia; Department of Public Health, Faculty of Medicine, Universitas Gadjah Mada, Yogyakarta, Indonesia; Study Program of Specialty in Internal Medicine, Department of Internal Medicine, Faculty of Medicine, Universitas Gadjah Mada, Yogyakarta, Indonesia; Division of Hematology and Medical Oncology, Faculty of Medicine, Universitas Gadjah Mada/Dr Sardjito General Hospital, Yogyakarta, Indonesia; Department of Otorhinolaryngology Head and Neck Surgery, Faculty of Medicine, Universitas Gadjah Mada/Dr Sardjito General Hospital, Yogyakarta, Indonesia; Division of Radiotherapy, Department of Radiology, Faculty of Medicine, Universitas Gadjah Mada/Dr Sardjito General Hospital, Yogyakarta, Indonesia; Department of Anatomy, Faculty of Medicine, Jenderal Soedirman University, Purwokerto, Indonesia; Division of Hematology and Medical Oncology, Department of Internal Medicine, Faculty of Medicine, Universitas Gadjah Mada/Dr Sardjito General Hospital, Yogyakarta, Indonesia; Department of Otorhinolaryngology Head and Neck Surgery, Faculty of Medicine Universitas Gadjah Mada/Dr Sardjito General Hospital, Yogyakarta, Indonesia; Division of Radiotherapy, Department of Radiology, Faculty of Medicine Universitas Gadjah Mada/Dr Sardjito General Hospital, Yogyakarta, Indonesia; Study Program of Specialty in Internal Medicine, Department of Internal Medicine, Faculty of Medicine, Universitas Gadjah Mada, Yogyakarta, Indonesia; Division of Hematology and Medical Oncology, Department of Internal Medicine, Faculty of Medicine, Universitas Gadjah Mada/Dr Sardjito General Hospital, Yogyakarta, Indonesia; Department of Otorhinolaryngology Head and Neck Surgery, Faculty of Medicine, Universitas Gadjah Mada/Dr Sardjito General Hospital, Yogyakarta, Indonesia; Division of Radiotherapy, Department of Radiology, Faculty of Medicine, Universitas Gadjah Mada/Dr Sardjito General Hospital, Yogyakarta, Indonesia; Study Program of Specialty in Internal Medicine, Department of Internal Medicine, Faculty of Medicine, Universitas Gadjah Mada, Yogyakarta, Indonesia; Division of Hematology and Medical Oncology, Department of Internal Medicine, Faculty of Medicine, Universitas Gadjah Mada/Dr Sardjito General Hospital, Yogyakarta, Indonesia; Department of Otorhinolaryngology Head and Neck Surgery, Faculty of Medicine, Universitas Gadjah Mada/Dr Sardjito General Hospital, Yogyakarta, Indonesia; Division of Radiotherapy, Department of Radiology, Faculty of Medicine, Universitas Gadjah Mada/Dr Sardjito General Hospital, Yogyakarta, Indonesia; Division of Hematology and Medical Oncology, Department of Internal Medicine, Faculty of Medicine, Universitas Gadjah Mada/DrSardjito General Hospital, Yogyakarta, Indonesia; Department of Otorhinolaryngology Head and Neck Surgery, Faculty of Medicine, Universitas Gadjah Mada/DrSardjito General Hospital, Yogyakarta, Indonesia; Division of Radiotherapy, Department of Radiology, Faculty of Medicine, Universitas Gadjah Mada/DrSardjito General Hospital, Yogyakarta, Indonesia; Division of Hematology and Medical Oncology, Department of Internal Medicine, Faculty of Medicine, Universitas Gadjah Mada/Dr Sardjito General Hospital, Yogyakarta, Indonesia; Department of Anatomy, Faculty of Medicine, Jenderal Soedirman University, Purwokerto, Indonesia; Department of Otorhinolaryngology Head and Neck Surgery, Faculty of Medicine, Universitas Gadjah Mada, Yogyakarta, Indonesia; Division of Radiotherapy, Department of Radiology, Faculty of Medicine, Universitas Gadjah Mada, Yogyakarta, Indonesia; Division of Hematology and Medical Oncology, Department of Internal Medicine, Dr Hasan Sadikin Hospital, Padjadjaran University, Bandung, Indonesia; Genomics Research Center, Academia Sinica, Taipei, Taiwan; Division of Cancer Prevention, National Cancer Institute, Bethesda, MD USA; Department of Otolaryngology, National Taiwan University Hospital and National Taiwan University College of Medicine, Taipei, Taiwan; Department of Head and Neck Surgery, Koo Foundation Sun Yat-Sen Cancer Center, Taipei, Taiwan; Department of Otorhinolaryngology-Head and Neck Surgery China Medical University Hospital, Taichung, Taiwan; Department of Otolaryngology Head & Neck Surgery, Mackay Memorial Hospital, Taipei, Taiwan; Department of Otolaryngology, Far Eastern Memorial Hospital, Taipei, Taiwan; Department of Otolaryngology Head and Neck Surgery, Cathay General Hospital, Taipei, Taiwan; School of Medicine, Fu Jen Catholic University, Taipei, Taiwan; Department of Internal Medicine, National Taiwan University Hospital, Taipei, Taiwan; Health Management Center, National Taiwan University Hospital, Taipei, Taiwan; Graduate Institute of Epidemiology and Preventive Medicine College of Public Health, National Taiwan University, Taipei, Taiwan; Department of Radiation Oncology, Koo Foundation Sun Yat-Sen Cancer Center, Taipei, Taiwan; Division of Cancer Epidemiology and Genetics, National Cancer Institute, Bethesda, MD USA; Department of Otorhinolaryngology Head and Neck Surgery, Faculty of Medicine, Andalas University, Padang, Indonesia; BioMerieux (Shanghai) biotech company limited, Pudong, Shanghai, China; Department of Radiotheraphy, Dharmais National Cancer Hospital, Jakarta, Indonesia; Molecular Biology Laboratory, Faculty of Medicine, Universitas Gadjah Mada, Yogyakarta, Indonesia; Department of Public Health, Faculty of Medicine Universitas Gadjah Mada, Yogyakarta, Indonesia; Department of Otorhinolaryngology Head and Neck Surgery, Faculty of Medicine Universitas Gadjah Mada/Dr Sardjito General Hospital, Yogyakarta, Indonesia; Department of Histology and Cell Biology, Faculty of Medicine Universitas Gadjah Mada, Yogyakarta, Indonesia; Department of Biotechnology, Graduate program, Universitas Gadjah Mada, Yogyakarta, Indonesia; Department of Histology and Biology Molecular, Faculty of Medicine, Univesitas Gadjah Mada, Yogyakarta, Indonesia; Department of Otorhinolaryngology Head and Neck Surgery, Faculty of Medicine Universitas Gadjah Mada/Dr Sardjito General Hospital, Yogyakarta, Indonesia; Department of Otorhinolaryngology Head and Neck Surgery, Dharmais Cancer Hospital, Jakarta, Indonesia; Study Program of Medicine, Faculty of Medicine, Universitas Gadjah Mada, Yogyakarta, Indonesia; Department of Histology and Cell Biology, Faculty of Medicine, Universitas Gadjah Mada, Yogyakarta, Indonesia; Molecular Biology Laboratory, Faculty of Medicine, Universitas Gadjah Mada, Yogyakarta, Indonesia; Department of Clinical Pathology, Faculty of Medicine, Universitas Gadjah Mada, Yogyakarta, Indonesia; Study Program of Medicine, Faculty of Medicine, Universitas Gadjah Mada, Yogyakarta, Indonesia; Department of Histology and Cell Biology, Faculty of Medicine, Universitas Gadjah Mada, Yogyakarta, Indonesia; Study Program of Medicine, Faculty of Medicine, Universitas Gadjah Mada, Yogyakarta, ᅟ; Department of Public Health, Faculty of Medicine, Universitas Gadjah Mada, Yogyakarta, ᅟ; Department of Histology and Cell Biology, Faculty of Medicine, Universitas Gadjah Mada, Yogyakarta, ᅟ; Molecular Biology Laboratory, Faculty of Medicine, Universitas Gadjah Mada, Yogyakarta, ᅟ; Study Program of Medicine, Faculty of Medicine, Universitas Gadjah Mada, Yogyakarta, Indonesia; Department of Histology and Cell Biology, Faculty of Medicine, Universitas Gadjah Mada, Yogyakarta, Indonesia; Division of Hematology and Medical Oncology, Department of Internal Medicine, Faculty of Medicine, Universitas Gadjah Mada/Dr Sardjito General Hospital, Yogyakarta, Indonesia; Study Program of Medicine, Faculty of Medicine, Universitas Gadjah Mada, Yogyakarta, Indonesia; Department of Histology and Cell Biology, Faculty of Medicine, Universitas Gadjah Mada, Yogyakarta, Indonesia; Molecular Biology Laboratory, Faculty of Medicine, Universitas Gadjah Mada, Yogyakarta, Indonesia; Department of Clinical Pathology, Faculty of Medicine, Universitas Gadjah Mada, Yogyakarta, Indonesia; Laboratory of Genetic, Faculty of Biology, Universitas Jenderal Soedirman, Purwokerto, Indonesia; Department of Biology Medicine, Faculty of Medicine, Universitas Indonesia, Jakarta, Indonesia; Department of Anatomic Pathology, Faculty of Medicine, Universitas Indonesia, Jakarta, Indonesia; Department of Otorhinolaryngology Head and Neck Surgery, Dr Cipto Mangunkusumo Hospital, Faculty of Medicine, Universitas Indonesia, Jakarta, Indonesia; Molecular Pathology Unit, Cancer Research Centre, Institute for Medical Research, 50588 Kuala Lumpur, Malaysia; Sarawak Biodiversity Centre, KM20, Semengoh, 93990 Kuching, Sarawak Malaysia; Department of Biotechnology, Universitas Gadjah Mada, Yogyakarta, Indonesia; Dharmais National Cancer Hospital, Jakarta, Indonesia; Department of Otorhinolaryngology Head and Neck Surgery, Faculty of Medicine, Universitas Gadjah Mada, Yogyakarta, Indonesia; Department of Histology and Cell Biology, Faculty of Medicine, Universitas Gadjah Mada, Yogyakarta, Indonesia

## Abstract

A1 Hope and despair in the current treatment of nasopharyngeal cancer

IB Tan

I1 NPC international incidence and risk factors

Ellen T Chang

I2 Familial nasopharyngeal carcinoma and the use of biomarkers

Chien-Jen Chen, Wan-Lun Hsu, Yin-Chu Chien

I3 Genetic susceptibility risk factors for sporadic and familial NPC: recent findings

Allan Hildesheim

I5 Genetic and environmental risk factors for nasopharyngeal cancer in Southeast Asia

James D McKay, Valerie Gaborieau, Mohamed Arifin Bin Kaderi, Dewajani Purnomosari, Catherine Voegele, Florence LeCalvez-Kelm, Graham Byrnes, Paul Brennan, Beena Devi

I6 Characterization of the NPC methylome identifies aberrant epigenetic disruption of key signaling pathways and EBV-induced gene methylation

Li L, Zhang Y, Fan Y, Sun K, Du Z, Sun H, Chan AT, Tsao SW, Zeng YX, Tao Q

I7 Tumor exosomes and translational research in NPC

Pierre Busson, Claire Lhuillier, Olivier Morales, Dhafer Mrizak, Aurore Gelin, Nikiforos Kapetanakis, Nadira Delhem

I8 Host manipulations of the Epstein-Barr virus EBNA1 protein

Sheila Mansouri, Jennifer Cao, Anup Vaidya, and Lori Frappier

I9 Somatic genetic changes in EBV-associated nasopharyngeal carcinoma

Lo Kwok Wai

I10 Preliminary screening results for nasopharyngeal carcinoma with ELISA-based EBV antibodies in Southern China

Sui-Hong Chen, Jin-lin Du, Ming-Fang Ji, Qi-Hong Huang, Qing Liu, Su-Mei Cao

I11 EBV array platform to screen for EBV antibodies associated with NPC and other EBV-associated disorders

Denise L. Doolan, Anna Coghill, Jason Mulvenna, Carla Proietti, Lea Lekieffre, Jeffrey Bethony, and Allan Hildesheim

I12 The nasopharyngeal carcinoma awareness program in Indonesia

Renske Fles, Sagung Rai Indrasari, Camelia Herdini, Santi Martini, Atoillah Isfandiari, Achmad Rhomdoni, Marlinda Adham, Ika Mayangsari, Erik van Werkhoven, Maarten Wildeman, Bambang Hariwiyanto, Bambang Hermani, Widodo Ario Kentjono, Sofia Mubarika Haryana, Marjanka Schmidt, IB Tan

I13 Current advances and future direction in nasopharyngeal cancer management

Brian O’Sullivan

I14 Management of juvenile nasopharyngeal cancer

Enis Ozyar

I15 Global pattern of nasopharyngeal cancer: correlation of outcome with access to radiotherapy

Anne WM Lee

I16 The predictive/prognostic biomarker for nasopharyngeal carcinoma

Mu-Sheng Zeng

I17 Effect of HLA and KIR polymorphism on NPC risk

Xiaojiang Gao, Minzhong Tang, Pat Martin, Yi Zeng, Mary Carrington

I18 Exploring the Association between Potentially Neutralizing Antibodies against EBV Infection and Nasopharyngeal Carcinoma

Anna E Coghill, Wei Bu, Hanh Nguyen, Wan-Lun Hsu, Kelly J Yu, Pei-Jen Lou, Cheng-Ping Wang, Chien-Jen Chen, Allan Hildesheim, Jeffrey I Cohen

I19 Advances in MR imaging in NPC

Ann D King

O1 Epstein-Barr virus seromarkers and risk of nasopharyngeal carcinoma: the gene-environment interaction study on nasopharyngeal carcinoma in Taiwan

Yin-Chu Chien, Wan-Lun Hsu, Kelly J Yu, Tseng-Cheng Chen, Ching-Yuan Lin, Yung-An Tsou, Yi-Shing Leu, Li-Jen Laio, Yen-Liang Chang, Cheng-Ping Wang, Chun-Hun Hua, Ming-Shiang Wu, Chu-Hsing Kate Hsiao, Jehn-Chuan Lee, Ming-Hsui Tsai, Skye Hung-Chun Cheng, Pei-Jen Lou, Allan Hildesheim, Chien-Jen Chen

O2 Familial tendency and environmental co-factors of nasopharyngeal carcinoma: the gene-environment interaction study on nasopharyngeal carcinoma in Taiwan

Wan-Lun Hsu, Kelly J Yu, Yin-Chu Chien, Tseng-Cheng Chen, Ching-Yuan Lin, Yung-An Tsou, Yi-Shing Leu, Li-Jen Liao, Yen-Liang Chang, Tsung-Lin Yang, Chun-Hun Hua, Ming-ShiangWu, Chu-Hsing Kate Hsiao, Jehn-ChuanLee, Ming-Hsui Tsai, Skye Hung-Chun Cheng, Jenq-Yuh Ko, Allan Hildesheim, Chien-Jen Chen

O3 The genetic susceptibility and prognostic role of TERT-CLPTM1L and genes in DNA damage pathways in NPC

Josephine Mun Yee Ko, Wei Dai, Dora Kwong, Wai Tong Ng, Anne Lee, Roger Kai Cheong Ngan, Chun Chung Yau, Stewart Tung, Maria Li Lung

O4 Long term effects of NPC screening

Mingfang Ji, Wei Sheng, Mun Hon Ng, Weimin Cheng, Xia Yu, Biaohua Wu, Kuangrong Wei, Jun Zhan, Yi Xin Zeng, Su Mei Cao, Ningshao Xia, Yong Yuan

O5 Risk prediction of nasopharyngeal carcinoma by detecting host genetic and Epstein-Barr virus variation in saliva

Qian Cui, Miao Xu, Jin-Xin Bei, Yi-Xin Zeng

O6 Patterns of care study in Turkish nasopharyngeal cancer patients (NAZOTURK): A Turkish Radiation Oncology Association Head and Neck Cancer Working Group Study

B Şahin, A Dizman, M Esassolak, A Saran İkizler, HC Yıldırım, M Çaloğlu, B Atalar, F Akman, C Demiroz, BM Atasoy, E Canyilmaz, S Igdem, G Ugurluer, T Kütük, M Akmansoy, E Ozyar

O7 Long term outcome of intensity modulated radiotherapy in nasopharyngeal carcinoma in National Cancer Centre Singapore

Kiattisa Sommat, Fu Qiang Wang, Li-Lian Kwok, Terence Tan, Kam Weng Fong, Yoke Lim Soong, Shie Lee Cheah, Joseph Wee

O8 International phase II randomized study on the addition of docetaxel to the combination of cisplatin and 5-fluorouracil in the induction treatment for nasopharyngeal carcinoma in children and adolescents

M Casanova, E Özyar, C Patte, D Orbach, A Ferrari, VF Cristine, H Errihani, J Pan, L Zhang, S Liji, K Grzegorzewski, L Gore, A Varan

O9 Prognostic impact of metastatic status in patients with nasopharyngeal carcinoma

Susanna Hilda Hutajulu, Guntara Khuzairi, Camelia Herdini, Henry Kusumo, Mardiah Suci Hardianti, Kartika Widayati Taroeno-Hariadi, Ibnu Purwanto, Johan Kurnianda

O10 Development of small molecule inhibitors of latent Epstein-Barr virus infection for the treatment of nasopharyngeal carcinoma

Troy E. Messick, Kimberly Malecka, Lois Tolvinski, Samantha Soldan, Julianna Deakyne, Hui Song, Antonio van den Heuvel, Baiwei Gu, Joel Cassel, Mark McDonnell, Garry R Smith, Venkata Velvadapu, Haiyan Bian, Yan Zhang, Marianne Carlsen, Shuai Chen, Alastair Donald, Christian Lemmen, Allen B Reitz, Paul M Lieberman

O11 Therapeutic targeting of cancer stem-like cells using a Wnt modulator, ICG-001, enhances the treatment outcome of EBV-positive nasopharyngeal carcinoma

King Chi Chan, Lai Sheung Chan, Kwok Wai Lo, Timothy Tak Chun Yip, Roger Kai Cheong Ngan, Michael Kahn, Maria Li Lung, Nai Ki Mak

O12 Role of micro-RNA in NPC biology

Fei-Fei Liu

O13 Expansion of EBNA1- and LMP2-specific effector T lymphocytes from patients with nasopharyngeal carcinoma without enhancement of regulatory T cells

Wafa Khaali; Juliette Thariat; Laurence Fantin; Flavia Spirito; Meriem Khyatti; El Khalil Ben Driss; Sylvain Olivero; Janet Maryanski; Alain Doglio

O14 The experience of patients’ life after amifostine radiotherapy treatment (ART) for nasopharyngeal carcinoma (NPC)

Mengxue Xia, Yunfei Xia, Hui Chang, Rachel Shaw

O15 Analysis of mitochondrial DNA mutation in latent membrane protein-1 positive nasopharyngeal carcinoma

Pudji Rahaju

O16 Factors influencing treatment adherence of nasopharyngeal cancer and the clinical outcomes: a hospital-based study

Mardiah Suci Hardianti, Sindhu Wisesa, Kartika Widayati Taroeno-Harijadi, Ibnu Purwanto, Bambang Hariwiyanto, Wigati Dhamiyati, Johan Kurnianda

O17 Chromosomal breaks mediated by bile acid-induced apoptosis in nasopharyngeal epithelial cells: in relation to matrix association region/scaffold attachment region

Sang-Nee Tan, Sai-Peng Sim

O18 Expression of *p53* (wild type) on nasopharyngeal carcinoma stem cell that resistant to radiotherapy

Muhtarum Yusuf, Ahmad C Romdhoni, Widodo Ario K, Fedik Abdul Rantam

O19 Mathematical model of nasopharyngeal carcinoma in cellular level

Sugiyanto, Lina Aryati, Fajar Adi-Kusumo, Mardiah Suci Hardianti

O20 Differential expression of microRNA-21 on nasopharyngeal carcinoma plasma patient

SY Bintoro, R Oktriani, C. Herawati, A Surono, Sofia M. Haryana

O21 Therapeutic targeting of an oncogenic fibroblast growth factor-FGF19, which promotes proliferation and induces EMT of carcinoma cells through activating ERK and AKT signaling

L. Zhong, L. Li, B. B. Ma, A. T. Chan, Q. Tao

O22 Resist nasopharyngeal carcinoma (NPC): next generation T cells for the adoptive immunotherapy of NPC

M. Kalra, M. Ngo, S. Perna, A. Leen, N. Lapteva, C. M. Rooney, S. Gottschalk

O23 The correlation of heat shock protein 70 expressions and staging of nasopharyngeal carcinoma

Elida Mustikaningtyas, Sri Herawati, Achmad C Romdhoni

O24 Epstein-Barr virus serological profiles of nasopharyngeal carcinoma - A tribute to Werner Henle

Mingfang Ji, YaruiXu, Weimin Cheng, ShengxiangGe, Fugui Li, M. H. Ng

O25 Targeting the apoptosis pathway using combination TLR3 agonist with anti-survivin molecule (YM-155) in nasopharyngeal carcinoma

Louise SY Tan, Benjamin Wong, CM Lim

O26 The resistance mechanism of nasopharyngeal cancer stem cells to cisplatin through expression of CD44, Hsp70, p53 (wild type), Oct-4, and ß-catenin encoded-genes

Achmad C Romdhoni, Fedik A. Rantam, Widodo Ario Kentjono

P1 Prevalence of nasopharyngeal carcinoma patients at Departement of Otorhinolaringology-Head and Neck Surgery, Dr. Hasan Sadikin general hospital, Bandung, Indonesia in 2010-2014

Deasy Z Madani, Nur Akbar, Agung Dinasti Permana

P2 Case report on pediatric nasopharyngeal carcinoma at Dr. Sardjito Hospital, Yogyakarta

Camelia Herdini, Sagung Rai Indrasari, Jajah Fachiroh, Dwi Hartati, T. Baning Rahayudjati

P3 Report on loco regionally advanced nasopharyngeal cancer patients treated with induction chemotherapy followed by concurrent chemo-radiation therapy

Iswandi Darwis, Susanna Hilda Hutajulu, Bambang Hariwiyanto, Wigati Dhamiyati, Ibnu Purwanto, Kartika Widayati Taroeno-Hariadi, Johan Kurnianda

P4 Sex and age differences in the survival of patients with nasopharyngeal carcinoma

Sindhu Wisesa, Mardiah Suci Hardianti, Susanna Hilda Hutajulu, Kartika Widayati Taroeno-Harijadi, Ibnu Purwanto, Camelia Herdini, Wigati Dhamiyati, Johan Kurnianda

P5 Impact of delayed diagnosis and delayed therapy in the treatment outcome of patients with nasopharyngeal carcinoma

Khoirul Anwar, Susanna Hilda Hutajulu, Sagung Rai Indrasari, Sri Retna Dwidanarti, Ibnu Purwanto, Kartika Widayati Taroeno-Hariadi, Johan Kurnianda

P6 Anaysis of pretreatment anemia in nasopharyngeal cancer patients undergoing neoadjuvant therapy

Dominicus Wendhy Pramana, Susanna Hilda Hutajulu, Bambang Hariwiyanto, Wigati Dhamiyati, Ibnu Purwanto, Kartika Widayati Taroeno-Hariadi, Johan Kurnianda

P7 Results of treatment with neoadjuvant cisplatin-5FU in locally advanced nasopharyngeal carcinoma: a local experience

Diah Ari Safitri, Susanna Hilda Hutajulu, Camelia Herdini, Sri Retna Dwi Danarti, Ibnu Purwanto, Kartika Widayati Taroeno-Hariadi, Johan Kurnianda

P8 Geriatrics with nasopharyngeal cancer

Suryo A Taroeno, Sindhu Wisesa, Kartika Widayati Taroeno-Hariadi, Ibnu Purwanto, Bambang Hariwiyanto, Wigati Dhamiyati, Johan Kurnianda

P9 Correlation of lymphocyte to monocyte and neutrophil to lymphocyte ratio to the response of cisplatin chemoradiotheraphy in locally advance nasopharyngeal carcinoma

I. Wijaya, A. Oehadian, D. Prasetya

P10 Prediction of nasopharyngeal carcinoma risk by Epstein-Barr virus seromarkers and environmental co-factors: the gene-environment interaction study on nasopharyngeal carcinoma in Taiwan

Wan-Lun Hsu, Yin-Chu Chien, Kelly J Yu, Cheng-Ping Wang, Ching-Yuan Lin, Yung-An Tsou, Yi-Shing Leu, Li-Jen Liao, Yen-Liang Chang^191,192^, Jenq-Yuh Ko, Chun-Hun Hua, Ming-Shiang Wu, Chu-Hsing Kate Hsiao, Jehn-Chuan Lee, Ming-Hsui Tsai, Skye Hung-Chun Cheng, Pei-Jen Lou, Allan Hildesheim, Chien-Jen Chen

P11 Non-viral risk factors for nasopharyngeal carcinoma in West Sumatra, Indonesia

Sukri Rahman, Bestari J. Budiman, Novialdi, Rahmadona, Dewi Yuri Lestari

P12 New prototype Vidas EBV IgA quick: performance on Chinese and Moroccan populations

C. Yin, A. Foussadier, E. Blein, C. Chen, N. Bournet Ammour, M. Khiatti, S. Cao

P13 The expression of EBV-LMP1 and VEGF as predictors and plasma EBV-DNA levels as early marker of distant metastasis after therapy in nasopharyngeal cancer

Dewi Syafriyetti Soeis Marzaini

P14 Characteristics and factors influencing subjects refusal for blood samples retrieval: lesson from NPC case control study in Yogyakarta – Indonesia

Dwi Hartati, Baning Rahayujati, Camelia Herdini, Jajah Fachiroh

P15 Expression of microRNA BART-7-3p and mRNA PTEN on blood plasma of patients with nasopharyngeal carcinoma

L. Gunawan, S. Mubarika Haryana, A. Surono, C. Herawati

P16 IgA response to native early antigen (IgA-EAext) of Epstein-Barr virus (EBV) in healthy population and nasopharyngeal carcinoma (NPC) patients: the potential for diagnosis and screening tools

Michael Hartono, Jajah Fachiroh, Umi Intansari, Dewi Kartikawati Paramita

P17 IgA responses against Epstein-Barr Virus Early Antigen (EBV-EA) peptides as potential candidates of nasopharyngeal carcinoma detection marker

Akmal Akbar, Jajah Fachiroh, Dewi Kartikawati Paramita

P18 Association between smoking habit and IgA-EBV titer among healthy individuals in Yogyakarta, Indonesia

Benny Hermawan, T Baning Rahayudjati, Dewi K Paramita, Jajah Fachiroh

P19 Epstein-Barr virus IgA titer comparison of healthy non-family individuals and healthy first degree family of NPV patients

Gabriella Argy, Jajah Fachiroh, Dewi Kartikawati Paramita, Susanna Hilda Hutajulu

P20 Identification of EBV Early Antigen (EA) derived peptides for NPC diagnosis

Theodora Caroline Sihotang, Jajah Fachiroh, Umi Intansari, Dewi Kartikawati Paramita

P21 Host-pathogen study: relative expression of mRNA BRLF1 Epstein-Barr virus as a potential biomarker for tumor progressivity and polymorphisms of *TCRBC* and *TCRGC2* host genes related to genetic susceptibility on nasopharyngeal carcinoma

Daniel Joko Wahyono, Purnomo Soeharso, Dwi Anita Suryandari, Lisnawati, Zanil Musa, Bambang Hermani

P22 *In vitro* efficacy of silvestrol and episilvestrol, isolated from Borneo, on nasopharyngeal carcinoma, a major cancer in Borneo

Maelinda Daker, Yeo Jiun Tzen, Norhasimah Bakar, Asma’ Saiyidatina Aishah Abdul Rahman, Munirah Ahmad, Yeo Tiong Chia, Alan Khoo Soo Beng

P23 The expression of mir-141 in patients with nasopharyngeal cancer

Widyandani Sasikirana, Tirta Wardana, Muhammad Radifar, Cita Herawati, Agus Surono, Sofia Mubarika Haryana

## A1 Hope and despair in the current treatment of nasopharyngeal cancer

### IB Tan^1,2^ (i.tan@nki.nl)

#### ^1^The Netherlands Cancer Institute, Amsterdam, The Netherlands; ^2^Faculty of Medicine, Universitas Gadjah Mada/Dr Sardjito General Hospital, Yogyakarta, Indonesia

Nasopharyngeal carcinoma (NPC) is a rare disease in the Western world, but it is endemic in certain parts of Southeast Asia and China. During the last decades, the outcome of the treatment has improved considerably. Concurrent chemo-radiation has become the treatment of choice in advanced NPC. Five years survival figures of 80-90 % for T1-T2 and 60-80 % for T3-T4 are not exceptional. The relation between NPC and the Epstein Barr virus (EBV), which is almost 100 % in endemic areas, has opened up several possibilities for early detection and monitoring of the disease using EBV based markers or to new treatment strategies based on this viral connection.

The main progress in the treatment of NPC is coming from the top end hospitals in the high endemic regions. Effective treatment schedules are applied and compliance with treatment and follow up are secured. The good news is the fact that the incidence of NPC is also declining in more prosperous cities like Hong Kong and Singapore and the big cities in Taiwan. So, apparently, preventive measures can be effective on the long term.

Unfortunately, NPC is correlated to hygiene and environmental circumstances like pollution in areas where those being treated live, the adding of carcinogens to food and even to candies and it is more common in developing countries where many patients are suffering from this disease. Furthermore, the treatment possibilities in these parts of the endemic regions are less favorable. Due to the shortness of radiotherapy capacity and the lack of adequate insurance systems, many patients do not have access to effective treatment for this disease.

In a recent study in a tertiary referral hospital in Yogyakarta, the treatment results appeared to be behind compared with the international literature. Waiting time for radiotherapy was during the years of the study (2009-2013) between 3 and 4 months, but has increased to over 1 year and 9 months in March 2015, probably due to the introduction of the National Healthcare program in Indonesia. The most obvious solutions would be to increase the number of RT devices.

However, the short supply is so serious that at least 10 to 20 years will pass before the capacity will be in line with the demand. In the meantime patients are suffering and dying of this treatable disease. Action is needed and preventive measures have to be taken.

Future research should focus on innovative treatment strategies like photodynamic therapy, which have been proven to be effective in residual or recurrent NPC. A combination with other novel treatments, like vaccination and immunotherapy is challenging.

## I1 NPC international incidence and risk factors

### Ellen T Chang^1,2^ (echang@exponent.com)

#### ^1^Health Sciences Practice, Exponent, Inc., California, USA; ^2^Division of Epidemiology, Department of Health Research and Policy, Stanford University School of Medicine, California, USA

The international geographic distribution of NPC is unique among malignancies. Incidence rates can exceed 20 per 100,000 person-years among males and 10 per 100,000 person-years among females in southern China. Intermediate rates are observed in Southeast Asia, North Africa, the Middle East, and the Arctic, as well as among Asian and Pacific Islander migrant populations, while rates generally remain below 1 per 100,000 throughout the rest of the world. The age-specific incidence rate peaks at around 45-59 years in high-incidence areas, whereas a small early incidence peak at ages 15-19 years is followed by a later peak at around 65-79 years in low-incidence areas. The male-to-female incidence ratio is consistently around 2-3 or greater. Much of the striking geographic variation may be attributable to certain risk factors, such as certain *HLA* alleles and consumption of Chinese-style salted fish, whose geographic distribution mirrors that of NPC. Other NPC risk factors, such as EBV infection, are ubiquitous, yet are almost certainly modified by geographically heterogeneous co-factors. Most established risk factors for NPC are associated with undifferentiated NPC, although tobacco smoking is more strongly associated with squamous cell NPC. The recent worldwide decline in NPC incidence, especially in high-incidence regions, points to an important role of modifiable environmental risk factors that could serve as targets for further disease prevention.

## I2 Familial nasopharyngeal carcinoma and the use of biomarkers

### Chien-Jen Chen^1,2^, Wan-Lun Hsu^1^, Yin-Chu Chien^1^

#### ^1^Genomics Research Center, Academia Sinica, Taipei, Taiwan; ^2^Graduate Institute of Epidemiology and Preventive Medicine College of Public Health, National Taiwan University, Taipei, Taiwan

##### **Correspondence:** Chien-Jen Chen (chencj@gate.sinica.edu.tw) – Genomics Research Center, Academia Sinica, Taipei, Taiwan

Nasopharyngeal carcinoma (NPC) has a striking geographical variation with the highest incidence in South China, Southeast Asia, and East Africa; and the lowest incidence in Europe, West Africa, and Central America. As infection of Epstein-Barr virus (EBV), a well-documented cause of NPC, is ubiquitous; the geographical variation of NPC is likely attributable to ethnic and environmental cofactors. Chinese ethnicity in almost all cancer registries in the world has the highest incidence of NPC. Both case-control and cohort studies has demonstrated a strong familial tendency of NPC, suggesting common environmental and genetic components shared by family members may play a role in the development of NPC. Several molecular and genomic biomarkers for the early detection or long-term risk prediction of nasopharyngeal carcinoma have been reported previously. Elevated serotiter of antibodies against EBV including anti-EBV VCA IgA, anti-EBV DNase, anti-EBNA1 and the elevated serum level of EBV DNA (viral load) are the most important biomarkers for the detection and prediction of NPC. Genetic polymorphisms of xenobiotic metabolism enzymes CYP2E1, DNA repair enzymes hOGG1 and XRCC1, and human leukocyte antigen (HLA) are also associated with NPC risk. Among various risk calculators for NPC, those incorporating EBV biomarkers have the highest area under the receiver operating curve (AUROC). The AUROC remains low for risk calculators including only family NPC history and/or genetic polymorphisms, suggesting familial tendency and genetic susceptibility may not have validity (sensitivity and specificity) high enough for the early detection or risk prediction of NPC.

## I3 Genetic susceptibility risk factors for sporadic and familial NPC: recent findings

### Allan Hildesheim (hildesha@exchange.nih.gov)

#### Infections & Immunoepidemiology Branch, DCEG, NCI, Bethesda MD, USA

Along with Epstein-Barr virus infection, genetic susceptibility is believed to play an important role in the development of NPC. Early studies of NPC genetics used targeted candidate gene approaches. Those studies were successful at identifying Human Leukocyte Antigen (HLA) genes as important determinants of NPC risk but findings were largely inconsistent with respect to other genes hypothesized to be involved in NPC pathogenesis. Technological advances have allowed for more comprehensive approaches to query genetic factors linked to NPC development, using SNP-based genotyping and, more recently, next generation sequencing approaches. Both sporadic and familial NPC studies have been conducted or are underway using these newer technologies. In this presentation, findings and ongoing efforts to characterize genetic risk factors for sporadic and familial NPC will be presented and discussed. Most of these studies have been conducted by collaborative groups, including the International NPC Genetics Working Group for sporadic NPC and a NCI-Taiwan collaboration for familial NPC.

## I5 Genetic and environmental risk factors for nasopharyngeal cancer in Southeast Asia

### James D McKay^1^, Valerie Gaborieau^1^, Mohamed Arifin Bin Kaderi^1,2^, Dewajani Purnomosari^1,3^, Catherine Voegele^1^, Florence LeCalvez-Kelm^1^, Graham Byrnes^1^, Paul Brennan^1^, Beena Devi^4^

#### ^1^International Agency for Research on Cancer, Lyon, France; ^2^International Islamic University Malaysia, Kuantan, Pahang, Malaysia; ^3^Faculty of Medicine, Universitas Gadjah Mada, Yogyakarta, Indonesia; ^4^Umum Hospital Sarawak, Sarawak, Malaysia

##### **Correspondence:** James D McKay (MckayJ@iarc.fr) – International Agency for Research on Cancer, Lyon, France

Incidence rates of nasopharyngeal cancer (NPC) vary markedly across the world. In Sarawak, Malaysia incidence rates differ importantly between ethnic subgroups. This presentation will discuss both genetic and environmental explanations for these incidence rates differences. Germ-line exome sequencing within 12 NPC patients from a Bidayuh pedigree identified haplo type sharing within the major histo-compatability complex region on human chromosome 6. However, it does not appear completely explain the segregation of NPC in the pedigree. By contrast, important associations were noted with consumption of preserved foods, for example frequent heavy consumers of salted fish had an over 4-fold risk of NPC relative compared to never consumers (p = 9x10^-20^). These associations were also more marked within higher incidence rates ethnic subgroups. These results suggest that environmental exposures, rather than genetic susceptibility, appear more likely to explain the heterogeneous incidence rates in this region.

## I6 Characterization of the NPC methylome identifies aberrant epigenetic disruption of key signaling pathways and EBV-induced gene methylation

### Li L^1,2^, Zhang Y^1,2^, Fan Y^1,2^, Sun K^1,2^, Du Z^1,2^, Sun H^1,2^, Chan AT^1,2^, Tsao SW^1,2^, Zeng YX^1,2^, Tao Q^1,2^

#### ^**1**^Cancer Epigenetics Laboratory, Department of Clinical Oncology, State Key Laboratory of Oncology in South China, Sir YK Pao Center for Cancer, Li Ka Shing Institute of Health Sciences, The Chinese University of Hong Kong, Sha Tin, Hong Kong; ^2^Department of Anatomy, The University of Hong Kong, Pok Fu Lam, Hong Kong

##### **Correspondence:** Tao Q (qtao@cuhk.edu.hk) – Cancer Epigenetics Laboratory, Department of Clinical Oncology, State Key Laboratory of Oncology in South China, Sir YK Pao Center for Cancer, Li Ka Shing Institute of Health Sciences, The Chinese University of Hong Kong

NPC is a major tumor prevalent in South China including Hong Kong and Southeast Asia, as the top cancer for males of 20-44 years of age. Current genomic sequencing studies found only rare mutations in NPC, indicating its critical epigenetic etiology. NPC is virtually 100 % associated with the infection of herpesvirus Epstein-Barr virus (EBV), which is a strong epigenetic driver inducing more CpG methylation in infected cells. Although multiple genetic/genomic studies have shown numerous alterations of cancer genes in NPC, no epigenome study is available for NPC yet, except for some single-gene-based epigenetic studies. We profiled the whole-genome CpG methylation (methylomes) of NPC cell lines, primary tumors and normal nasopharyngeal epithelial cells (including EBV-infected ones) using methylated DNA immunoprecipitation (MeDIP). We observed extensive, genome-wide methylation of cellular genes, with methylation of multiple known and novel genes verified. In total, we identified ~1000-2000 methylated genes with promoter CGIs in cell lines and primary tumors. Moreover, EBV-induced cellular gene methylation was also identified. Epigenetic disruption of key signaling pathways including Wnt, MAPK, TGF-beta, Hedgehog and ErbB signaling was detected through bioinformatics analysis. Frequent methylation of Wnt signaling regulators including *SFRPs, DACTs, DKKs, WNT5A, CDH11* and *ROR2* was detected in cell lines and primary tumors. Further functional studies showed that these genes are *bona fide* tumor suppressor genes (TSG) for NPC. TSG methylation could also be detected in nasal swab and serum samples from NPC patients, hence of potential biomarker value. Thus, NPC methylome shows a special high-degree CpG methylation epigenotype, similar to the EBV-infected gastric cancer, indicating a critical role of epigenetic alterations in NPC pathogenesis.

## I7 Tumor exosomes and translational research in NPC

### Pierre Busson^1^, Claire Lhuillier^1^, Olivier Morales^2^, Dhafer Mrizak^2^, Aurore Gelin^1^, Nikiforos Kapetanakis^1^, Nadira Delhem^2^

#### ^1^Université Paris-sud, CNRS UMR8126 and Gustave Roussy, Villejuif, France; ^2^ Université de Lille, CNRS UMR 8161 and Institut Pasteur de Lille, Lille, France

##### **Correspondence:** Pierre Busson (pierre.busson@igr.fr) – Université Paris-sud, CNRS UMR8126 and Gustave Roussy, Villejuif, France

Exosomes are extra-cellular nanovesicles of 50 to 100 nm in diameter which are produced by all types of eukaryotic cells including cancer cells and which play a key role in cellular communications. Prior to their extra-cellular release, exosomes are formed in endosomal organelles called multivesicular bodies. Proteins carried by exosomes are contained in their peripheral lipid bilayer or their internal cavity. It is convenient to classify these proteins in three sets: a) structural proteins common to all exosomes regardless of producing cells (for example CD63); b) proteins related to a specific lineage of producing cells (for example CD227 or CD326 for epithelial cells), c) proteins linked to a specific physiological condition (for example TSAP6 in cells undergoing genotoxic stress).

Various nucleic acids are consistently carried by exosomes: microRNAs, messenger RNAs, long non-coding RNAs and probably DNA. There are now multiple evidences of horizontal genetic transfers mediated by exosomes *in vitro* and *in vivo*. Exosomes are involved in various aspects of tumor biology, especially immune suppression, preparation of the metastatic niche and angiogenesis. There are three major reasons to investigate exosomes in NPC patients: their probable contribution to oncogenesis, especially viral oncogenesis, their role in immune suppression and their potential as a source of biomarkers. For example, there is indirect evidence that LMP1 carried by exosomes play a role in tumor angiogenesis. The immunosuppressive functions of NPC exosomes have been recently documented *in vitro* as well as in murine models.

Regarding the development of biomarkers, it is interesting to note that specific capture of NPC tumor exosomes can be achieved from plasma samples on the basis of their surface expression of HLA class II molecules. One aim of future investigations will be to determine whether exosome capture can improve detection of viral nucleic acids in the peripheral blood (especially viral microRNAs).

## I8 Host manipulations of the Epstein-Barr virus EBNA1 protein

### Sheila Mansouri, Jennifer Cao, Anup Vaidya, Lori Frappier

#### Department of Molecular Genetics, University of Toronto, Toronto, Ontario, Canada

##### **Correspondence:** Lori Frappier (lori.frappier@utoronto.ca) – Department of Molecular Genetics, University of Toronto, Toronto, Ontario, Canada

EBNA1 is the only EBV protein expressed in all EBV-induced tumours. It contributes to latent EBV infection in multiple ways including manipulation of the cellular environment in order to promote cell survival. One way that EBNA1 manipulates cells is by binding and hijacking specific host proteins, including casein kinase 2 (CK2), known to regulate multiple pathways important for cell proliferation. EBNA1 binds CK2 through its regulatory (β) subunit and can relocalize CK2 to promyleocytic leukemia (PML) nuclear bodies where CK2 phosphorylation of PML proteins leads to loss of the nuclear bodies, resulting in impairment of apoptosis and DNA repair. Using a proteomic approach, we identified an uncharacterized cellular protein (ARKL1) as binding CK2β through the same binding pocket as EBNA1, suggesting that EBNA1 could interfere with ARKL1 function by blocking its binding to CK2. One function we have identified for ARKL1 and CK2β is in inhibiting the EBV lytic cycle. Therefore the interplay between EBNA1, ARKL1 and CK2 may be one way of regulating EBV reactivation. We also used miRNA cloning coupled with high throughput sequencing, to investigate the effects of EBNA1 on cellular miRNAs in NPC cells. EBNA1 was found to upregulate multiple let-7 family miRNAs, including let-7a. Consistent with these effects, the let-7a target, Dicer, was decreased by EBNA1, and reporter assays indicated that this effect was dependent on the let-7a target sequences in the Dicer 3′UTR. Previous reports indicate that low Dicer levels promote tumour metastases and the epithelial-to-mesenchymal transition (EMT), and that EBNA1 induces EMT in NPC cell lines. Therefore our results suggest that EBNA1 might induce EMT by lowering Dicer. In addition, Dicer has been implicated in promoting EBV reactivation. Accordingly, we found that a let-7a mimic inhibited EBV reactivation to the lytic cycle while a let-7 sponge increased reactivation. These results provide a mechanism by which EBNA1 promotes EBV latency by inducing let-7 miRNAs.

## I9 Somatic genetic changes in EBV-associated nasopharyngeal carcinoma

### Lo Kwok Wai (kwlo@cuhk.edu.hk)

#### The Chinese University of Hong Kong, Sha Tin, Hong Kong

Nonkeratinizing nasopharyngeal carcinoma (NPC) is an epithelial malignancy driven by Epstein-Bar virus (EBV) infection and cumulative genetic and epigenetic changes. Both viral and cellular genes contribute to disease pathogenesis and their interplay creates distinct phenotypes, unique molecular features and tumor microenvironment. Although few recurrent mutations in common cancer-related genes were reported in the recent genomic studies, the contributions of several unique somatic genetic changes in NPC tumorigenesis will be discussed.

Our molecular and functional studies have revealed the high frequency of genetic alterations in p16/CyclinD1 pathway and their critical role in supporting stable EBV infection in NPC. In stably EBV-infected cells, constitutive activation of multiple signalling pathways by either latent viral oncoproteins, such as LMP1, or somatic changes plays a crucial role for driving transformation. Activation of distinct NF-κB signals, p50/p50/BCL3 and/or p50/RelB, are consistently found in EBV-positive NPC tumor lines and primary tumors. Notably, recurrent somatic changes in multiple components (e.g. *LTBR, TRAF3, TRAF2, NFKBIA, A20*) of NF-κB were detected in the tumors, especially that with weak or absence of LMP1 expression. These mutational events enhance cell survival and modulate tumor microenvironment via constitutively activated NF-κB signals. The unique finding highlights the importance of inflammation in NPC tumorigenesis. Furthermore, inactivating TRAF3 by LMP1 and somatic mutations may allow NPC cells to evade immune response against abundant EBV-encoded transcripts such as *EBER*s. On the other hand, by next generation sequencing, we recently identified a number of fusion transcripts in a panel of EBV-positive NPC tumor lines. Among these gene rearrangements, a novel fusion gene *UBR5-ZNF423* is recurrently detected in >8 % of primary tumors. Notably, the growth of NPC cells with *UBR5-ZNF423* rearrangement is dependent on expression of this fusion gene. The transforming ability of *UBR5-ZNF423* fusion was also confirmed by in vitro and in vivo studies. Constitutive expression of *UBR5-ZNF423* in NIH3T3 fibroblasts significantly enhanced its anchorage-independent growth in soft agar and induced tumor formation in a nude mouse model. These findings suggest that expression of UBR5-ZNF423 might contribute to the transformation of a subset of NPCs, possibly by altering the activity of early B cell factors (EBFs).

Finally, it is believed that comprehensive characterization of the NPC genome by multiple sequencing platforms might unveil more critical genetic changes contributed to the progression of this EBV-associated epithelial malignancy.

## I10 Preliminary screening results for nasopharyngeal carcinoma with ELISA-based EBV antibodies in Southern China

### Sui-Hong Chen, Jin-lin Du, Ming-Fang Ji, Qi-Hong Huang, Qing Liu, Su-Mei Cao

#### Department of Cancer Prevention Research, Sun Yat-sen University Cancer Center; State Key Laboratory of Oncology in Southern China, Guangzhou 510060 Guangdong, P.R. China

##### **Correspondence:** Su-Mei Cao (caosm@sysucc.org.cn) – Department of Cancer Prevention Research, Sun Yat-sen University Cancer Center; State Key Laboratory of Oncology in Southern China, Guangzhou 510060 Guangdong, P.R. China

A cluster randomized nasopharyngeal carcinoma (NPC) mass screening trial using a combination of immunoglobulin A antibodies against Epstein-Barr virus capsid antigen (VCA/IgA) and nuclear antigen-1 (EBNA1/IgA) by enzyme-linked immunosorbent assay (ELISA) has been conducted in Zhongshan and Sihui cities in Southern China. The residences aged 30-59 years in the 8 screening towns of the 2 cities were invited to participate in this screening trial from 2008. Until the end of 2012, a total of 45,005 participants were recruited. In this study, we showed NPC cancer occurrence and the early detection rates in the population of the screening towns compared with those in the control towns. After more than 1 year follow-up, 89 NPC cases (with 4 cases unknown clinical stage) were detected in all of the screened participants, 76 cases (with 16 cases unknown clinical stage) were found in the nonparticipants in one screened town and 69 (with 8 cases unknown clinical stage) in one control town. The corresponding early detection rates in the screened participants (63/85, 74.1 %) was 3.5 times higher than that in the population of the control town (13/61, 21.3 %), p < 0.001. In the screened towns, about 36.9 % target participants took part in the screening trial and the early detection rates in the screened participants was also higher than the non-participants (13/60, 21.7 %), p < 0.001. Moreover, most of the NPC patients (71/89, 80.0 %) were detected in the high risk group, next in the medium risk group (12/89, 13.5 %) and the lowest in the lowest risk group. In the 28,688 screened participants with follow-up more than 3 years, the serial detection for NPC cases was also analyzed annually. The results showed that 60 % NPC cases (61.7 %, 42/70) were detected in the first year follow-up; however, the cancers were rare in the low risk group in the 3 years of follow-up. It suggested that NPC screening with ELISA-based EBV antibodies could increase the early detection rate of NPC in the screening group and further follow-up is needed to examine whether screening had an effect on decreasing mortality for NPC in endemic areas.

## I11 EBV array platform to screen for EBV antibodies associated with NPC and other EBV-associated disorders

### Denise L. Doolan^1^, Anna Coghill^2^, Jason Mulvenna^1^, Carla Proietti^1^, Lea Lekieffre^1^, Jeffrey Bethony^3^, and Allan Hildesheim^2^

#### ^1^Infectious Diseases Program, QIMR Berghofer Institute of Medical Research, Brisbane, QLD, Australia; ^2^Division of Cancer Epidemiology and Genetics, National Cancer Institute, Bethesda, MD, USA; ^3^Department of Microbiology and Tropical Medicine, George Washington University, Washington DC, USA

##### **Correspondence:** Denise L. Doolan (Denise.Doolan@qimr.edu.au) – Infectious Diseases Program, QIMR Berghofer Institute of Medical Research, Brisbane, QLD, Australia

Nasopharyngeal carcinoma (NPC) is a squamous cell carcinoma which is sensitive to treatment when detected early, but early detection of NPC has proven difficult due the location of the tumor and the lack of obvious clinical signs. Hence, there is a need for a sensitive and specific biomarker for early stage or occult NPC. Epstein Barr Virus (EBV) has been implicated as an active player in NPC pathogenesis. Elevated IgA antibodies to a number of EBV antigens occur in the vast majority of individuals with frank NPC, and antibody titers tend to rise with tumor burden and correlate strongly with tumour remission and recurrence. Simplex ELISAs targeting anti-EBV EBNA1 and VCA IgA are highly “sensitive” to the presence of the tumor. In fact, efforts are underway in some regions to evaluate EBNA1/VCA IgA screening for the early detection of NPC. Predicted specificity of these tests, however, is not optimal. We hypothesize that a whole proteome microarray approach can be applied to identify a multiplexed antibody signature to EBV as a sensitive and specific biomarker for NPC. To test this hypothesis, we constructed a novel protein microarray that contains the entire EBV proteome, represented by approximately 90 proteins derived from publically available prototypical EBV type 1 and type 2 genome sequences as well as known splice variants of those proteins (approximately 200 protein fragments in total). This proteome microarray is being probed with well-characterized serum samples from histologically confirmed NPC cases and community-matched controls. Using comprehensive feature selection analysis, we hope to identify an EBV antibody signature that effectively discriminates between NPC case and control subjects. Such an antibody signature could be applied to screen populations resident in areas where the incidence of NPC is high for potential therapeutic intervention.

## I12 The nasopharyngeal carcinoma awareness program in Indonesia

### Renske Fles^1^, Sagung Rai Indrasari^2^, Camelia Herdini^2^, Santi Martini^3^, Atoillah Isfandiari^3^, Achmad Rhomdoni^4^, Marlinda Adham^5^, Ika Mayangsari^5^, Erik van Werkhoven^6^, Maarten Wildeman^7^, Bambang Hariwiyanto^2^, Bambang Hermani^5^, Widodo Ario Kentjono^4^, Sofia Mubarika Haryana^8^, Marjanka Schmidt^9^, IB Tan^1,2^

#### ^1^Department of Head and Neck Surgery, Netherlands Cancer Institute, Amsterdam, The Netherlands; ^2^Department of Otorhinolaryngology Head and Neck Surgery, Faculty of Medicine, Universitas Gadjah Mada/Dr Sardjito General Hospital, Yogyakarta, Indonesia; ^3^Department of Public Health, Faculty of Medicine, Universitas Airlangga, Surabaya, Indonesia; ^4^Department of Otorhinolaryngology Head and Neck Surgery, Faculty of Medicine, Universitas Airlangga, Surabaya, Indonesia; ^5^Department of Otorhinolaryngology Head and Neck Surgery, Faculty of Medicine, Universitas Indonesia, Jakarta, Indonesia; ^6^Department of Biometrics, Netherlands Cancer Institute, Amsterdam, The Netherlands; ^7^Department of Head and Neck Surgery, Amsterdam Medical Center, Amsterdam, The Netherlands; ^8^Department of Histology and Cell Biology, Faculty of Medicine, Universitas Gadjah Mada, Yogyakarta, Indonesia; ^9^Division of Psychosocial Research and Epidemiology, Division of Molecular Pathology, Netherlands Cancer Institute, Amsterdam, The Netherlands

##### **Correspondence:** Renske Fles (r.fles@nki.nl) – Department of Head and Neck Surgery, Netherlands Cancer Institute, Amsterdam, The Netherlands

**Introduction** Nasopharyngeal cancer (NPC) is common in Indonesia. Nevertheless, most people are diagnosed with an advanced stage of disease resulting in poor treatment outcome. Late stage diagnosis of these patients is partly due to limited knowledge of the early symptoms of NPC among health care workers in the primary health care centers (PHCC) and the referral system for NPC suspects. We established the NPC awareness program, which aims to educate general practitioners (GPs), midwives and nurses working in the PHCC. The aim of this study was to evaluate the efficiency of this program on the short- and long-term improvement of knowledge and referral of NPC patients by PHCC staff.

**Methods** The awareness program consisted of twelve symposia, organized in Yogyakarta, Jakarta and East Java. They contained lectures about the early symptoms and the risk factors of NPC, practical examination and the referral system for NPC suspects. A representative GP from each PHCC was invited to participate. Based on the Train The Trainer (TTT) principle, GPs received training material and were obligated to train their colleagues in the PHCC. Before and after a training (symposium or TTT), all participants completed a questionnaire. After one and a half years, all PHCC in the province of Yogyakarta were visited again to assess the long-term effects of the training.

**Results** In total 703 GPs attended the symposia and trained 1349 staff members: 314 other GPs, 685 nurses and 350 midwives. After the training, respondents’ average score regarding the knowledge of NPC symptoms increased from 47 points (out of 100) to 74 (p < 0.001). There was no difference between symposium and TTT (p = 0.880). Two years after the training, the knowledge remained significantly increased, at 59 points (p < 0.001). Overall, GPs scored better compared to nurses (-18 points) and midwives (-20 points) on questions regarding symptoms. Similar results were found for knowledge regarding NPC risk factors.

**Discussion** The initial results of this NPC awareness program provide proof that the NPC awareness program is effective also in the long term and therefore should continue. The effect of the improved knowledge based on the stage at diagnoses of the NPC patients will be scrutinized. In addition, patient-delay, doctors-delay and system-delay causing the poor treatment outcome should be investigated in more detail.

## I13 Current advances and future direction in nasopharyngeal cancer management

### Brian O’Sullivan (brian.osullivan@rmp.uhn.on.ca)

#### University of Toronto, Toronto, Canada

The management of primary loco regionally advanced nasopharyngeal cancer (NPC) is fraught with technical challenges for safe and effective treatment due to its location in the sanctuary of the skull base; great opportunity exists for tumor extension to regions of relative inaccessibility. The latter makes radiotherapy (RT) the most obvious choice for initial management compared to surgery, since the disease is particularly radiosensitive.

The immediate proximity of critical structures (e.g. brain stem, spinal cord and the optic apparatus) to target areas harboring disease in the locally advanced setting represents the most important barriers of delivery of curative RT with conventional technique. Ability to avoid critical anatomy using precision techniques to plan, guide, and deliver RT is paramount for this disease. Intensity-modulated radiotherapy (IMRT) is currently the most usual precision RT approach. The technical advances provided by IMRT enable conformation of tumoricidal doses delivered to concave shaped distributions, thereby providing, for the first time, the opportunity for safe delivery of high doses to tumor while protecting normal tissues. The value of IMRT in eliminating blindness and protecting salivary function as well as enhancing disease control has been demonstrated in randomized trials. In parallel with IMRT, emerging imaging modalities (MRI and PET CT) have also improved accuracy in target delineation. The implementation of volumetric image-guidance (IGRT) during radiotherapy has further improved setup accuracy and provides an opportunity for monitoring tumor volume and normal tissue changes over the course of radiotherapy with the eventual promise of ‘real-time’ dose-volume parameter adaption to anatomic structures when necessary. Recently, particle therapy, such as intensity-modulated proton therapy (IMPT), has emerged as an additional potentially attractive option for this disease. However, the clinical benefit and cost-effectiveness remain to be evaluated.

In parallel to advances in RT technique, progress has also been made combining RT with systemic therapy. Since the proven benefit of chemotherapy in enhancing RT loco regional effect and reducing risk of distant metastasis was demonstrated in Inter group-0099, combined RT with chemotherapy has become standard of care for loco-regionally advanced NPC. However, controversies exist regarding the sequence of chemotherapy, what agents to use, and which sub group of patients benefit. Several clinical trials testing the benefit of induction chemotherapy in addition to concurrent chemoradiotherapy have been completed, and others are exploring a risk stratified approach by selecting patients using post-radiotherapy EBV DNA titers for additional adjuvant chemotherapy. Network meta-analysis of published clinical trial data may also provide additional evidence in guiding optimal treatment for NPC.

## I14 Management of juvenile nasopharyngeal cancer

### Enis Ozyar (enis.ozyar@acibadem.com.tr)

#### Department of Radiation Oncology, Acibadem University School of Medicine, Maslak, Istanbul, Turkey

Nasopharyngeal carcinoma (NPC) is a rare disease in children with distinct epidemiological and etiological features, histopathological characteristic, and clinical presentation. NPC shows a bimodal age distribution in non-endemic countries and the first peak is seen in the second decade in addition to the second peak at more advanced age. Juvenile NPC (JNPC) is one of the few malignancies in childhood that emerge from the epithelium, and constitutes 1-5 % of all pediatric cancers and 20-50 % of all primary nasopharyngeal malignant tumors in children. The incidence of JNPC varies widely around the world, and both genetic and environmental factors contribute to the development of the disease. In the United States, only 3 % of NPC occurs in patients younger than 19 years old according to SEER data. JNPC almost always have the undifferentiated variant of the disease, which is associated with advanced locoregional spread and distant metastases. While the concomitant chemotherapy and radiation, with or without adjuvant chemotherapy, is the current standard for adult patients with NPC, neoadjuvant chemotherapy with radiotherapy has gained popularity parallel to other pediatric treatment protocols in various solid tumours. The optimal radiotherapy dose for disease control in JNPC has reportedly ranged from 50 to 70 Gy in the literature.

There are few prospective studies in the literature on the management of JNPC. Currently, cisplatin-based induction chemotherapy followed by high-dose radiotherapy is the treatment of choice. Although multimodality treatment has increased the 5-year survival to 70-90 %, late morbidity is a major concern. Immune-modulation with interferon has resulted with excellent outcome, and studies have been extended to investigate the impact of immunotherapy on survival, in combination with less toxic chemoradiotherapy. Integration of new chemotherapeutic agents or modifiying their administration sequence when managing JNPC’s is another way to improve results. A randomized phase II study recently compared two versus three-drug regimen including docetaxel in 72 JNPC. The results of this study did not show any benefit. It was shown that intensity-modulated radiotherapy (IMRT) provides superior target coverage and normal tissue sparing compared with conventional radiotherapy in adult patients with NPC. However, compared with conventional radiotherapy, a two-fold increase in integral dose has been theoretically estimated with the use of IMRT due to the larger treatment volumes. In the pediatric setting the risk could be significant due to a higher inherent susceptibility of tissues. However, as the risk of secondary cancers related with IMRT estimated to be 2 % compared with 1 % for CRT, it seems logical to utilize IMRT in JNPC in order to decrease the significant late effects. Although the optimal treatment modality for JNPC has not been established, any potential reduction in radiation field and doses is desirable due to the significant chronic morbidity among long-term survivors.

## I15 Global pattern of nasopharyngeal cancer: correlation of outcome with access to radiotherapy

### Anne WM Lee (awmlee@ha.org.hk)

#### University of Hong Kong – Shenzhen Hospital, Shenzhen, Guangdong, China

Epidemiological data on nasopharyngeal cancer (NPC) from Globocan 2012 showed that the total number of new cases in the world amounted to 86,691 and number of deaths was 50,828. NPC has a uniquely skewed geographic distribution, 81 % of new cases occurred in Asia and 9 % in Africa. The top four countries (China, Indonesia, Vietnam and India) accounted for 64 % of the global burden. China has the largest number of new cases (n = 33,198), but in terms of age-standardized incidence (ASI), China only ranked 19^th^ because only the southern provinces have high incidence.

Radiotherapy (RT) is the primary treatment modality for NPC. We conducted a study on the correlation of outcome with access to RT to evaluate the fundamental requirement for RT facilities. Outcome for each country was calculated by taking [1-(age-standardized modality/incidence)] as a proxy for 5-year relative survival (RS). Information from the IAEA Directory of Radiotherapy Centres (DIRAC) was extracted to calculate the number of RT equipment (linear accelerator and/or Cobalt machine) and radiation oncologists per million populations.

There were 112 countries with both outcome and RT data: the proxy RS rate ranged widely from 0 to 83 % (median 50 %). Countries can be categorized into poor (proxy RS < 50 %), median (proxy RS = 50 %), and good outcome group (proxy RS > 50 %). Among the 53 countries with poor outcome, the top 14 countries accounted for 75 % of the global burden; their proxy RS rate ranged 22-41 %. Univariate linear regression showed a significant correlation between outcome and the availability of RT: proxy RS increased at 3.4 % (p < 0.001) and 1.5 % (p = 0.001) per unit increase in RT equipment and oncologist per million, respectively. Comparison of median similarly showed statistically significant differences in RT indicators among the three outcome groups (p < 0.001). The median number of RT equipment per million populations increased from 0.6 in the poor to 4.5 in the good outcome group; the corresponding number of oncologists increased from 1.1 to 7.1. These findings will be useful in advocating cancer plan by government, particularly those in developing countries.

Nasopharyngeal cancer is a highly treatable cancer. For patients treated with good quality RT in experienced centers, 5-year disease-specific survival exceeds 80 %. Concerted global efforts are urgently needed to improve outcome in countries with poor outcome.

## I16 The predictive/prognostic biomarker for nasopharyngeal carcinoma

### Mu-Sheng Zeng (zengmsh@mail.sysu.edu.cn)

#### State Key Laboratory of Oncology in South China; Collaborative Innovation Center for Cancer Medicine, Sun Yat-sen University Cancer Center, Guangzhou, China

Nasopharyngeal carcinoma (NPC) is a malignant tumor originated from nasopharyngeal epithelial cells. The cancer is an Epstein–Barr virus (EBV)-associated malignancy, with a remarkable racial and geographical distribution. Radiotherapy (RT) is the primary treatment; while concurrent chemoradiotherapy with or without adjuvant chemotherapy is the standard of care for advanced NPC. Although the 5-year overall survival was up to 70 % in IMRT era, predicting metastasis, recurrence, prognosis and therapeutic response of NPC remain a challenge. Therefore, discovery of biomarkers for predicting treatment failure of NPC is of great importance for guiding NPC treatment and improving patients’ prognosis. Thanks to the advancements in proteomics and bioinformatics in the recent 20 years, a great amount of biomarker that correlates NPC clinical parameters were identified in DNA, RNA and protein level, respectively. All these biomarkers were correlated with recurrence, distant metastasis, or prognosis for NPC patients. Elevated levels of circulating cell-free Epstein–Barr virus (EBV) DNA have been detected in plasma and serum samples from nasopharyngeal cancer (NPC) patients by quantitative real time PCR (qPCR) test. This qPCR test for circulating EBV DNA was found to be useful in the clinical management of NPC patients. Currently, plasma EBV DNA has gradually being adopted in clinical applications and is considered to be the most attractive potential biomarker to complement the TNM classification. Many other biomarkers, such as serum C-reactive protein (hs-CRP), lactate dehydrogenase (LDH), Cystatin A, and serum amyloid A (SAA), was associated with recurrence and prognosis for NPC patients. However, now incorporating biomarker into new staging systems is still a challenge for NPC patients. Even for plasma EBV DNA, test variation among laboratories should be addressed and the test of plasma EBV DNA should be globally standardized before widely accepted by clinicians. Therefore, to identify more reliable NPC biomarkers, new strategies for biomarker screening need to be adopted, and clinical validation and test of identified biomarkers in a large series of samples and multicenter validation should be performed. A simple blood test for evaluation of disease progress and therapeutic response individually is expected to be a reality in the future.

## I17 Effect of HLA and KIR polymorphism on NPC risk

### Xiaojiang Gao^1^, Minzhong Tang^2,3^, Pat Martin^1^, Yi Zeng,^2^ Mary Carrington^1^

#### ^1^Cancer & Inflammation Program, National Cancer Institute, Frederick, Maryland, USA; ^2^College of Life Sciences, Beijing University of Technology, Beijing, China; ^3^Wuzhou Red Cross Hospital, Guangxi, China

##### **Correspondence:** Xiaojiang Gao (gaoxia@mail.nih.gov) – Cancer & Inflammation Program, National Cancer Institute, Frederick, Maryland, USA

Southern China has recorded one of the highest rates of incidence of NPC. The risk of NPC in China has long been associated with the HLA polymorphism. We have also detected protective as well as susceptible HLA factors in a southern Chinese NPC cohort from Guangxi. In the present study we extended the genetic analysis to HLA interaction with KIR. The study cohort included three groups from Guangxi: 1405 NPC cases, 1362 healthy individuals who are negative for EBV/IgA/VCA (a precursor for NPC onset), and 1288 healthy individuals who are positive for EBV/IgA/VCA. Four-digit HLA typing was performed using SBT. The KIR profiles (presence and absence of KIR genes) were examined using SSP. HLA-A*11:01 was confirmed to be the major projective allele (OR = 0.58, p < 0.0001). Another A*11 subtype A*11:02, on the other hand, showed no effect on NPC risk despite the fact that the two A*11 subtypes differ from each other by a single amino acid in position 19 which is outside the peptide binding groove and therefore unlikely to influence peptide presentation. However, this single replacement is known to influence A*11 interaction with KIR2DS4 with A*11:02 serving as a better ligand than A*11:01. The analysis of compound HLA/KIR genotypes showed that the presence or absence of KIR2DS4 did not change the protective effect of A*11:01 but affected A*11:02 association with NPC development and EBV/IgA/VCA conversion. When KIR2DS4 is absent A*11:02 showed an increased riskfor NPC onset (OR = 1.94, p < 0.005). It is especially true when the comparison was between NPC cases and the EBV/IgA/VCA positive controls (OR = 5.3, p < 0.0001). A borderline significance of the same compound genotype was also detected between the two control groups of EBV/IgA/VCA negative vs. positive individuals (OR = 0.25, p = 0.002). In other words in the absence of the KIR2DS4 gene individuals having A*11:02 are less likely to convert to EBV/IgA/VCA positive but among those EBV/IgA/VCA positive individuals the A*11:02^+^/KIR2DS4^-^ genotype confers an increased risk for NPC onset. These results indicate that HLA/KIR interaction might indeed play a role in NPC pathogenesis.

## I18 Exploring the Association between Potentially Neutralizing Antibodies against EBV Infection and Nasopharyngeal Carcinoma

### Anna E Coghill^1†^, Wei Bu^2†^, Hanh Nguyen ^2^, Wan-Lun Hsu^3.4^, Kelly J Yu^1,5^, Pei-Jen Lou^6^, Cheng-Ping Wang^6^, Chien-Jen Chen^3,4^, Allan Hildesheim^1†^, Jeffrey I Cohen^2†^

#### ^1^Infections and Immunoepidemiology Branch, Division of Cancer Epidemiology and Genetics, National Cancer Institute, Bethesda, Maryland, USA; ^2^Laboratory of Infectious Diseases, National Institute of Allergy and Infectious Diseases, Bethesda, Maryland, USA; ^3^Graduate Institute of Epidemiology, College of Public Health, National Taiwan University, Taipei, Taiwan; ^4^Genomics Research Center, Academia Sinica, Taipei, Taiwan; ^5^Division of Cancer Prevention, National Cancer Institute, Bethesda, Maryland, USA; ^6^Department of Otolaryngology, National Taiwan University Hospital and College of Medicine, Taipei, Taiwan

##### **Correspondence:** Anna E Coghill (anna.coghill@nih.gov) – Infections and Immunoepidemiology Branch, Division of Cancer Epidemiology and Genetics, National Cancer Institute, Bethesda, Maryland, USA

^†^Both authors contributed equally to this work.

**Introduction** Epstein-Barr virus (EBV) is a necessary co-factor for the development of nasopharyngeal carcinoma (NPC). Strong evidence exists that antibody titers to latent and lytic-phase EBV proteins are elevated years prior to NPC diagnosis, which has led to their ongoing evaluation as high-risk NPC biomarkers. However, biomarkers that can prospectively identify individuals at lower risk of developing NPC have not been identified. Antibodies against proteins necessary for EBV cell entry (i.e., antibodies that could neutralize virus infection) represent a biologically plausible starting point to look for such low-risk factors.

**Aim** We utilized the Taiwan Family Study (TFS) to evaluate the association between risk of developing NPC and potentially neutralizing antibodies against B-cell infection (EBV glycoprotein gp350 [gp350]) and epithelial cell infection (glycoprotein H/glycoprotein L [gH/gL]).

**Methods** The TFS is a prospective cohort of 2,557 individuals recruited from 358 Taiwanese multiplex families (i.e., >2 family members affected by NPC). Incident NPC cases were identified passively (linkage to national tumor registry) and actively (clinical evaluation) from 1996-2010 (N = 21). We also selected a sample of frequency matched (age, gender) individuals who remained disease-free during follow-up (N = 50). Potentially neutralizing antibodies were measured in baseline serum utilizing an immunoprecipitation assay. Baseline serum was also tested for neutralizing potential against EBV infection of B-cells and epithelial cells utilizing a rapid flow cytometry-based neutralization assay. Finally, samples from prevalent NPC cases (N = 30 early stage; 10 late stage) were evaluated for gp350 and gH/gL antibodies.

**Results** Average baseline gp350 antibody levels were significantly lower (x = 24.10; p-value = 0.048) in those who developed NPC during follow-up (median = 5 years) compared to individuals who remained disease-free (x = 53.48). Similarly, serum from incident NPC cases neutralized EBV infection of B-cells less potently (raw IC_50_ = 93.98) than disease-free individuals (raw IC_50_ = 110.5), a statically significant difference when considering NPC diagnosed >1 year after baseline (p-value = 0.026). Patterns differed according to the target cell type, with no observed differences in the average baseline gH/gL antibody level or neutralizing potential against EBV infection of epithelial cells according to NPC status during follow-up. Elevated gp350 and gH/gL antibody levels were detected at the time of NPC diagnosis (i.e., prevalent disease), regardless of disease stage.

**Conclusions** Our results suggest that high-risk individuals with lower gp350 antibody titers, which may neutralize EBV B-cell infection, are more likely to develop incident NPC. If the pattern for this vaccine-candidate antibody is replicated, it could provide an avenue for future research into a previously-unidentified, *low-risk* NPC factor. The patterns of this protective marker, however, differ between incidence and prevalent NPC cases, and the gp350 antibody may therefore not be useful for NPC risk stratification in early detection screening programs.

## I19 Advances in MR imaging in NPC

### Ann D King (king2015@cuhk.edu.hk)

#### The Chinese University of Hongkong, Sha Tin, China

**Advances in MR imaging in NPC**

MRI is a versatile imaging modality providing both anatomical and functional information in the same examination. MRI is one of the most widely used modalities for imaging NPC and this lecture will focus on some of the recent advances in MRI.

**Advances in anatomical MRI**

Detection of NPC

Nasopharyngeal MRI is a very sensitive technique for the detection of NPC and it detects small NPCs buried in the pharyngeal recess or beneath the mucosa that cannot be visualized by endoscopy. Therefore, MRI has a role in the investigation of patients with suspected NPC who have normal endoscopic findings, such as those patients with elevated plasma EBV DNA. In these patients MRI can identify the site of cancer for biopsy, while those patients without cancer can be spared sampling biopsies. MRI also identifies a range of benign hyperplastic changes in the nasopharynx, including adenoidal hyperplasia, which may cause false positive findings for NPC at endoscopy.

**Advances in functional MRI**

General

The head and neck is a technically challenging area in which to perform functional MRI. In addition there are variations in methods for extracting data from the functional maps, and correlation of functional data with treatment response is often based on only short-term clinical follow-up. However, despite these drawbacks results, functional MRI show promise in several areas of NPC evaluation. This lecture will discuss very briefly some of these functional techniques (DCE, MRS, CEST and ASL), but will focus mainly on diffusion weighted imaging (DWI) which shows the greatest potential for clinical use in head and neck cancer.

DWI

NPC primary tumours and metastatic nodes show restriction of diffusion which can be seen as high signal on the b-1000 DWI images and low signal on the apparent diffusion coefficient (ADC) map. Currently results from the small number of NPC research papers have shown a possible role for DWI in the following areas; *(1) Tumour characterisation.* Differences have been shown between the ADC of NPC and the ADC of other head and neck cancers, benign nodes, nasopharyngeal lymphoid hyperplasia and skull base infection; *(2) Pretreatment prediction of treatment response.* Sophisticated methods of ADC analysis using the distribution of ADC values are promising for predicting long term treatment response; *(3) Intra-treatment monitoring*. This is an area where DWI appears to have the greatest potential in cancer management. Over the course of RT/CRT the ADC rises as tumour cells die. Results from both squamous cell carcinoma and NPC suggest that a greater % rise in ADC in the first few weeks of treatment indicates a more favourable response. (4) *Identification of post-treatment residual cancer*. Residual cancers show restricted diffusion similar to that of the pretreatment tumour, and the ADC values of residual cancers are lower than those of post treatment inflammatory masses. Research from squamous cell carcinoma suggests that visual analysis of the DWI images + ADC maps (residual cancer = high signal on b-1000 DWI + low signal on the ADC map), may be superior to the measurement of ADC values.

## O1 Epstein-Barr virus seromarkers and risk of nasopharyngeal carcinoma: the gene-environment interaction study on nasopharyngeal carcinoma in Taiwan

### Yin-Chu Chien^1^, Wan-Lun Hsu^1^, Kelly J Yu^2^, Tseng-Cheng Chen^3,4^, Ching-Yuan Lin^5^, Yung-An Tsou^6^, Yi-Shing Leu^7^, Li-Jen Laio^8^, Yen-Liang Chang^9,10^, Cheng-Ping Wang^3,4^, Chun-Hun Hua^6^, Ming-Shiang Wu^11,12^, Chu-Hsing Kate Hsiao^13^, Jehn-Chuan Lee^7^, Ming-Hsui Tsai^6^, Skye Hung-Chun Cheng^14^, Pei-Jen Lou^3,4^, Allan Hildesheim^15^, Chien-Jen Chen^1,13^

#### ^1^Genomics Research Center, Academia Sinica, Taipei, Taiwan; ^2^Division of Cancer Prevention, National Cancer Institute, Bethesda, MD, USA; ^3^Department of Otolaryngology, National Taiwan University Hospital; ^4^National Taiwan University College of Medicine, Taipei, Taiwan; ^5^Department of Head and Neck Surgery, Koo Foundation Sun Yat-Sen Cancer Center, Taipei, Taiwan; ^6^Department of Otorhinolaryngology-Head and Neck Surgery China Medical University Hospital, Taichung, Taiwan; ^7^Department of Otolaryngology-Head & Neck Surgery, Mackay Memorial Hospital, Taipei, Taiwan; ^8^Department of Otolaryngology, Far Eastern Memorial Hospital, Taipei, Taiwan; ^9^Department of Otolaryngology Head and Neck Surgery, Cathay General Hospital, Taipei, Taiwan; ^10^School of Medicine, Fu Jen Catholic University, Taipei, Taiwan; ^11^Department of Internal Medicine, National Taiwan University Hospital, Taipei, Taiwan; ^12^Health Management Center, National Taiwan University Hospital, Taipei, Taiwan; ^13^Graduate Institute of Epidemiology and Preventive Medicine College of Public Health, National Taiwan University, Taipei, Taiwan; ^14^Department of Radiation Oncology, Koo Foundation Sun Yat-Sen Cancer Center, Taipei, Taiwan; ^15^Division of Cancer Epidemiology and Genetics, National Cancer Institute, Bethesda, MD, USA

##### **Correspondence:** Yin-Chu Chien (ycchien219@gate.sinica.edu.tw) – Genomics Research Center, Academia Sinica, Taipei, Taiwan

**Introduction** Epstein-Barr virus (EBV) has been suggested as the strongest risk factor for nasopharyngeal carcinoma (NPC). We conducted a multi-center case-control study in Taiwan to assess the independent effect of two EBV infection markers and interaction with other environmental factors for newly developed NPC.

**Aims** This study aims to evaluate anti-EBV VCA IgA and anti-EBV EA-EBNA1 IgA with other risk factors on risk of NPC development.

**Methods** A total of 1,656 histologically confirmed NPC cases, including of 896 prospective cases and 760 retrospective cases, and 1,608 community controls were recruited between July 2010 and December 2014 in Taiwan. Six hundred and ninety-nine (699) NPC incidence cases and 1,359 age-gender matched controls with adequate blood samples and questionnaires were enrolled in the analysis. Information on socio-demographic characteristics, cigarette smoking, betel quid chewing, alcohol consumption, diet history, medical history, and occupational history were collected by a standardized interviewed questionnaire. Anti-EBV VCA IgA and anti-EBNA1 IgA were tested in all blood samples. Unconditional logistic regression analysis was used to estimate odds ratio (ORadj) with 95 % confidence interval (CI) after adjusting for education years and ethnicity.

**Result and discussion** Two EBV seromarkers were associated with an increased NPC risk showing OR_adj_ (95 % CI) of 57.0 (42.1-77.0, P < 0.001) for anti-EBV VCA IgA, and 91.3 (62.5-127.8, P < 0.001) for anti-EBV EA-EBNA1 IgA. Compared to seronegative subjects, the OR_adj_ was 12.9 (8.3-20.0) for seropositivity of either anti-EBV VCA IgA or anti-EBV EA-EBNA1 IgA, and 648.9 (377.5-999) for those who were seropositive on both EBV seromarkers. In the stratification analysis, the risk were higher for subjects who were seropositive on both EBV seromarkers and without family history of NPC (OR_adj_ = 719.6, 95 % CI: 401.2-999 for subjects without family history vs. OR_adj_ = 314.6, 95 % CI: 56.1-999 for subjects with family history). Compared to never smokers, smokers with seropositive on both EBV seromarkers had higher risk for NPC (OR_adj_ = 922.5, 95 % CI: 370.8-999 for smokers vs. OR_adj_ = 534.2, 95 % CI: 268.3-999 for never smokers). Among seropositive subjects on both EBV seromarkers, the NPC risk was lower for those with high intake of milk, deep-sea fish, dark vegetables, and fresh fruit.

## O2 Familial tendency and environmental co-factors of nasopharyngeal carcinoma: the gene-environment interaction study on nasopharyngeal carcinoma in Taiwan

### Wan-Lun Hsu^1^, Kelly J Yu^2^, Yin-Chu Chien^1^, Tseng-Cheng Chen^3^, Ching-Yuan Lin^4^, Yung-An Tsou^5^, Yi-Shing Leu^6^, Li-Jen Liao^7^, Yen-Liang Chang^8^, Tsung-Lin Yang^3^, Chun-Hun Hua^5^, Ming-ShiangWu^9^, Chu-Hsing Kate Hsiao^10^, Jehn-ChuanLee^6^, Ming-Hsui Tsai^5^, Skye Hung-Chun Cheng^11^, Jenq-Yuh Ko^3^, Allan Hildesheim^12^, Chien-Jen Chen^1,10^

#### ^1^Genomics Research Center, Academia Sinica, Taipei, Taiwan; ^2^Division of Cancer Prevention, National Cancer Institute, Bethesda, MD, USA; ^3^Department of Otolaryngology, National Taiwan University Hospital and National Taiwan University College of Medicine, Taipei, Taiwan; ^4^Department of Head and Neck Surgery, Koo Foundation Sun Yat-Sen Cancer Center, Taipei, Taiwan; ^5^Department of Otorhinolaryngology-Head and Neck Surgery China Medical University Hospital, Taichung, Taiwan; ^6^Department of Otolaryngology-Head & Neck Surgery, Mackay Memorial Hospital, Taipei, Taiwan; ^7^Department of Otolaryngology, Far Eastern Memorial Hospital, Taipei, Taiwan; ^8^Department of Otolaryngology Head and Neck Surgery, Cathay General Hospital, Taipei, Taiwan; School of Medicine, Fu Jen Catholic University, Taipei, Taiwan; ^9^Department of Internal Medicine, National Taiwan University Hospital, Taipei, Taiwan; Health Management Center, National Taiwan University Hospital, Taipei, Taiwan; ^10^Graduate Institute of Epidemiology and Preventive Medicine College of Public Health, National Taiwan University, Taipei, Taiwan; ^11^Department of Radiation Oncology, Koo Foundation Sun Yat-Sen Cancer Center, Taipei, Taiwan; ^12^Division of Cancer Epidemiology and Genetics, National Cancer Institute, Bethesda, Bethesda MD, USA

##### **Correspondence:** Wan-Lun Hsu (lun0112@ms26.hinet.net) – Genomics Research Center, Academia Sinica, Taipei, Taiwan

**Introduction** Epstein-Barr virus (EBV) is a well-documented etiological agent in the development of nasopharyngeal carcinoma (NPC). EBV is known to infect the vast majority of adults worldwide (~95 %), usually with lifelong persistence. However, only a small fraction of EBV infected individuals are expected to develop NPC in their lifetime. Various co-factors have been postulated to be important determinants of NPC.

**Aim** The study objective is to evaluate putative environmental exposures and NPC.

**Methods** A total of 1,656 histologically confirmed NPC cases, comprised of 896 prospective cases and 760 retrospective cases, and 1,608 community controls were recruited between July 2010 and December 2014. Controls were frequency matched to the cases on age and gender. A standardized interviewed questionnaire was administered by trained nurses to all subjects. Information on socio-demographic characteristics, cigarette smoking, betel quid chewing, alcohol consumption, diet history, medical history, and occupational history were included. Unconditional logistic regression analysis was used to estimate odds ratio (OR_adj_) with 95 % confidence interval (CI) after adjusting for education years and ethnicity.

**Resultand discussion** Compared to never smokers, current smokers had an adjusted OR (OR_adj_) of 1.39 (95 % CI = 1.19-1.62) for NPC. Subjects with a family history of NPC had an increased risk of NPC compared to those without such a family history (OR_adj_ = 3.77, 95 % CI = 2.84-5.02). Drinking milk (OR_adj_ = 0.69, 95 % CI = 0.56-0.86 for the intake > = 2 times per week versus never), intake of dark vegetables (OR_adj_ = 0.45, 95 % CI = 0.33-0.61 for intake >1 times/week vs. <=4 times per month), consumption of deep-sea fish (OR_adj_ = 0.45, 95 % CI = 0.33-0.61 for intake > =5 times per week vs. <=3 times per month), and intake of fresh fruit (OR_adj_ = 0.49, 95 % CI = 0.39-0.60 for >1 time per week vs <1 time per week) were all inversely associated with NPC risk.

## O3 The genetic susceptibility and prognostic role of TERT-CLPTM1L and genes in DNA damage pathways in NPC

### Josephine Mun Yee Ko^1^, Wei Dai^1^, Dora Kwong^1,2,3,^, Wai Tong Ng^3,4^, Anne Lee^3,5^, Roger Kai Cheong Ngan^3,6^, Chun Chung Yau^3,7^, Stewart Tung^3,8^ and Maria Li Lung^1,2,3^

#### ^1^Department of Clinical Oncology, University of Hong Kong, Pokfulam, Hong Kong SAR, People’s Republic of China; ^2^Center for Cancer Research, University of Hong Kong, Pokfulam, Hong Kong SAR, People’s Republic of China; ^3^Center for Nasopharyngeal Carcinoma Research, University of Hong Kong, Pokfulam, Hong Kong SAR, People’s Republic of China; ^4^Department of Clinical Oncology, Pamela Youde Nethersole Eastern Hospital, Chai Wan, Hong Kong SAR, People’s Republic of China; ^5^Department of Clinical Oncology, Hong Kong University-Shenzhen Hospital, Shenzhen, People’s Republic of China; ^6^Department of Clinical Oncology, Queen Elizabeth Hospital, Kowloon, Hong Kong SAR, People’s Republic of China; ^7^Department of Oncology, Princess Margaret Hospital, Kwai Chung, Hong Kong SAR, People’s Republic of China; ^8^Department of Clinical Oncology, TuenMun Hospital, TuenMun, Hong Kong SAR, People’s Republic of China

##### **Correspondence:** Josephine Mun Yee Ko (joko@hku.hk) – Department of Clinical Oncology, University of Hong Kong, Pokfulam, Hong Kong SAR, People’s Republic of China

**Introduction** The genetic etiology of nasopharyngeal carcinoma (NPC) remains unclear. We hypothesized that heritable NPC risk was attributable to the cumulative effects of multiple common low-risk variants. A multigenic pathway-based approach was used to systematically score the cumulative effects with SNPs in genes in DNA repair pathways for NPC risk. Although the radiotherapy is very effective in curing NPC patients with early- stage tumors, local failure and distant metastasis remain major challenges for treatment and it is necessary to identify prognostic biomarkers that predict poor clinical outcomes of advanced-stage NPC patients.

**Aims** We aimed to identify genetic susceptibility genes involved in NPC development and prognostic biomarkers for NPC patients.

**Methods** A case-control association study was performed with Mass ARRAY genotyping of about 300 SNPs covering 161 genes/loci in 2,349 Hong Kong individuals including 1,177 NPC patients and 1,172 Red Cross individuals. Both single SNP and pathway based analysis were performed for NPC risk. Patients were also stratified according to their familial status for meaningful association analysis. For identification of the prognostic biomarkers of NPC, the clinical pathological information of 1,177 NPC was collected for univariate and multivariate statistical analysis for survival.

**Results and discussions** Single SNP association identified three SNPs (rs401681, rs6774494, and rs3757318) corresponding to TERT/CLPTM1L, MDS1-EVI1, and CCDC170 conferring modest protective effects individually for NPC risk by the logistic regression analysis. Stratification of NPC cases according to familial status identified rs2380165 in BLM with an association with family history-positive (FH+) NPC patients. Multiple SNP pathway-based analysis observed cumulative effects for increasing numbers of unfavorable genotypes in TERT- CLPTM1L and double-strand break repair (DSBR) conferred elevated risk in FH+ and sporadic NPC patients. Our preliminary data suggested a SNP in p53 with prognostic value for survival of NPC patients. The prognostic role of p53 in NPC should be further validated in a larger cohort.

## O4 Long term effects of NPC screening

### Mingfang Ji^1,2,3^, Wei Sheng^1,2,3^, Mun Hon Ng^1,2,3^, Weimin Cheng^1,2,3^, Xia Yu^1,2,3^, Biaohua Wu^1,2,3^, Kuangrong Wei^1,2,3^, Jun Zhan^1,2,3^, Yi Xin Zeng^1,2,3^, Su Mei Cao^1,2,3^, Ningshao Xia^1,2,3^, Yong Yuan^1,2,3^

#### ^1^Cancer Research Institute of Zhongshan, Zhongshan, Guangdong province, 528403, China; ^2^National Institute of Diagnostics and Vaccine Development in Infectious Disease, State Key Laboratory of Molecular Vaccinology and Molecular Diagnostics, School of Public Health, Xiamen University, Xiamen 361005, China; ^3^Sun Yat-sen University Cancer Center, 651 Dongfeng Road East, Guangzhou 510060, China

##### **Correspondence:** Mingfang Ji (jmftbh@sina.com) – Cancer Research Institute of Zhongshan, Zhongshan, Guangdong province, 528403, China

**Introduction** Mass nasopharyngeal carcinoma (NPC) screening using Epstein-Barr Virus (EBV) antibody as the primary screening test was found to effect early diagnosis, but the long term effect of which has not been ascertained.

**Aim** We conducted long term effects of NPC screening in a high incidence area to assess the efficacy of NPC screening.

**Method** 16,695 residents of one town in Zhongshan City of southern China aged 30 to 59 years in the screening arm and 33,390 residents in two neighboring towns in the study arm matched for age, gender and date of entry to study with each study subject at 2:1 ratio were enrolled over 12 months since Aug 2009. A study subject initially screened with medium EBV antibody levels and higher was invited for retesting annually in the following 3 years and a subject initially screened or retested and found to have high antibody levels was referred to otorhinolaryngologists for diagnostic workup for NPC.

**Result and discussion** Forty-one (n = 41) NPC cases were diagnosed among study subjects and 45 among the control subjects over a median follow-up of 52 months. Based on subjects referred for diagnostic workup, the sensitivity of the screening triage was 94.9 % (0.827, 0.994), specificity was 94.3 % (0.940, 0.946), the positive predictive value was 3.25 % (0.022, 0.043) and negative predictive value was 99.99 % (0.9997, 1.000). The cumulative incidence of the participants was 0.246 %, 1.8 times that of the control subjects. Most of the excessive diagnosis was probably because of detection of future cases. This conferred on the population 92 % protection against NPC for at least 5 years. The proportion of cases diagnosed with stage I & II disease was increased from 20 % to 78 % and the overall survival rate was increased from 97.6 % to 82.2 %.

**Conclusion** We conclude that the NPC screening could confer 92 % protection against NPC for 5 years and reduce death risk by 88.4 % (0.05, 0.986).

## O5 Risk prediction of nasopharyngeal carcinoma by detecting host genetic and Epstein-Barr virus variation in saliva

### Qian Cui, Miao Xu, Jin-Xin Bei, Yi-Xin Zeng

#### Sun Yat-sen University Cancer Center; State Key Laboratory of Oncology in South China; Collaborative Innovation Center for Cancer Medicine, Guanzhou 510060, China

##### **Correspondence:** Qian Cui (cuiqian@sysucc.org.cn) – Sun Yat-sen University Cancer Center; State Key Laboratory of Oncology in South China; Collaborative Innovation Center for Cancer Medicine, Guanzhou 510060, China

**Introduction** Nasopharyngeal carcinoma (NPC) is a malignant tumor associated with host genetic predisposition and Epstein-Barr virus (EBV) infection. Disease alerting and early prevention are key points to reduce NPC burden.

**Aims** Our objective is to construct a prediction risk model of NPC based on host genetic and EBV variation, screening those NPC susceptible population in a non-invasive and convenient way ultimately.

**Methods** SNPs and EBV variation were genotyped using the customed Sequenome Mass ARRAY iPlex assay. The association and the interaction were observed under a logistic regression model. An optimal risk prediction model was constructed through forward conditional method to select the variables. Receiver operating characteristic (ROC) curve analysis was used to illustrate the performance of the model.

**Results and discussion** Firstly, we confirmed that the SNP genotyping result has no difference between the buffy coat and the saliva in 116 paired samples. Thus we tested 8 single nucleotide polymorphisms (SNPs; rs2860580, rs2894207, rs28421666, rs9510787, rs1572072, rs6774494, rs1412829 and rs31489) and the EBV subtype (G > A mutation at locus 155391) simultaneously in saliva in a large population-based case-control (1057 NPC cases and 1148 controls) study, which replicated their associations with NPC except for rs1572072. ROC curve analysis revealed the AUC of the NPC risk prediction model inclusive of those 7 SNPs, EBV subtype and their interactions was 0.74 (95 % CI 0.72-0.77) in the training cohort including 705 cases and 776 controls. The AUC was 0.72 (95 % CI 0.66-0.79) in the independent validation cohort of 161 cases and 110 controls. Taken together, this study confirms the strong contributions of genetic and viral components to NPC susceptibility and develops a risk prediction model with these factors using saliva samples. This model can facilitate the screening of high-risk groups in a non-invasive and convenient way and thus promote the prevention and earlier detection of NPC for those people.

## O6 Patterns of care study in Turkish nasopharyngeal cancer patients (NAZOTURK): A Turkish Radiation Oncology Association Head and Neck Cancer Working Group Study

### B Şahin^1^, A Dizman^2^, M Esassolak^3^, A Saran İkizler^4^, HC Yıldırım^5^, M Çaloğlu^6^, B Atalar^1^, F Akman^7^, C Demiroz^8^, BM Atasoy^9^, E Canyilmaz^10^, S Igdem^11^, G Ugurluer^1,12^, T Kütük^13^, M Akmansoy^14^, E Ozyar^1^

#### ^1^Departments of Radiation Oncology of Acibadem University, Istanbul, Turkey; ^2^Ankara Oncology Hospital, Ankara, Turkey; ^3^Ege University, Bornova-İzmir, Turkey; ^4^Acibadem Bursa Hospital, Nilüfer-Bursa, Turkey; ^5^Cerrahpaşa University, Cerrahpaşa-Fatih, Turkey; ^6^Trakya University, Edirne, Turkey; ^7^Eylül University, Alsancak-İzmir, Turkey; ^8^Uludağ University, Nilüfer-Bursa, Turkey; ^9^Marmara University, Kadıköy-Istanbul, Turkey; ^10^Karadeniz Technical University, Trabzon, Turkey; ^11^Bilim University, Beşiktaş-İstanbul, Turkey^12^Acibadem Adana Hospital, Seyhan-Adana, Turkey; ^13^Ankara University, Tandoğan-Ankara, Turkey; ^14^Gazi University, Ankara, Turkey

##### **Correspondence:** E Ozyar (enis.ozyar@acibadem.com.tr) – Departments of Radiation Oncology of Acibadem University, Turkey

**Aim** The objective of this study was to examine patterns of care and patient, tumor and treatment parameters in a sample of patients with nasopharyngeal cancer (NPC) who were diagnosed and treated by 14 Turkish radiotherapy centers in the year of 2014.

**Methods** Data of 122 patients primary NPC from 14 Turkish centers were evaluated for patient's characteristics, staging characteristics, tumor parameters, use of plasma EBV DNA quantification, and trends in treatment.

**Results** Overall data of 122 patients were analyzed. There were 90 male and 32 female patients (M/F ratio: 2.8/1). Age of the patients was ranged between 11-81years old (median: 51 years old). There were 14 patients with age less than 30 years old. Histopathological diagnosis was WHO I (12 patients), WHO II (24 patients), WHO III (81 patients), and unclassified cancer (5 patients). Fourteen (11.4 %) out of 122 patients had cranial nerve involvement at initial diagnosis. PET staging was used in 116 (95 %) and MRI staging was used in 108 (88.5 %) out of 122 patients. Plasma EBV DNA analysis was available in 19 (15.5 %) patients at diagnosis. Six (31.5 %) out of 19 patients were found to be negative at initial diagnosis. Plasma EBV DNA level was ranged between 316-19.747.645 cp/ml (median: 43.683 cp/ml). Patient distribution according to T status was as follows: T1: 28, T2: 46, T3: 21, and T4: 27 patients. N status was as follows: N0: 18, N1: 37, N2: 51, N3a: 9, and N3b: 7 patients. Stage distribution was as follows: Stage I: 4, Stage II: 24, Stage III: 50, Stage IVA: 21, Stage IVB: 15 and Stage IVC: 8 patients. Treatment parameters was as follows; radiotherapy alone: 9, concomitant chemoradiotherapy (CCRT) alone: 64, CCRT + adjuvant chemotherapy: 14, neoadjuvant chemotherapy and radiotherapy: 6, neoadjuvant chemotherapy and CCRT: 28, and chemotherapy alone: 1 patients. Radiotherapy was applied using 2D radiotherapy technique in 12 patients, with 3D conformal radiotherapy in 32 patients, with intensity modulated radiotherapy in 77 patients (63.1 %).

**Conclusions** This study was made as an initial step to achieve collaboration between Turkish radiotherapy centers to execute a multicentre study on NPC. The results have shown that PET-CT and MRI staging is commonly available; however plasma quantification is under-utilized among the centers. While a majority of patients were treated with concomitant CCRT, adjuvant treatment was not preferred as it is in the endemic countries. IMRT is available in two third of the patients. The data obtained in this study can be representative of Turkish NPC patients.

## O7 Long term outcome of intensity modulated radiotherapy in nasopharyngeal carcinoma in National Cancer Centre Singapore

### Kiattisa Sommat, Fu Qiang Wang, Li-Lian Kwok, Terence Tan, Kam Weng Fong, Yoke Lim Soong, Shie Lee Cheah, Joseph Wee

#### National Cancer Centre Singapore, 11 Hospital Drive, Singapore 169610, Singapore

##### **Correspondence:** Kiattisa Sommat (kiattisa.sommat@nccs.com.sg) – National Cancer Centre Singapore, Singapore

**Introduction** Intensity Modulated Radiation Therapy (IMRT) has become standard technique for the treatment of nasopharyngeal cancer (NPC) in the recent years. It has achieved excellent locoregional control with acceptable acute and late toxicities.

**Aim** To report the clinical outcome of NPC patients treated with IMRT in our centre.

**Methods** This is a retrospective analysis of all patients with NPC treated with curative intent in our Department of Radiation Oncology in National Cancer Centre Singapore between 2000 and 2009. Clinical outcome in terms of loco-regional control, disease free survival (DFS) and overall survival (OS) were determined. 937 patients with Stage I-IVB NPC were treated with definitive IMRT during this period. 68.2 % of patients presented with advanced stage; 35.6 % with Stage III and 33.6 % with Stage IVA and IVB.

**Results** The median follow up time is 5.7 years. The 5 year local control rate were as follows: T1: 92.6 %, T2: 88.8 %, T3: 87 %, T4: 77.5 %. The 5 year regional control rate were N0: 97.1 %, N1:93.9 %, N2: 92.7 %, N3a: 89.2 %, N3b: 87.7 %. The 5 year overall OS rate were Stage I: 98.8 %, Stage II: 86.2 %, Stage III: 83.4 %, Stage IVA: 69 %, Stage IVB 71 %. The 5 year DFS were Stage I: 91.6 %, Stage II 76.1 %, Stage III 73.2 %, Stage IVA 59.2 %, Stage IVB 58 %. 265 (28.2 %) patients developed recurrences. Out of these, 91 patients (9.7 %) had locoregional recurrence only, 126 patients (13.5 %) had distant relapse only and 48 patients (5.1 %) had both loco regional and distant relapse.

**Conclusion** IMRT offers excellent long term disease control and survival in patients with NPC. Effective strategy is needed to optimize distant control.

## O8 International phase II randomized study on the addition of docetaxel to the combination of cisplatin and 5-fluorouracil in the induction treatment for nasopharyngeal carcinoma in children and adolescents

### M Casanova^1^, E Özyar^2^, C Patte^3^, D Orbach^4^, A Ferrari^1^, VF Cristine^5^, H Errihani^6^, J Pan^7^, L Zhang^8^, S Liji^9^, K Grzegorzewski^10^, L Gore^11^, A Varan^12^

#### ^1^Fondazione IRCCS Istituto Nazionale dei Tumori, Milan, Italy; ^2^Acibadem University, School of Medicine, Istanbul, Turkey; ^3^Institut Gustave Roussy, Villejuif Cedex, France; ^4^Institut Curie, Paris, France; ^5^Sanofi, Chilly-Mazarin, France; ^6^National Institute of Oncology, University Mohammed V Souissi, Rabat, Morocco; ^7^Cancer Hospital of Fujian Provi, Fujian, China; ^8^State Key Laboratory of Oncology in South China, Collaborative Innovation Center for Cancer Medicine, Sun Yat-Sun University Cancer Center, East Guangzhou, China; ^9^Sanofi; Bridgewater, NJ, USA; ^10^Novartis, East Hanover, NJ, USA; ^11^The Children’s Hospital, Colorado, USA; ^12^Hacettepe University, Institute of Oncology, Ankara, Turkey

##### **Correspondence:** E Özyar (enis.ozyar@acibadem.com.tr) – Acibadem University, School of Medicine, Istanbul, Turkey

**Introduction** Nasopharyngeal carcinoma (NPC) is a rare but aggressive malignancy in children and adolescents.

**Aim** An international, randomized phase II trial was conducted to compare induction chemotherapy with docetaxel plus cisplatin and 5-fluorouracil (TPF) with cisplatin and 5-fluorouracil (PF) in NPC patients under the age of 21.

**Methods** Patients with stage IIB-IV NPC were randomly assigned, in a 2:1 ratio, to receive TPF or PF three-weekly for 3 cycles, followed by chemoradiotherapy. The primary endpoint was the complete response rate achieved with TPF or PF. Docetaxel pharmacokinetics were also evaluated.

**Results and discussion** Seventy-five patients (median 16 years old) were randomized, with 50 assigned to the TPF group and 25 to the PF group. Overall response was assessed after induction treatment: 1 patient in the TPF group and none in the PF group had a complete response. Partial response was achieved in 76.0 % and 80.0 % in the TPF and PF group, respectively. The overall safety profile was consistent with findings in adults. The estimated 3-year overall survival rate was 78.0 % for the PF group and 85.7 % for the TPF group (median follow-up 3.3 years). Mean docetaxel area under the curve was 3.41 μg.h/mL, compared to 3.51 μg.h/mL seen in adult patients.

**Conclusion** This study demonstrated the feasibility of prospective randomized protocols, even for such rare tumors as pediatric NPC. Overall, there were no differences between the 2 treatment arms in terms of efficacy and toxicity. The pharmacokinetics of docetaxel in pediatric patients at 75 mg/m^2^ were similar to those observed in adults.

(This study was supported by Sanofi).

## O9 Prognostic impact of metastatic status in patients with nasopharyngeal carcinoma

### Susanna Hilda Hutajulu^1^, Guntara Khuzairi^2^, Camelia Herdini^3^, Henry Kusumo^4^, Mardiah Suci Hardianti^1^, Kartika Widayati Taroeno-Hariadi^1^, Ibnu Purwanto^1^, Johan Kurnianda^1^

#### ^1^Division of Hematology and Medical Oncology, Department of Internal Medicine, Faculty of Medicine, Universitas Gadjah Mada/Dr Sardjito General Hospital, Yogyakarta, Indonesia; ^2^Study Program of Medicine, Faculty of Medicine, Universitas Gadjah Mada, Yogyakarta, Indonesia; ^3^Department of Otorhinolaryngology Head and Neck Surgery, Faculty of Medicine, Universitas Gadjah Mada/Dr Sardjito General Hospital, Yogyakarta, Indonesia; ^4^Department of Radiology, Faculty of Medicine, Universitas Gadjah Mada/Dr Sardjito General Hospital, Yogyakarta, Indonesia

##### **Correspondence:** Susanna Hilda Hutajulu (susanna.hutajulu@ugm.ac.id) – Division of Hematology and Medical Oncology, Department of Internal Medicine, Faculty of Medicine, Universitas Gadjah Mada/Dr Sardjito General Hospital, Yogyakarta, Indonesia

**Introduction** The M1 stage of TNM classification in nasopharyngeal carcinoma (NPC) does not discriminate patients based on metastatic site and number of metastatic lesions. The role of metastatic status in predicting survival has not been analyzed in the local setting.

**Aim** This study aimed to determine the prognostic impact of metastatic status of NPC patients.

**Methods** Data of 81 metastatic NPC patients diagnosed between January 2007 and December 2011 were retrospectively reviewed. Diagnosis of metastasis was based on imaging results. Patients were grouped according to various prognostic variables, including age (<50 versus ≥ 50 years), sex, histology grading, T and N classification, the time order of metastasis (synchronous versus metachronous), and number of metastatic sites (single versus multiple). Rates of overall survival (OS) were calculated using the Kaplan-Meier method. The log rank test was performed to analyze the difference between survival curves. Cox regression was used to calculate the hazard ratio (HR) and 95 % confidence interval (CI).

**Results and discussion** The median OS was 24.4 months (ranged from 12.4-36.4 months). The 1 year-, 2 year-, and 3 year-survival rate were 64.4 %, 49.5 %, and 39.0 %, respectively. Involved single metastatic sites included the bone (39, 48.1 %), lung (18, 22 %), liver (11, 13.6 %) and brain (1, 1.2 %), whereas the involvement of multiple organs occurred in 12 (14.8 %) patients. In univariate analysis, patients with lung-only metastasis had favourable survival compared with other single- (HR = 0.698, 95 % CI 0.236-2.065) and multiple-distant metastases (HR = 0.195; 95 % CI0.653-0.058). T4 and N1-N3 were insignificant negative predictors for survival (HR = 1.937, 95 % CI 0.887-4.229 and HR = 3.712, 95 % CI 0.502-27.472, respectively). Interestingly, cases with metachronous lesion had worse survival than those with synchronous disease (HR = 2.312, 95 % CI 1.090-4.900). However, when tested in multivariate analysis, metastatic site was the only significant independent prognostic factor for patients’ survival (95 % CI 0.912-0.449), while metastatic time lost its prognostic influence (95 % CI 0.224-1.018).

**Conclusion** In the local patient cohort, metastatic site had a prognostic impact on patients’ survival.

## O10 Development of small molecule inhibitors of latent Epstein-Barr virus infection for the treatment of nasopharyngeal carcinoma

### Troy E. Messick^1^; Kimberly Malecka^1^; Lois Tolvinski^1^; Samantha Soldan^1^; Julianna Deakyne^1^; Hui Song^1^; Antonio van den Heuvel^2^; Baiwei Gu^2^; Joel Cassel^2^; Mark McDonnell^2,3^; Garry R Smith^3^; Venkata Velvadapu^3^; Haiyan Bian^3^; Yan Zhang^3^; Marianne Carlsen^3^; Shuai Chen^3^; Alastair Donald^4^; Christian Lemmen^4^; Allen B Reitz^3^; Paul M Lieberman^1^

#### ^1^The Wistar Institute, 3601 Spruce Street, Philadelphia, PA 19104, USA; ^2^Vironika, LLC, 3805 Old Easton Road, Doylestown, PA 18902, USA; ^3^Fox Chase Chemical Diversity Center, 3805 Old Easton Road, Doylestown, PA 18902, USA; ^4^Bio Solve IT GmbH, An der Ziegelei 79, 53757 Sankt Augustin, Germany

##### **Correspondence:** Troy E. Messick (tmessick@Wistar.org) – The Wistar Institute, 3601 Spruce Street, Philadelphia, PA 19104, USA

**Introduction** EBV is a DNA tumor virus etiologically associated with 1-2 % of all human cancers including Burkitt's lymphoma (BL), Hodgkin's lymphoma and nasopharyngeal carcinoma (NPC). Currently, no pharmaceutical-based therapies exist that selectively target EBV latent infection. EBV latent infection depends on the continuous expression of Epstein-Barr Nuclear Antigen 1 (EBNA1), a multifunctional dimeric protein critical for viral replication, genome maintenance, and viral gene expression.

**Aim** The aim of this program is to advance the development of a New Chemical Entity (NCE) for latent infection of Epstein-Barr Virus (EBV) to treat NPC.

**Methods** We have used structure-based drug design and medicinal chemistry methods to develop a small molecule lead series that selectively inhibits the DNA-binding activity of EBNA1.

**Results and discussion** The lead series inhibits the EBNA1 function with nanomolar potency in biochemical assays and low micro molecular activity in several cell-based assays, including ChIP assays. We demonstrate that these inhibitors provide protection in xenograft models of EBV-driven tumor growth of NPC cell lines. Furthermore, EBER-ISH experiments confirm *in vivo* target engagement by the elimination of EBV in treated tumor tissue. The inhibitors are selective, showing no activity against KSHV LANA in the biochemical assay, no cell-killing activity of EBV-negative cells, and no tumor growth inhibition in an EBV-negative xenograft experiments. The lead series meet or exceed industry-accepted criteria for drug suitability, safety and toxicology including physicochemical properties, metabolic stability, selectivity in broad-based screens and bioavailability, and the absence of *in vivo* liabilities, including genotoxicity and 14-day toxicity studies. These data establish proof-of-concept for targeting EBNA1—a protein previously thought to be undruggable.

## O11 Therapeutic targeting of cancer stem-like cells using a Wnt modulator, ICG-001, enhances the treatment outcome of EBV-positive nasopharyngeal carcinoma

### King Chi Chan^1^, Lai Sheung Chan^1^, Kwok Wai Lo^2^, Timothy Tak Chun Yip^3,4^, Roger Kai Cheong Ngan^3,4^, Michael Kahn^5^, Maria Li Lung^4,6^, Nai Ki Mak^1,4^

#### ^1^Department of Biology, Hong Kong Baptist University, Hong Kong, P.R. China; ^2^Department of Anatomical and Cellular Pathology, State Key Laboratory in Oncology in South China, The Chinese University of Hong Kong, Hong Kong, P.R. China; ^3^Department of Clinical Oncology, Queen Elizabeth Hospital Hong Kong, P.R. China; ^4^Center for Nasopharyngeal Carcinoma Research, University of Hong Kong, Hong Kong, P.R. China^5^Center for Molecular Pathways and Drug Discovery, University of Southern California, Los Angeles, CS, USA; ^6^Department of Clinical Oncology, University of Hong Kong, Hong Kong, P.R. China

##### **Correspondence:** King Chi Chan (biocandy@hkbu.edu.hk) – Department of Biology, Hong Kong Baptist University, Hong Kong, P.R. China

**Introduction** Cancer stem-like cells (CSCs) are sub-population of treatment resistant cells and have the tumor initiating ability. The presence of CSCs is suggested to be associated with tumor relapse and metastasis after conventional treatments. The Wnt signaling pathway involved in CSC maintenance is known to be aberrantly activated in most of the NPC patients. ICG-001 is a Wnt modulator that can specifically block the interaction between coactivator CBP and β-catenin to inhibit the transcription of genes associated with the maintenance of stemness properties.

**Aim** We hypothesized that ICG-001 can reduce NPC cells with stem-like phenotypes and enhances the efficacy of cisplatin on NPC.

**Methods** We employed the 3-D tumor sphere formation assay as a functional assay to enrich the CSC-like cellsto study the effect of ICG-001 on the CSC-like population. Also, we performed a combination study of ICG-001 with the conventional drug cisplatin to evaluate the *in vitro* and *in vivo* efficacy of the treatment on NPC.

**Results and discussion** Using the EBV-positive C666 cell model, ICG-001 was found to significantly reduce the growth of tumor spheres and reduce the spheroid cells with SOX2^hi^/CD44^hi^ CSC-like phenotype. The reduced SOX2 expression was found to be associated with restored expression of the tumor suppressive microRNA-145 (miR-145). Ectopic expression of miR-145 effectively repressed SOX2 expression and inhibited tumor sphere formation. Furthermore, the combination of ICG-001 with cisplatin demonstrated a better tumor suppressive effect than the cisplatin treatment alone on the EBV-positive NPC xenograft models. These results suggest that therapeutic targeting of the Wnt signaling pathway by ICG-001 may reduce the CSC-like population in NPC and enhance the tumor suppressive effect of NPC to cisplatin. Future clinical investigations of ICG-001 (PRI-724, the second generation of ICG-001 for clinical study) combined with cisplatin may benefit NPC patients from a better treatment outcome.

## O12 Role of micro-RNA in NPC biology

### Fei-Fei Liu (fei-fei.liu@rmp.uhn.on.ca)

#### Department of Radiation Oncology, Princess Margaret Cancer Center, University of Toronto, Toronto, Canada

**Introduction** One of the major clinic-translational challenges in nasopharyngeal carcinoma (NPC) is the pre-identification of patients who will develop distant metastasis in order to develop a personalized treatment approach.

**Aim** Using a training: validation design, we wished to identify a micro-RNA (miRNA) signature which can identify risk of developing distant metastasis in NPC patients.

**Methods** Global miRNA was conducted on 246 primary human NPC formalin-fixed paraffin-embedded (FFPE) biopsy samples using the Nanostring nCounter Human miRNA Platform, which measured the expression of 732 miRNAs, including 654 human and 78 viral, including EBV miRNAs.

**Results and discussion** From the 125 samples in the training set, the expression levels of 4-miRNAs were identified to be significant prognosticators for distant metastasis: miR-154, miR-449b, miR-140, and miR-34c, with a HR of 8.25 (p < 0.001). This was validated in the second independent NPC cohort of 121 samples, with a HR of 3.2 (p = 0.010), and determined to be independently prognostic, in addition to clinical nodal status (Bruce *et al; Oncotarget* 6:4537; 2015). Pathway analysis indicated that cell cycle regulation appeared to be targeted by these 4-miRNAs, which was corroborated to be an important pathway based on results of a recent whole exome sequencing study (Lin *et al; Nat Genet* 46:866, 2014). Preliminary data generated using NP69 (normal nasopharyngeal epithelial) cell lines indicated that transfection using anti-miR-34c plus pre-miR-449b suppressed cell proliferation, and potential mRNA targets including CDKN1A and p53 were identified, as well as bcl-2, DNMT1, NOTCH1, HMGA2, and MMP13.

**Conclusion** Further interrogation of these 4-miRNAs could provide important biological insights that could facilitate the discovery and development of novel molecularly-targeted therapies to improve outcome for future NPC patients by preventing distant metastasis.

## O13 Expansion of EBNA1- and LMP2-specific effector T lymphocytes from patients with nasopharyngeal carcinoma without enhancement of regulatory T cells

### Wafa Khaali^1,2,3,4^, Juliette Thariat^1,5^, Laurence Fantin^1,2^, Flavia Spirito^1,2^, Meriem Khyatti^3^, El Khalil Ben Driss^4^, Sylvain Olivero^1,2^, Janet Maryanski^1,2^, Alain Doglio^1,2^

#### ^1^Centre Hospitalier Universitaire de Nice, Unité de Thérapie Cellulaire et Génique, Nice, France*;*^2^Université Nice-Sophia Antipolis, UFR Médecine, MICORALIS, Nice, France; ^3^Institut Pasteur du Maroc, Laboratoire Onco-virologie, Casablanca, Morocco; ^4^Université Abdelmalek Essaâdi, Faculté des sciences, Laboratoire Ecologie, Biodiversité et Environnement, Tétouan, Morocco; ^5^Département de Radiothérapie, Centre Antoine-Lacassagne, Nice, France

##### **Correspondence:** Wafa Khaali (wafa.khaali@gmail.com) – Centre Hospitalier Universitaire de Nice, Unité de Thérapie Cellulaire et Génique, Nice, France

**Introduction** Nasopharyngeal carcinomas (NPC) are endemic in Asia and Mediterranean areas, where they are associated with Epstein Barr Virus (EBV) infection. They are diagnosed at an advanced stage in 75-90 % of cases owing to their deep anatomic location. Despite better response than in other head and neck cancers, the five-year survival is about 75 %. However, the outcomes for patients with metastatic or locally recurrent EBV-positive NPC remain poor.

**Aim** To develop more effective therapies for NPC, adoptive lymphocyte therapy with EBV-specific T-cells has been proposed as a very promising immunotherapeutic approach to treat EBV-associated diseases.

**Methods** In this study, we performed a novel procedure to rapidly generate multivirus-specific cytotoxic lymphocytes for treatment of viral infection proposed recently by Gerdemann et al. 2012. A specific preparation of polyclonal specific antiviral T-CD4 and T-CD8 population can be achieved by a single round of stimulation with viral peptides and growth in the presence of prosurvival cytokines.

**Results and discussion** In the present study we show that this approach is suitable to rapidly generate batches of specific EBV latency II T-cell from NPC, which importantly are devoid of regulatory T-cell.

## O14 The experience of patients’ life after amifostine radiotherapy treatment (ART) for nasopharyngeal carcinoma (NPC)

### Mengxue Xia^1^, Yunfei Xia^2^, Hui Chang^2^, Rachel Shaw (r.l.shaw@aston.ac.uk)^1^

#### ^1^Aston University, Birmingham, UK; ^2^Cancer Hospital of Sun Yat-Sen University, Guangzhou, China

##### **Correspondence:** Mengxue Xia (iisabel.mx@gmail.com) – Aston University, Birmingham, UK

**Introduction** Little is known about patients’ experiences of Amifostine radiotherapy treatment (ART) for nasopharyngeal carcinoma (NPC) or how it impacts on their quality of life (QoL).

**Aims** The study examined patients’ perspectives on ART, in order to explore their understanding and experience on medium to long-term effects QoL. Taking a patient-centered approach enabled an exploration of perceived limitations of the treatment, and the opportunities for improvement in relation to patients’ psychological wellbeing.

**Methods** Six semi-structured interviews were conducted using questions inspired by EORTC QLQ-C30 questionnaire and QLQ-H&N35 module for examining QoL in head and neck cancer patients. The participants selected had undergone ART at the Radiotherapy Department of Cancer Hospital of Sun Yat-sen University and were cancer-free, i.e. successfully completed treatment at least 12 months previously with no signs of recurrence. Thematic analysis was used to analyse the interview transcripts.

**Results and discussion** The study found that ART had minimal negative impact upon patients’ QoL in long-term. NPC survivors suffered from mild long-term side effects (dry mouth), with only short-term severe side effects (headaches, ragged oral cavity, and tiredness). Since treatment, patients exhibited a sensitive self-awareness that allowed them to maintain a positive attitude towards the treatment. They were highly attached to the senior consultant and sought opportunities to meet him, which helped patients to maintain a positive attitude. However, due to lack of understanding on their treatment, several patients expressed feelings of anxiety that triggered them to seek information online, which only resulted in further anxiety. Participants also reported strong fears of relapse, and doubted the results provided by the hospital. Despite coming into contact with several other healthcare professionals, patients expressed a need to seek reassurance from the senior doctor. Thus, this study suggests a need for more comprehensive management of information shared with patients regarding their treatment, and structured psychological support at all stages of the treatment. Furthermore, better use of the wider clinical team could be used following work with patients to raise awareness of their roles.

## O15 Analysis of mitochondrial DNA mutation in latent membrane protein-1 positive nasopharyngeal carcinoma

### Pudji Rahaju (pudjitht@gmail.com; pudji_tht@yahoo.co.id)

#### Faculty of Medicine Brawijaya University, Malang, Indonesia

**Introduction** Nasopharyngeal carcinoma (NPC) is the most malignant disease in the head and neck areas. Epstein-Barr Virus (EBV) plays an important role in NPC carcinogenesis. Mitochondria is important in intrinsic apoptotic signaling and ATP energy forming through oxidation-phosphorylation.

**Aims** We have explored the influences of mitochondrial DNA (mtDNA) mutation particularly cytochrome-oxidase I, II, III, ATP-synthase 6 and 8 genes from nasopharyngeal tissue biopsy of NPC patient (WHO III, LMP-1 positive) in apoptotic signaling and ATP energy forming.

**Methods** We used nasopharyngeal tissue biopsy and determined mutation of mitochondrial DNA CO I, II, III, ATPase 6 and 8 by electrophorese, PCR and sequencing. We checked the distribution of protein cytosol cytochrome-c, caspase 9, 3 and ATP synthase by immunohistochemistry. We analyzed the data using Chi-square test and ANOVA.

**Result and discussion** By using Chi-square it was found that there was a significant association between the type of mtDNA mutation and the distribution of those proteins. By ANOVA analysis it was found that there was significant association between the type of genes and the number of mutation between CO III gene as compared to CO I and CO II genes, ATPase 8 with ATPase 6, substitution with deletion and insertion. It was concluded that all EBV LMP-1 positive-NPC patients who experienced mtDNA mutation of coding region and types of mutation in those genes had a strong association toward the distribution of protein cytosol that induce apoptosis and ATP energy forming.

## O16 Factors influencing treatment adherence of nasopharyngeal cancer and the clinical outcomes: a hospital-based study

### Mardiah Suci Hardianti^1^, Sindhu Wisesa^2^, Kartika Widayati Taroeno-Harijadi^1^, Ibnu Purwanto^1^, Bambang Hariwiyanto^3^, Wigati Dhamiyati^4^, Johan Kurnianda^1^

#### ^1^Division of Hematology and Medical Oncology, Department of Internal Medicine, Faculty of Medicine Universitas Gadjah Mada, Yogyakarta, Indonesia; ^2^Department of Anatomy, Faculty of Medicine, Jenderal Soedirman University, Purwokerto, Indonesia; ^3^Department of Otorhinolaryngology Head and Neck Surgery, Faculty of Medicine, Universitas Gadjah Mada, Yogyakarta, Indonesia; ^4^Division of Radiotherapy, Department of Radiology, Faculty of Medicine, Universitas Gadjah Mada, Yogyakarta, Indonesia

##### **Correspondence:** Mardiah Suci Hardianti (diahbudiyanto@yahoo.com) – Division of Hematology and Medical Oncology, Department of Internal Medicine, Faculty of Medicine Universitas Gadjah Mada, Yogyakarta, Indonesia

**Introduction** Adherence to treatment regimens offers both a better survival rate and lower recurrence in nasopharyngeal cancer (NPC) patients. Various factors influencing the adherence to long term therapy include socio economic and medical factors. Understanding those factors is essential to improve the patients’ outcome.

**Aims** This study aims to define factors influencing adherence of NPC patients to their treatment regimens and the correlations between those factors and clinical outcomes.

**Methods** A retrospective cohort study was conducted in Dr. Sardjito Hospital based on medical record from 2007-2011. Factors examined were socio-demography, baseline characteristics, and survival. Adherence was defined as completion of the entire course of therapy and clinical follow-up. Data were described and analyzed with Kruskall-Wallis analysis. Kaplan-Maier survival analysis was done to observe its clinical outcome.

**Result and discussion** From 274 NPC cases, we found 184 (67.2 %) patients were adherent. Most of non-adherent patients (32.8 %) were due to lost to follow up (73.3 %), financial problems (9.9 %), adverse event (7.8 %), patient refusal (4.4 %), death (4.4 %), and empty stock of drugs (1.1 %). Patient adherence showed significant correlations with baseline staging (p = 0.0001), with stage III-IVA showed better adherence compared with those in stage IVB-IVC. Patients with nasal blockage (p = 0.050), epistaxis (p = 0.320), ringing ear (p = 0.570) had been more adherent compared with other complaints. The overall survival (OS) and clinical response were better in adherent than non-adherent patients (p = 0.0001) with median OS of 1.5 and 0.53 years, respectively. Moreover, adherent patients had more significant result in one, two, and three-year OS (p = 0.0001; p = 0.0001; p = 0.040). However, it declined in the following four and five year OS (p = 0.993; p = 0.174). The adherence showed poor correlations with sex (p = 0.534), first visit age (p = 0.916), education status (p = 0.947), occupation (p = 0.752), covering insurance (p = 0.825), neck mass complain (p = 0.347), faster diagnosis (p = 0.592), histopathology type (p = 0.278), and free-disease survival (p = 0.341). Further attention should be given to the factors influencing the adherence to treatment to finally improve patients outcome.

## O17 Chromosomal breaks mediated by bile acid-induced apoptosis in nasopharyngeal epithelial cells: in relation to matrix association region/scaffold attachment region

### Sang-Nee Tan, Sai-Peng Sim

#### Faculty of Medicine and Health Sciences, Universiti Malaysia Sarawak, Sarawak, Malaysia

##### **Correspondence:** Sang-Nee Tan (tansangnee@gmail.com) – Faculty of Medicine and Health Sciences, Universiti Malaysia Sarawak, Sarawak, Malaysia

**Introduction** Chronic rhinosinusitis (CRS) has been recognised as a risk factor for nasopharyngeal carcinoma (NPC). CRS can be triggered by gastroesophageal reflux (GER) that may reach the nasopharynx. Bile acid (BA), the main component of refluxate, is a potential carcinogen. BA-induced apoptosis has been implicated in various malignancies. Chromosomal breakage is an early event in both apoptosis and chromosomal rearrangement. Matrix Attachment Region/Scaffold Attachment Region (MAR/SAR) appears to be a preferential site of chromosomal breakage. We hypothesised that BA-induced apoptosis may cause chromosomal breaks at MAR/SAR leading to NPC chromosomal rearrangements.

**Aims** To identify BA-induced chromosomal breaks within *AF9* (9p22) SAR and non-SAR regions.

**Methods** MAR/SAR sites in the *AF9* gene were predicted by MARSCAN. NP69 cells were treated with BA at neutral and acidic pH. Flow cytometric analyses of phosphatidylserine (PS) externalization and mitochondrial membrane potential (MMP) disruption were performed. Inverse-PCR (IPCR) was employed to detect cleavages in SAR and non-SAR regions. IPCR bands were sequenced.

**Result and discussion** Treatment of NP69 cells with BA at neutral and acidic pH resulted in increased apoptosis and increased cleavage frequencies of the SAR region. No significant difference was detected in non-SAR cleavage frequency between untreated cells and cells treated with BA at neutral or acidic pH. A few breakpoints detected in the SAR region were mapped within the *AF9* region that was previously reported to be involved in the formation of *MLL* (Mixed Lineage Leukaemia)-*AF9* fusion gene in acute lymphoblastic leukaemia (ALL) patient. Our findings suggested that BA-induced apoptosis could be one of the mechanisms underlying NPC chromosomal rearrangements. MAR/SAR may play an important role in chromosomal breaks mediated by BA-induced apoptosis.

## O18 Expression of *p53* (wild type) on nasopharyngeal carcinoma stem cell that resistant to radiotherapy

### Muhtarum Yusuf^1^, Ahmad C Romdhoni^1^, Widodo Ario K^1^, Fedik Abdul Rantam^2^

#### ^1^Oncology Division Departement of Otolaryngology Head and Neck sugery Faculty of Medicine Airlangga University/Dr. Soetomo Hospital Surabaya, Indonesia; ^2^Institute of Tropical Disease Center, Universitas Airlangga Surabaya, Indonesia

##### **Correspondence:** Muhtarum Yusuf (muhtarumyusuf@yahoo.co.id) – Oncology Division Departement of Otolaryngology Head and Neck sugery Faculty of Medicine Airlangga University/Dr. Soetomo Hospital Surabaya, Indonesia

**Introduction** Recurrences remain frequent in nasopharyngeal carcinoma (NPC) patients, despite having received complete therapy. Recent studies have proven that recurrences were caused by NPC cancer stem cells that were resistant to radiotherapy. The mechanism of resistance in cancer stem cells to radiotherapy was assumed to be due to the blocking of apoptosis and/or proliferation induction. The blocking of apoptosis was caused by the decreased of *p53* (wild type) expression.

**Aim** To describe the mechanism of NPC stem cells resistance to radiotherapy based on p53 (wild type) profiles.

**Methods** We used a experimental study with pre- and post-test control group design. The cultured NPC stem cells were divided into two groups, each with 16 samples. The treatment group had 1.5Gy dose of radiotherapy exposure with Linac device, then incubated for 24 hours. Both groups were analyzed for p53 (wild type) before and after treatment. The analysis were done by flow cytometry.

**Result** The *p53* (wild type) expression between the treatment and control group showed no significant difference (p = 0.576).

**Conclusion** No changes of p53 (wild type) on NPC stem cell that resistant to radiotherapy.

## O19 Mathematical model of nasopharyngeal carcinoma in cellular level

### Sugiyanto^1^, Lina Aryati^1^, Fajar Adi-Kusumo^1^, Mardiah Suci Hardianti^2^

#### ^1^Department of Mathematics, Universitas Gadjah Mada, Yogyakarta, Indonesia; ^2^Faculty of Medicine, Universitas Gadjah Mada, Yogyakarta, Indonesia

##### **Correspondence:** Sugiyanto (sugimath@yahoo.co.id) – Department of Mathematics, Universitas Gadjah Mada, Yogyakarta, Indonesia

**Introduction** Nasopharyngeal carcinoma (NPC) is a cancer that occurs due to tumor malignancies in nasopharyngeal epithelial cells. This cancer is related to Epstein-Barr Virus (EBV) infection, as almost all NPC tumor cells contain EBV, and most people with NPC showed evidence of infection by this virus in their blood.

**Aims** The aim of the research is to determine the length of the process of development from normal cells into invansive carcinoma cells. This research also can describe the comparison of the amount of normal cells and invansive carcinoma cells at a certain time.

**Methods** Mathematical modeling on the development of normal cells to invasive carcinoma cells. The processes in celluler level were divided into six sub-populations consisting of normal, lesion, low dysplastic, infection, high dysplastic, invansive carcinoma cells and one lytic EBV sub-population. All sub-populations are modeled in the system of differential equations.

**Result and discussion** Within a mathematical model, we obtained three conditions of cells where the virus exists, i.e. the lesions which occurred from non-infected cells, the cells which are infected but did not develop into invasive carcinoma, and the infected cells with invasive carcinoma.

## O20 Differential expression of microRNA-21 on nasopharyngeal carcinoma plasma patient

### SY Bintoro^1^*,* R Oktriani^1^, C Herawati^2^, A Surono^3^, Sofia M Haryana^1^

#### ^1^Department of Molecular Biology, Faculty of Medicine, Universitas Gadjah Mada, Yogyakarta, Indonesia; ^2^Depatment of Otorhinolaryngology Head and Neck Surgery, Dharmais Cancer Hospital, Jakarta, Indonesia; ^3^Department of Otorhinolaryngology Head and Neck Surgery, Faculty of Medicine, Universitas Gadjah Mada/Dr Sardjito Hospital, Yogyakarta, Indonesia

##### **Correspondence:** SY Bintoro (yudhadokmol@gmail.com) – Department of Molecular Biology, Faculty of Medicine, Universitas Gadjah Mada, Yogyakarta, Indonesia

**Introduction.** Nasopharyngeal carcinoma (NPC) is a common malignancy of the head and neck in South-East Asia, including Indonesia. MicroRNA-21 (miR-21) has been considered as the most active miRNA and play important role in several cancers, including NPC.

**Aim.** In this research, we analyzed the expression of miR-21 on NPC patients and compared it to healthy people. We also determined its correlation to the stage of NPC.

**Methods.** A total of 63 samples from 53 NPC patients and 10 healthy controls were used in this study. In the patient group, there were seven individuals with early stage and 46 with advanced stage. Total RNA were isolated from plasma, cDNA was synthesized, and quantified by means of qPCR.

**Result and discussion.** Our results showed that miR-21 expressions in patients’ samples were significantly different from those of healthy controls (p = 0.0013). The Livak’s model showed that the expression of miR-21 on NPC plasma was elevated to 20 times than healthy control. The expression of miR-21 on patients with advanced stage was up to 10 times than those with early stage.

## O21 Therapeutic targeting of an oncogenic fibroblast growth factor-FGF19, which promotes proliferation and induces EMT of carcinoma cells through activating ERK and AKT signaling

### L. Zhong, L. Li, BB Ma, A. T. Chan, Q. Tao

#### Cancer Epigenetics Laboratory, Department of Clinical Oncology, State Key Laboratory of Oncology in South China, Sir YK Pao Center for Cancer, Li Ka Shing Institute of Health Sciences, The Chinese University of Hong Kong, Hong Kong

##### **Correspondence:** Q. Tao (qtao@clo.cuhk.edu.hk) – Cancer Epigenetics Laboratory, Department of Clinical Oncology, State Key Laboratory of Oncology in South China, Sir YK Pao Center for Cancer, Li Ka Shing Institute of Health Sciences, The Chinese University of Hong Kong, Sha Tin, Hong Kong

The endocrine fibroblast growth factor 19 (FGF19) subfamily contains three members - FGF19, FGF21 and FGF23, critically implicated in the pathogenesis of multiple malignancies. Here we report that, although with limited expression in few normal adult tissue types, FGF19 is overexpressed in multiple carcinomas especially nasopharyngeal carcinoma (NPC) and digestive tract cancer tissues. Overexpression of FGF19 promoted tumor cell proliferation and colony formation, and also enhanced tumor cell migration and invasion. Knockdown of endogenous FGF19 suppressed the viability, clonogenicity, migration and invasion abilities of tumor cells. FGF19 activated ERK, AKT and β-catenin signaling through upregulating the phosphorylation of AKT, β-catenin and ERK1/2. We also found that knock-down of FGF19 inhibited tumor cell epithelial-mesenchymal transition (EMT), accompanied by increased expression of epithelial marker E-cadherin and decreased expression of mesenchymal markers N-cadherin, SLUG and fibronectin. FGF19 regulated F-actin rearrangement through promoting RhoA phosphorylation, further modulating the EMT process. Moreover, FGF19 could induce tumor cell resistance to doxorubicin through ERK signaling pathway. Application of an anti-FGF19 antibody successfully suppressed tumor cell migration and invasion. Thus, FGF19 exerts oncogenic functions in NPC and other tumor cells, and could serve as a therapeutic target.

## O22 Resist nasopharyngeal carcinoma (NPC): next generation T cells for the adoptive immunotherapy of NPC

### M. Kalra, M. Ngo, S. Perna, A. Leen, N. Lapteva, C. M. Rooney, S. Gottschalk

#### Center for Cell and Gene Therapy, Texas Children's Hospital, Houston Methodist Hospital, Baylor College of Medicine, Houston, Texas-77030, USA

##### **Correspondence:** M. Kalra (mxkalra@txch.org) – Center for Cell and Gene Therapy, Texas Children's Hospital, Houston Methodist Hospital, Baylor College of Medicine, Houston, Texas-77030, USA

**Introduction** We have demonstrated that administration of autologous EBV-specific T cells (EBV-T cells) to patients with NPC is feasible, safe and associated with anti tumor responses especially for loco-regional disease. However, the anti-NPC activity of EBV-T cell lines generated by our standard method is limited by several factors, including: i) low frequency of T-cells targeting EBV antigens selectively expressed in NPC (LMP1, LMP2, EBNA1 and BARF1), and ii) sensitivity of infused T cells to the immune suppressive tumor microenvironment.

**Aim** The goal of this study was to generate a clinical grade NPC-specific T-cell product that recognizes at least 2 EBV antigens expressed in NPC and that is resistant to TGFβ, a common immune evasion strategy used by tumors including NPC.

**Methods** We used good manufacturing practice (GMP) grade pepmixes, cytokines, and artificial antigen presenting cells to generate NPC-specific T-cell lines. These T cells were genetically modified to express a dominant negative TGFβ-receptor (TGFβ-DNR) to render them resistant to TGFβ.

**Results and discussion** We successfully generated NPC-specific T-cells from 7 NPC patients. As compared to our standard method, manufacturing time was reduced from 3 months to less than 4 weeks. As judged by IFNγ elispot assays, generated T-cell lines recognized at least 2 NPC-associated EBV antigens, and had 1-2 logs higher frequency of T cells specific for LMP1, LMP2, EBNA1 and/or BARF1 in comparison to T cell lines generated by our standard method. Transduction of NPC-specific T cells with a GMP-grade retro virus encoding TGFβ-DNR resulted in transgene expression in 50-80 % of T cells detected by FACS analysis. A clinical study (NCT02065362) to evaluate the safety and antitumor activity of our ‘next generation’ NPC-specific T cells opened for patient accrual in January 2015. TGF-β resistant, NPC-specific T cells may present a promising alternative to currently available therapies for NPC.

## O23 The correlation of heat shock protein 70 expressions and staging of nasopharyngeal carcinoma

### Elida Mustikaningtyas^1^, Sri Herawati^1^, Achmad C. Romdhoni^1,2^

#### ^1^Department of Otorhinolaryngology Head and Neck Surgery, Faculty of Medicine, Universitas Airlangga, Surabaya, Indonesia; ^2^Stem Cell Laboratory, Institute of Tropical Disease, Universitas Airlangga, Surabaya, Indonesia

##### **Correspondence:** Achmad C. Romdhoni (romdhoni_ent@yahoo.com) – Department of Otorhinolaryngology Head and Neck Surgery, Faculty of Medicine, Universitas Airlangga, Surabaya, Indonesia

**Introduction** Recent research demonstrates that intracellular Hsp70 has a crucial role in the growth, invasion, and metastasis of NPC.

**Aims** This study was conducted to analyze intracellular Hsp70 protein expression of NPC samples and see the correlation between the expression and the NPC stage.

**Methods** The study was cross sectionally designed. Formalin-fixed paraffin-embedded biopsy specimens were obtained from 20 cases. All patients were histopathologically confirmed as NPC and clinically determined as stage I, II, III, or IV. The expression of intracellular Hsp70 was detected by immunohistochemistry using anti human Hsp70 antibodi (Santa Cruz Biotechnology, California, USA). Staining assessment was performed using Remmele method. Statistical analysis was done using the Spearman’s test. Statistical significance was defined as p < 0.05.

**Results and discussion** The negative staining of intracellular Hsp70 expression was found in 15 % of the samples which consisted of 1 patient of stage I, II and III, and no patient of stage IV. Mild positive was found in 25 % of the samples which consisted of no patient of stage I and III, 2 patients of stage II, and 3 patients of stage IV. Moderate positive was found in 25 % of the samples which consisted of no patient of stage I and II, 1 patient of stage III, and 9 patients of stage IV. Strong positive was found in 10 % of the samples which consisted of no patient of stage I, II, III, and 2 patients of stage IV. Spearman’s test showed a correlation coefficient of 0.671 (p = 0.001), suggesting a correlation between increasing of the intracellular Hsp70 expression and NPC clinical stage.

## O24 Epstein-Barr virus serological profiles of nasopharyngeal carcinoma - A tribute to Werner Henle

### Mingfang Ji, YaruiXu, Weimin Cheng, ShengxiangGe, Fugui Li, Ng MH

#### Cancer Research Institute of Zhongshan City, Zhongshan People’s Hospital, Zhongshan City, Guangdong Province, PRC

##### **Correspondence:** Mingfang Ji (jmftbh@sina.com) – Cancer Research Institute of Zhongshan City, Zhongshan People’s Hospital, Zhongshan City, Guangdong Province, PRC

**Introduction** In 1976, Werner Henle and colleagues reported that serum levels of Epstein-Barr Virus (EBV) antibody of immunoglobulin subtype A (IgA) were generally elevated among nasopharyngeal carcinoma (NPC) patients. It was believed that this “outstanding” serological feature might be attributed at least partly to the life cycle of the tumor-associated virus.

**Aim** To test this hypothesis and to explore the scope for application of this feature in management of NPC.

**Methods** We developed seven EBV specific ELISA’s using purified recombinant proteins of the following EBV antigens: EBNA1, EBNA2 associating with EBV latency; the non-structural antigens, zta, TK, EA-R EA-D comprising the early antigen complex (EA); and the structural antigen, VCAp18, associating with replication cycle of the virus.

**Results and discussion** We determined levels of the corresponding IgA antibodies of 347 donors and 182 newly NPC patients. All the patients’ sera were obtained before treatment, 64 were occult cases identified through NPC screening program and apparently not aware of by the patients and 118 were symptomatic cases presenting at outpatients’ clinics. We showed that the levels of all the antibodies were significantly higher for NPC patients than the donors, except EBNA2 antibody. The levels of EBNA1 antibody were similar for the occult cases and the symptomatic cases and remained relatively constant as the disease progressed. In contrast, the levels of the EA antibodies were higher for the symptomatic cases than the occult cases. The levels also increased as disease advanced, and with tumor bulk and involvement of loco-regional lymph nodes. But, with the possible exception of TK, the levels of the EA antibodies did not appear to also increase with metastasis. The levels of p18 antibody of the NPC patients were higher than the donors, but did not consistently change with disease progression.

**Conclusion** These findings suggested that the outstanding serological feature originally observed by Henle and colleagues is attributed to the EBV associating with loco-regional tumors. The serological profiles are compatible with a virus life cycle characterized by latency II attended by occasional reactivation of the latent virus which usually aborts before production of virus.

## O25 Targeting the apoptosis pathway using combination TLR3 agonist with anti-survivin molecule (YM-155) in nasopharyngeal carcinoma

### Louise SY Tan, Benjamin Wong, CM Lim (chwee_ming_lim@nuhs.edu.sg)

#### National University Health System, Singapore, Singapore

##### **Correspondence:** Louise SY Tan – National University Health System, Singapore

**Introduction** Distant relapse is a major cause of mortality among patients with nasopharyngeal cancer (NPC). This preclinical study explores the feasibility of targeting the apoptotic pathway in NPC.

**Aims** We aim to investigate the enhancement of NPC cells apoptosis following combination therapy with poly I:C and YM-155, as well as determined the degree of cisplatin induced cytotoxicity of pre-treated NPC cells with YM-155 and poly I:C.

**Methods** TLR3 and survivin expression were investigated on NPC cell lines (HONE1 and C-666) before and after treatment with poly I:C and YM-155 by using flow cytometry. Apoptotic cell death was evaluated using Annexin V and propidium iodide assay. Next, cytotoxic effect of cisplatin was measured using the resazurin assays (PrestoBlue, Invitrogen). The degree of cisplatin induced cytotoxicity was analyzed following pre-treatment of NPC cells with poly I:C and YM-155. Mann Whitney test was used to evaluate the statistical significance between groups.

**Results and discussion** Both HONE1 and C-666 cells expressed TLR3 and survivin on flow cytometry. Poly I:C (0.1-100 μg/ml) treated cells do not affect the expression of TLR3 but induce modest apoptosis (10-15 %) of NPC cells. YM-155 significantly induced apoptosis in a dose-dependent manner with an IC_50_ value of 50nM. Combination therapy with poly I:C and YM-155 enhanced NPC cells apoptosis synergistically compared to any type drug alone (p < 0.050). Cytotoxic effect of cisplatin was significantly enhanced following prê-treatment with YM-155 compared to untreated cells (p < 0.001). The addition of poly I:C pre-treatment to NPC cells demonstrated enhancement of cisplatin induced cytotoxicity only at high concentration of poly I:C (100 μg/ml; p < 0.030).

**Conclusion** Poly I:C and YM-155 in combination enhanced the apoptosis of NPC cells. However, YM-155 was more effective in sensitizing NPC cells to cisplatin induced cytotoxicity compared to poly I:C. Targeting the apoptosis pathway in combination with cisplatin in NPC merits further study.

## O26 The resistance mechanism of nasopharyngeal cancer stem cells to cisplatin through expression of CD44, Hsp70, p53 (wild type), Oct-4, and ß-catenin encoded-genes

### Achmad C Romdhoni^2^, Fedik A Rantam^2,3^, Widodo Ario Kentjono^1^

#### ^1^Department of Otorhinolaryngology Head and Neck Surgery, Faculty of Medicine, Universitas Airlangga, Surabaya, Indonesia; ^2^Stem Cell Laboratory, Institute of Tropical Disease, Universitas Airlangga, Surabaya, Indonesia; ^3^Department of Virology, Faculty of Veterinary, Universitas Airlangga, Surabaya, Indonesia

##### **Correspondence:** Achmad C Romdhoni (romdhoni_ent@yahoo.com) – Department of Otorhinolaryngology Head and Neck Surgery, Faculty of Medicine, Universitas Airlangga, Surabaya, Indonesia

**Introduction** Chemotherapy does not always give a good response in patients with nasopharyngeal carcinoma (NPC). Approximately 60-70 % of the patients showed partial response, while the other 15-25 % showed no response. Residual tumors are often found in patients who receive cisplatin-based regimen. This clinical resistance is generally undesirable because it may develop into a tumor recurrence and poor prognosis. Some researchers proved that such cases were due to the cancer stem cell.

**Aim** This study was conducted to reveal the mechanism of NPC stem cell resistance to cisplatin through expression of CD44, Hsp70, p53 (wild type), Oct-4, and ß-catenin encoded-genes.

**Method** The study was an in vitro experimental laboratory, carried out through four stages: 1) isolation and culture of NPC stem cells obtained from the biopsies of NPC patients; 2) characterization of NPC stem cells (CD44^+^); 3) determination of the concentration of cisplatin used in the study; and 4) analysis of NPC stem cell resistance to cisplatin. The statistical analysis was performed using ANOVA.

**Results and discussion** After administration of cisplatin the expression of the encoded-genes was significantly increased (CD44, p < 0.0001, Hsp70, p < 0.0001, Oct-4, p = 0.003, and p53 (wild type), p = 0.000); but not ß-catenin (p = 0.270). Furthermore, through regression analysis it was shown that Hsp70 (p = 0.002) and CD44 (p = 0.029) were strongly affected the cell death. This study suggested that the increasing expression of CD44, Hsp70, p53 (wild type), and Oct-4 influenced NPC stem cell resistance to cisplatin.

## P1 Prevalence of nasopharyngeal carcinoma patients at Departement of Otorhinolaringology-Head and Neck Surgery, Dr. Hasan Sadikin general hospital, Bandung, Indonesia in 2010-2014

### Deasy Z Madani, Nur Akbar, Agung Dinasti Permana

#### Department of Otorhinolaringology-Head and Neck Surgery, Faculty of Medicine, Padjajaran University/Dr.Hasan Sadikin General Hospital, Bandung, Indonesia

##### **Correspondence:** Deasy Z Madani (deasymadani@gmail.com) – Department of Otorhinolaringology-Head and Neck Surgery, Faculty of Medicine, Padjajaran University/Dr.Hasan Sadikin General Hospital, Bandung, Indonesia

**Introduction** Nasopharyngeal carcinoma is a disease with a certain geographical and ethnic distribution around the world.

**Aim** The present study aimed to determine the prevalence of patients with nasopharyngeal carcinoma presenting at the Otorhinolaringology-Head and Neck Department, Dr. Hasan Sadikin General Hospital Bandung between 2010-2014.

**Methods** A descriptive analysis with sampling method was conducted on data of NPC patients from medical records at Dr. Hasan Sadikin General Hospital Bandung between 2010-2014.

**Results and discussion** Total patient visits at the Otorhinolaringology-Head and Neck Surgery Department, Dr. Hasan Sadikin General Hospital Bandung in the period was 2250. From a total of 692 patients with NPC, 426 data were included, comprising of 265 males and 161 females. Most subjects were of elementary school background (45 %) and with a peak age group of 46-55 years (29.6 %). Most subjects were male (62 %) with a diverse work background, while most females were housewives (38 %). The most histopathology type was undifferentiated carcinoma (57.3 %).

**Conclusion and suggestion** Local NPC patients were dominated by individuals with middle age, older men, lower educational background and tumors with undifferentiated type.

## P2 Case report on pediatric nasopharyngeal carcinoma at Dr. Sardjito Hospital, Yogyakarta

### Camelia Herdini^1^, Sagung Rai Indrasari^1^, Jajah Fachiroh^2,3^, Dwi Hartati^3^, T Baning Rahayudjati^4^

#### ^1^Department of Otolaryngology Head and Neck Surgery, Faculty of Medicine, Universitas Gadjah Mada/Dr Sardjito General Hospital, Yogyakarta, Indonesia; ^2^Department of Histology and Cell Biology, Faculty of Medicine, Universitas Gadjah Mada, Yogyakarta, Indonesia; ^3^Molecular Biology Laboratory, Faculty of Medicine, Universitas Gadjah Mada, Yogyakarta, Indonesia; ^4^Department of Public Health, Faculty of Medicine, Universitas Gadjah Mada, Yogyakarta, Indonesia

##### **Correspondence:** Camelia Herdini (camellia_herdini@yahoo.com) – Department of Otolaryngology Head and Neck Surgery, Faculty of Medicine, Universitas Gadjah Mada/Dr Sardjito General Hospital, Yogyakarta, Indonesia

**Introduction** NPC is rare, accounting for only 1-3 % of all pediatric malignancies. Its incidence varies widely in different regions, reflecting interactions between genetic and environmental factors.

**Aims** To describe clinical characteristic and environmental exposures of two pediatric NPC cases.

**Methods** Case report.

**Result** We reported two cases from Dr. Sardjito Hospital Yogyakarta, Indonesia recruited in 2014. A 10-year-old boy and a 12-year-old girl have already visited general practitioners several times with flu-like complaints. After several unsuccessful attempts at medication, visible lumps in the neck then appeared and they were referred to Dr. Sardjito Hospital. Flexible nasal endoscopy for both patients showed soft tissue masses in the nasopharynx. Histopathology examination revealed undifferentiated carcinoma. The stages were T3N3aM0 and T3N2M0. From the questionnaire given to the parents, it was shown that both patients had high consumption of instant noodles, grilled food, preserved meat, and soy sauce. Additionally, on a daily basis, the boy was exposed to burning firewood and was a passive smoker, while the girl was exposed to wood dust and the use of insecticide spray at home on a daily basis. IgA-EBV ELISA showed that results of both childrens’ parents and the boy were below cut off (< 0.41), but not the girl (0.84).

**Future plan** To collect more data about NPC in children, either clinical presentation or epidemiology aspect to identify risk factors, as well as improving awareness on unspecified complaint among children to find early onset of NPC.

**Consent to publish**

Written informed consent for publication of this clinical details and/or clinical images was obtained from the patient/parent/guardian/relative of the patient. A copy of the consent form is available for review by the Editor of this journal.

## P3 Report on loco regionally advanced nasopharyngeal cancer patients treated with induction chemotherapy followed by concurrent chemo-radiation therapy

### Iswandi Darwis^1^, Susanna Hilda Hutajulu^2^, Bambang Hariwiyanto^3^, Wigati Dhamiyati^4^, Ibnu Purwanto^2^, Kartika Widayati Taroeno-Hariadi^2^, Johan Kurnianda^2^

#### ^1^Study Program of Specialty in Internal Medicine, Department of Internal Medicine, Faculty of Medicine, Universitas Gadjah Mada, Yogyakarta, Indonesia; ^2^Division of Hematology and Medical Oncology, Faculty of Medicine, Universitas Gadjah Mada/Dr Sardjito General Hospital, Yogyakarta, Indonesia; ^3^Department of Otorhinolaryngology Head and Neck Surgery, Faculty of Medicine, Universitas Gadjah Mada/Dr Sardjito General Hospital, Yogyakarta, Indonesia; ^4^Division of Radiotherapy, Department of Radiology, Faculty of Medicine, Universitas Gadjah Mada/Dr Sardjito General Hospital, Yogyakarta, Indonesia

##### **Correspondence:** Iswandi Darwis (iswandi.darwis@gmail.com) – Study Program of Specialty in Internal Medicine, Department of Internal Medicine, Faculty of Medicine, Universitas Gadjah Mada, Yogyakarta, Indonesia

**Introduction** Concurrent chemo-radiation therapy (CCRT) showed a significant improvement in disease control and clinical outcome in patients with nasopharyngeal carcinoma (NPC) with locoregional advanced disease. Effective induction chemotherapy has the potential to downsize locoregional disease prior to definitive treatment.

**Aim** This study aimed to analyze the survival rate of NPC patients who received induction chemotherapy followed by CCRT.

**Methods** An observational study was conducted on data of NPC patients diagnosed at the local setting between January 2007 and December 2011 with stage II-IVB. Twelve out of 16 (75 %) patients completed 1st-line treatment schedule of induction chemotherapy with docetaxel, cisplatin and 5-FU (TPF) 3 cycles followed by CCRT with carboplatin 1.5 AUC day 1 and concurrent radiotherapy in day 1-5 weekly for 7 cycles.

**Results** The median age was 51.5 (37-71) years. All cases had WHO type III pathology features and 8 (66.7 %) patients had bilateral enlargement masses. Most of cases were males (10, 83.3 %). The median of body mass index (BMI) was 22.5 (15.7-27.5). There were 4 (33.3 %) cases with stage II-III and 8 (66.7 %) cases with stage IVA-IVB. Three-year survival rate of cases was 33.3 %. The frequency of patients achieving three-year survival was more frequent in females compared to males (50 % vs 30 %, p = 0.570), in patients with BMI ≤ 22.5 compared to >22.5 (50 % vs 16.67 %, p = 0.270), and in patients with stage II-III compared to IVA-IVB (50 % vs 25 %, p = 0.400).

**Conclusion** In local patients receiving induction chemotherapy followed by CCRT, three- year survival was more frequently achieved by females, individuals with BMI ≤22.5 and stage II-III, even though no statistical significance reached.

## P4 Sex and age differences in the survival of patients with nasopharyngeal carcinoma

### Sindhu Wisesa^1^, Mardiah Suci Hardianti^2^, Susanna Hilda Hutajulu^2^, Kartika Widayati Taroeno-Harijadi^2^, Ibnu Purwanto^2^, Camelia Herdini^3^, Wigati Dhamiyati^4^, Johan Kurnianda^2^

#### ^1^Department of Anatomy, Faculty of Medicine, Jenderal Soedirman University, Purwokerto, Indonesia; ^2^Division of Hematology and Medical Oncology, Department of Internal Medicine, Faculty of Medicine, Universitas Gadjah Mada/Dr Sardjito General Hospital, Yogyakarta, Indonesia; ^3^Department of Otorhinolaryngology Head and Neck Surgery, Faculty of Medicine Universitas Gadjah Mada/Dr Sardjito General Hospital, Yogyakarta, Indonesia; ^4^Division of Radiotherapy, Department of Radiology, Faculty of Medicine Universitas Gadjah Mada/Dr Sardjito General Hospital, Yogyakarta, Indonesia

##### **Correspondence:** Sindhu Wisesa (sindhu.wisesa@gmail.com) – Department of Anatomy, Faculty of Medicine, Jenderal Soedirman University, Purwokerto, Indonesia

**Introduction** The incidence of nasopharyngeal carcinoma (NPC) in male patients was significantly higher than in female. Several studies showed sex and age have strong influence on a patient’s survival in solid tumor. Their prognostic impact in NPC patients remains unclear.

**Aim** To investigate the effect of sex and age difference on overall survival and clinical outcomes of NPC patients.

**Methods** A retrospective study included data of NPC patients at Dr. Sardjito Hospital between 2007-2011. From 333 NPC patients, 10 were excluded due to incomplete information of histopathology data. Kaplan-Meier and cox regression method were used to calculate overall survival (OS). Kruskal-Wallis analysis was used to define the relationship among variables.

**Results and discussion** Male patients were two-fold higher in frequency than female patients (225, 9.7 % and 98, 30.3 %, respectively). Female patients had a higher median OS compared with male patients (2.62 years versus 1.24 years; hazard ratio for male patients 1.271), although no statistical significance was reached (p = 0.286). It may be caused by the difference of tumor behavior between male and female that was influenced by their different genotype and hormonal function. The frequency of NPC was higher in older patients (≥ 45 years) than younger patients (<45 years) (62.6 % versus 37.4 %). Older cases had a significant lower median OS (1.45 years versus 2.51 years, hazard ratio for older patients 1.727; p = 0.014). The correlation between age and OS was stronger in male patients (p = 0.010) than in female patients (p = 0.577). Compared to younger patients, older patients had more comorbidities and poorer baseline characteristics such as anemia (p = 0.106) and low creatinine clearance (p = 0.390). They also experienced more adverse reactions during treatment course including myelosuppression (p = 0.004), nephrotoxicity (p = 0.007), electrolyte disturbance (p = 0.008), diarrhea (p = 0.006), and nausea and vomiting (p = 0.050) that might contribute to the lower OS. Multivariate analysis showed that age was an independent prognostic factor for the OS (p = 0.014).

**Conclusion** Male NPC patients and cases with older age had a lower OS compared to their counterparts. Furthermore, older age was a negative independent prognostic factor for the survival.

## P5 Impact of delayed diagnosis and delayed therapy in the treatment outcome of patients with nasopharyngeal carcinoma

### Khoirul Anwar^1^, Susanna Hilda Hutajulu^2^, Sagung Rai Indrasari^3^, Sri Retna Dwidanarti^4^, Ibnu Purwanto^2^, Kartika Widayati Taroeno-Hariadi^2^, Johan Kurnianda^2^

#### ^1^Study Program of Specialty in Internal Medicine, Department of Internal Medicine, Faculty of Medicine, Universitas Gadjah Mada, Yogyakarta, Indonesia; ^2^Division of Hematology and Medical Oncology, Department of Internal Medicine, Faculty of Medicine, Universitas Gadjah Mada/Dr Sardjito General Hospital, Yogyakarta, Indonesia; ^3^Department of Otorhinolaryngology Head and Neck Surgery, Faculty of Medicine, Universitas Gadjah Mada/Dr Sardjito General Hospital, Yogyakarta, Indonesia; ^4^Division of Radiotherapy, Department of Radiology, Faculty of Medicine, Universitas Gadjah Mada/Dr Sardjito General Hospital, Yogyakarta, Indonesia

##### **Correspondence:** Khoirul Anwar (vierithegreat@gmail.com) – Study Program of Specialty in Internal Medicine, Department of Internal Medicine, Faculty of Medicine, Universitas Gadjah Mada, Yogyakarta, Indonesia

**Introduction** Nasopharyngeal carcinoma (NPC) is the most common malignancy of head and neck in Indonesia. Early detection is difficult and early diagnosis and treatment are frequently delayed.

**Aim** This study evaluated presentation and treatment outcomes of patients with NPC who had delayed diagnosis and therapy in a tertiary referral centre, in Yogyakarta, Indonesia.

**Methods** One-hundred and three patients diagnosed as NPC between 2007 and 2011 were retrospectively evaluated. Baseline data included age, gender, type of insurance, disease stage, presenting symptoms, symptom-to-visit interval (SVI), and diagnosis-to-treatment interval (DTI). Treatment outcomes and overall survival (OS) were analyzed.

**Results and discussion** Of 103 patients, 87 (94.1 %) had an advanced disease, 23 (22.3 %) had distant metastases, and 48 (46.6 %) presented with neck mass as early symptoms. The mean of SVI was 9.9 months and the mean DTI of was 53 days. From 53 patients having SVI more than 6 months, 22 (41.5 %) were with low education, covered by government insurance for the poor people (28, 52.8 %), lived outside of Yogyakarta (31, 58.5 %) and had neck mass as early symptoms (27, 50.9 %). Out of 105 patients planned for first line treatment, 22 patients did not complete the treatment due to death (2, 1.9 %), adverse event (1, 1 %), financial problems (1, 1 %), lost to follow up (16, 15.2 %) and unknown reason (2, 1.9 %). In 83 patients who completed the treatment, only 49 records of treatment response were available. Complete response was achieved in 10 patient (20.4 %) and partial response in 11 patients (22.4 %). Twenty patients (40.8 %) were in stable disease while 8 patients (16.3 %) showed disease progression. Median of OS was 17.23 months (ranged from 0.7-60.9 months) and 3-year survival rate was 16.5 %.

**Conclusion and future direction** Treatment outcomes in NPC patients who had delayed diagnosis and therapy was poor. Level of education, health insurance coverage, distance from referral centre, and non-specific early sign of NPC may contribute to the delay of SVI. The practitioners must improve their capabilities in educating patient for symptoms of NPC so that cases with earlier stage can be identified. Furthermore, patients should be better educated about the importance of immediate treatment initiation in order to achieve optimum results.

## P6 Anaysis of pretreatment anemia in nasopharyngeal cancer patients undergoing neoadjuvant therapy

### Dominicus Wendhy Pramana^1^, Susanna Hilda Hutajulu^2^, Bambang Hariwiyanto^3^, Wigati Dhamiyati^4^, Ibnu Purwanto^2^, Kartika Widayati Taroeno-Hariadi^2^, Johan Kurnianda^2^

#### ^1^Study Program of Specialty in Internal Medicine, Department of Internal Medicine, Faculty of Medicine, Universitas Gadjah Mada, Yogyakarta, Indonesia; ^2^Division of Hematology and Medical Oncology, Department of Internal Medicine, Faculty of Medicine, Universitas Gadjah Mada/Dr Sardjito General Hospital, Yogyakarta, Indonesia; ^3^Department of Otorhinolaryngology Head and Neck Surgery, Faculty of Medicine, Universitas Gadjah Mada/Dr Sardjito General Hospital, Yogyakarta, Indonesia; ^4^Division of Radiotherapy, Department of Radiology, Faculty of Medicine, Universitas Gadjah Mada/Dr Sardjito General Hospital, Yogyakarta, Indonesia

##### **Correspondence:** Dominicus Wendhy Pramana (drwendhypramana@gmail.com) – Study Program of Specialty in Internal Medicine, Department of Internal Medicine, Faculty of Medicine, Universitas Gadjah Mada, Yogyakarta, Indonesia

**Introduction** Neoadjuvant therapy with cisplatin and 5-fluorouracyl (5-FU) has been a reasonable treatment option for advanced nasopharyngeal cancer (NPC) in regions where delayed radiotherapy initiation is relatively common, such as Yogyakarta. As pretreatment anemia has been observed to negatively influence patient’s survival in various cancers, the present study analyzed its impact in NPC patients undergoing neoadjuvant treatment.

**Aim** This study aimed to determine whether pretreatment anemia has a prognostic role in NPC patients undergoing neoadjuvant therapy.

**Methods** The data of 40 patients diagnosed as NPC between January 2007 and December 2011 and who underwent neoadjuvant therapy with cisplatin and 5-FU were retrospectively reviewed. Baseline hemoglobin concentration were evaluated at diagnosis. Hemoglobin concentration of less 13 g/dL for males and less than 12 g/dL for females were defined as anemia. Along with other known prognostic factors (age and sex), the prognostic value of pretreatment anemia was analyzed. Overall survival (OS) was assessed using the Kaplan-Meier method. Univariate analyses was used to evaluate prognostic significance.

**Results** Pretreatment anemia was observed in 12 (30 %) patients. Median OS was 32.2 months (ranged from 28.2-37.5 months). Patients with pretreatment anemia showed significantly lower median OS than those with normal hemoglobin concentration (21.3 vs 34.5 months, p = 0.044). In univariate analysis, cases with pretreatment anemia had lower median OS, although significance was not reached (HR = 2.68; 95 % confidence interval 0.98-7.28).

**Conclusion** Local NPC patients who underwent neoadjuvant therapy and had pretreatment anemia showed a worse median OS compared to those without anemia. However, the pretreatment condition did not significantly predicted patient’s survival.

## P7 Results of treatment with neoadjuvant cisplatin-5FU in locally advanced nasopharyngeal carcinoma: a local experience

### Diah Ari Safitri^1^, Susanna Hilda Hutajulu^1^, Camelia Herdini^2^, Sri Retna Dwi Danarti^3^, Ibnu Purwanto^1^, Kartika Widayati Taroeno-Hariadi^1^, Johan Kurnianda^1^

#### ^1^Division of Hematology and Medical Oncology, Department of Internal Medicine, Faculty of Medicine, Universitas Gadjah Mada/DrSardjito General Hospital, Yogyakarta, Indonesia; ^2^Department of Otorhinolaryngology Head and Neck Surgery, Faculty of Medicine, Universitas Gadjah Mada/DrSardjito General Hospital, Yogyakarta, Indonesia; ^3^Division of Radiotherapy, Department of Radiology, Faculty of Medicine, Universitas Gadjah Mada/DrSardjito General Hospital, Yogyakarta, Indonesia

##### **Correspondence:** Diah Ari Safitri (ifidas2@gmail.com) – Division of Hematology and Medical Oncology, Department of Internal Medicine, Faculty of Medicine, Universitas Gadjah Mada/DrSardjito General Hospital, Yogyakarta, Indonesia

**Introduction** The significance of the problem of radiotherapy waiting lists in local patients has been documented. Delays in initiating radiation treatment may result in poorer treatment outcomes. Even though neoadjuvant chemotherapy had not been considered a standard approach for locally advanced nasopharyngeal carcinoma (NPC), it may be given while waiting for the radiotherapy schedule. The platinum-based chemotherapy combined with 5-fluorouracil (5-FU) was the most frequently used for NPC. Data on NPC patients receiving neoadjuvant cisplatin 5-FU chemotherapy followed by radiotherapy at our cancer clinic had not been comprehensively analyzed.

**Aim** To analyze the survival rate of NPC patients who received neoadjuvant cisplatin-5-FU chemotherapy followed by radiotherapy.

**Methods** An observational study was conducted on data of NPC patients diagnosed at Dr Sardjito Hospital Yogyakarta between January 2007 and December 2011. Patients with stage IIB-IVB disease who received cisplatin IV infusion 100 mg/m^2^ on day 1 and 5-FU IV infusion 1000 mg/m^2^/24 hours on days 1 to 4, or 1 to 5 as neoadjuvant therapy were analyzed.

**Results** Fourty-three (n = 43) stage IIB-IVB NPC were analyzed. The age of subjects ranged from 17 to 76 years (median 48 years), with male predominant (67.40 %). Stage III, IVA, and IVB were 17 (39.54 %), 6 (13.95 %) and 20 (46.51 %) subjects, respecively. WHO type III was predominant (95.35 % cases). The radiation dose ranged from 18 to 70 Gy (median 66 Gy). A total of 37 (86.05 %) subjects received 3 cycles of cisplatin-5FU, while 4 (9.31 %) received 4-6 cycles and 2 (4.64 %) received 1 cycle only. The median survival was 34.63 months. The 3 year- and 5 year-survival rates were 45.30 % and 37.80 %, respectively. The median follow up period was 23.27 months. When compared, stage III and IV subjects had similar median survival (36.50 months versus 33.93 months, p = 0.295).

**Conclusion** With neoadjuvant cisplatin and 5-FU, survival beyond 5 years was possible in our local setting. Treatment adherence, patient’s obedience on follow up, and improved radiologic facilities may contribute to a better survival rate.

## P8 Geriatrics with nasopharyngeal cancer

### Suryo A. Taroeno^1^, Sindhu Wisesa^2^, Kartika Widayati Taroeno-Hariadi^1^, Ibnu Purwanto^1^, Bambang Hariwiyanto^3^, Wigati Dhamiyati^4^, Johan Kurnianda^1^

#### ^1^Division of Hematology and Medical Oncology, Department of Internal Medicine, Faculty of Medicine, Universitas Gadjah Mada/Dr Sardjito General Hospital, Yogyakarta, Indonesia; ^2^Department of Anatomy, Faculty of Medicine, Jenderal Soedirman University, Purwokerto, Indonesia; ^3^Department of Otorhinolaryngology Head and Neck Surgery, Faculty of Medicine, Universitas Gadjah Mada, Yogyakarta, Indonesia; ^4^Division of Radiotherapy, Department of Radiology, Faculty of Medicine, Universitas Gadjah Mada, Yogyakarta, Indonesia

##### **Correspondence:** Suryo A Taroeno (suryotaroeno@gmail.com) – Division of Hematology and Medical Oncology, Department of Internal Medicine, Faculty of Medicine, Universitas Gadjah Mada/Dr Sardjito General Hospital, Yogyakarta, Indonesia

**Introduction** Aging patients with cancer commonly pose special problems. As a special population of cancer patients, they may have specific clinical features and treatment outcomes.

**Aim** To explore the database of nasopharyngeal cancer (NPC) geriatric patients in the local centre, regarding the specific clinical features and the treatment outcomes.

**Methods** Retrospective analysis was done on data of NPC patients diagnosed between 2007-2011 at Dr Sardjito Hospital, Yogyakarta Indonesia (organ-specific cancer registry). Geriatric patients were defined as cases of ≥60 years old at first visits. The effect of age and treatment on patients’ survival were analyzed.

**Result and discussion** During the period, data from 65 geriatric NPC patients were recorded in the database. Mean age was 66.6 years old (range 60-85 years old). The majority of patients presented at late stage of the disease (89.2 % in stage III or IV). The median time of ‘symptom-to-first-visit’ was 6 months. The active cancer treatments (chemoradiation or chemotherapy alone) were given to 45 (67 %) patients. The adherence for completion of the planned treatments was 60 %. Non-adherence (33 %) was due to lost to follow up. The rate of 1-year survival was 32 %. There was no significant difference found between the survivors and the non-survivors (p = 0.406, Mann-Whitney test), as well as Karnofsky performance status (p = 0.943, Mann-Whitney test). There was a slight association between active treatment and survival in one year (p = 0.047, Chi-square), although this trend was not maintained in survival analysis (p = 0.182, log-rank test). ‘Late-stage presentation’ was apparently the main problem in geriatric NPC patients, putting them in the commonly incurable stage of the disease, associated with low survival rate. A comprehensive strategy for disease down-staging, beside active treatments, may serve as a better management approach.

## P9 Correlation of lymphocyte to monocyte and neutrophil to lymphocyte ratio to the response of cisplatin chemoradiotheraphy in locally advance nasopharyngeal carcinoma

### I. Wijaya, A Oehadian, D Prasetya

#### Division of Hematology and Medical Oncology, Department of Internal Medicine, Dr Hasan Sadikin Hospital, Padjadjaran University, Bandung, Indonesia

##### **Correspondence:** I. Wijaya (indrawijayaipd@gmail.com) – Division of Hematology and Medical Oncology, Department of Internal Medicine, Dr Hasan Sadikin Hospital, Padjadjaran University, Bandung, Indonesia

**Introduction** Nasopharyngeal cancer (NPC) is radiosensitive and chemosensitive. However, the response to cisplatin chemoradiation for an individual patient is unpredictable. The lack of factors to predict the treatment response remains a major problem. Elevated lymphocyte to monocyte ratio (LMR) and neutrophil to lymphocyte (NLR) has been reported to be associated with prognosis in NPC.

**Aim** The aim of this study was to investigate the correlation of LMR and NLR to complement as predictor in the assessment of response to cisplatin chemoradiation in NPC.

**Methods** We collected data from a prospective cohort study in Dr Hasan Sadikin General Hospital, Bandung. The study was performed from October 2012 to October 2013. Clinical examination and CT scan were performed before and after chemoradiation to determine the therapy response based on *Response Evaluation Criteria in Solid Tumors (*RECIST*)* criteria. The relationship between treatment response and pretreatment LMR and NLR was analyzed using point bi serial correlation test.

**Result and discussion** We enrolled 35 patients, and 5 were excluded from the study due to incomplete treatment. Thirteen (13) out of 30 patients were at stage IVA and 17 patients were at stage IVB. There were 23 (76 %) responder patients (1 patient with complete, 1 stable and 21 partial response), and 7 (24 %) unresponder patients (with progressive disease). NLR was significantly correlated with response of cisplatin chemoradiation in patients with locally advanced NPC (r = -0.330; p = 0.037), but not LMR (r = -0.171, p = 0.187).

**Conclusion** In locally advanced NPC patients who were treated with cisplatin chemoradiation, NLR may be a predictive factor, but not LMR.

## P10 Prediction of nasopharyngeal carcinoma risk by Epstein-Barr virus seromarkers and environmental co-factors: the gene-environment interaction study on nasopharyngeal carcinoma in Taiwan

### Wan-Lun Hsu^1^, Yin-Chu Chien^1^, Kelly J Yu^2^, Cheng-Ping Wang^3^, Ching-Yuan Lin^4^, Yung-An Tsou^5^, Yi-Shing Leu^6^, Li-Jen Liao^7^, Yen-Liang Chang^8,9^, Jenq-Yuh Ko^3^, Chun-Hun Hua^5^, Ming-Shiang Wu^10,11^, Chu-Hsing Kate Hsiao^12^, Jehn-Chuan Lee^6^, Ming-Hsui Tsai^5^, Skye Hung-Chun Cheng^13^, Pei-Jen Lou^3^, Allan Hildesheim^14^, Chien-Jen Chen^1,12^

#### ^1^Genomics Research Center, Academia Sinica, Taipei, Taiwan; ^2^Division of Cancer Prevention, National Cancer Institute, Bethesda, MD, USA; ^3^Department of Otolaryngology, National Taiwan University Hospital and National Taiwan University College of Medicine, Taipei, Taiwan; ^4^Department of Head and Neck Surgery, Koo Foundation Sun Yat-Sen Cancer Center, Taipei, Taiwan; ^5^Department of Otorhinolaryngology-Head and Neck Surgery China Medical University Hospital, Taichung, Taiwan; ^6^Department of Otolaryngology Head & Neck Surgery, Mackay Memorial Hospital, Taipei, Taiwan; ^7^Department of Otolaryngology, Far Eastern Memorial Hospital, Taipei, Taiwan; ^8^Department of Otolaryngology Head and Neck Surgery, Cathay General Hospital, Taipei, Taiwan; ^9^School of Medicine, Fu Jen Catholic University, Taipei, Taiwan; ^10^Department of Internal Medicine, National Taiwan University Hospital, Taipei, Taiwan; ^11^Health Management Center, National Taiwan University Hospital, Taipei, Taiwan; ^12^Graduate Institute of Epidemiology and Preventive Medicine College of Public Health, National Taiwan University, Taipei, Taiwan; ^13^Department of Radiation Oncology, Koo Foundation Sun Yat-Sen Cancer Center, Taipei, Taiwan; ^14^Division of Cancer Epidemiology and Genetics, National Cancer Institute, Bethesda, MD, USA

##### **Correspondence:** Wan-Lun Hsu (lun0112@ms26.hinet.net) – Genomics Research Center, Academia Sinica, Taipei, Taiwan

**Introduction** Epstein-Barr virus (EBV) infection is known to be associated with the development of nasopharyngeal carcinoma (NPC). Cofactors include long-term cigarette smoking, family history and diet marker. The evaluation of EBV markers and other risk factors may help in future identification of risks. However, it is still unclear whether EBV alone or combined with other risk factors is sufficientfor NPC riskprediction.

**Aims** This study aims to assess the predictability of NPC risk using a case-control study by evaluating the combination of anti-EBV markers with and without known environmental factors.

**Methods** A case-control study was conducted in Taiwan. A total of 699 histologically confirmed incident NPC cases and 1,359 controls frequency matched to cases on sex, age were recruited between July 2010 and December 2014.A questionnaire inquiring socio-demographic characteristics, cigarette smoking, diet consumption, family history of NPC was used in the trained nurse interview. Blood specimens were tested for anti-EBV VCA IgA and anti-EBV EA-EBNA1 IgA. The predictive accuracy and 95 % confidence interval (CI) was evaluated using the area under the receiver operating characteristic curve (AUROC). Several risk models were used to combine different sets of NPC predictors.

**Result and discussion** The AUROC model predicted NPC poorly based on only environmental factors (AUROC = 0.647, 95 % CI = 0.622-0.673) and NPC family history (AUROC = 0.544, 95 % CI = 0.530-0.559). The corresponding AUROCs (95 % CI) were 0.885 (0.870-0.900) and 0.911 (0.897-0.924) for anti-EBV VCA IgA and anti-EBV EA-EBNA1 IgA, respectively. The AUROCs (95 % CI) were 0.912 (0.897-0.926) for anti-EBV VCA IgA and 0.936 (0.924-0.948) for anti-EBV EA-EBNA1 IgA after adjusting for family history and environmental factors. Anti-EBV marker and other factors may be combined into useful risk models for predicting NPC risk. Additional analyses are on going.

## P11 Non-viral risk factors for nasopharyngeal carcinoma in West Sumatra, Indonesia

### Sukri Rahman, Bestari J Budiman, Novialdi, Rahmadona, Dewi Yuri Lestari

#### Department of Otorhinolaryngology Head and Neck Surgery, Faculty of Medicine, Andalas University, Padang, Indonesia

##### **Correspondence:** Sukri Rahman (sukri_rahman@yahoo.com) – Department of Otorhinolaryngology Head and Neck Surgery, Faculty of Medicine, Andalas University, Padang, Indonesia

**Introduction** Nasopharyngeal carcinoma (NPC) is a unique cancer with incidence which varies widely according to geographic location and ethnic background. Many studies have shown that the etiology of NPC is multifactorial, including genetic, viral infection and environmental factors. NPC is a frequent cancer in Indonesia, and, specifically, the most common head and neck cancer in West Sumatra. Indonesia has diverse ethnic groups with various lifestyles and local food. Minangkabau is an ethnic group that dominates the population of West Sumatra.

**Aims** This study aimed to determine whether non-viral factors increased the risk of NPC in this population.

**Methods** A case-control study was conducted in 33 newly diagnosed NPC cases and 33 sex- and age- (±5 years) matched controls. Data were collected for demographic characteristics and exogenous factors using questionnaire through face to face interviews. Odds ratio (ORs) and corresponding 95 % confidence intervals (CIs) were estimated.

**Results and discussion** The present study demonstrated that exposure to smoke of anti-mosquito coils (OR = 3.54; 95 % CI 1.28-9.80), exposure to wood dust (OR = 3.63; 95 % CI 1.02-12.93), and family history of NPC (OR = 2.06; 95 % CI 1.60-2.66) were associated with an increased risk of NPC. Salted fish consumption was more frequent in cases than controls, but the difference was not significant (OR = 2.51; 95 % CI 0.90-7.00). There were no associations between NPC risk and preserved meat (OR = 3.20; 95 % CI 0.31-32.47), tobacco smoking (OR = 2.37; 95 % CI 0.88-6.35), alcohol consumption (OR = 1.24; 95 % CI 0.34-4.56), wood fire (OR = 2.30; 95 % CI 0.80-6.60), herbal medicine (OR = 0.31; 95 % CI 0.31-3.17), past history of chronic disease in ear, nose, and throat (OR = 1.69; 95 % CI 0.52-544) and pesticides exposure (OR = 2.60; 95 % CI 0.91-7.44).

**Conclusion** Our results suggested that the use of anti-mosquito coils and exposure to wood dust are associated with the risk of NPC development in the West Sumatra population.

## P12 New prototype Vidas EBV IgA quick: performance on Chinese and Moroccan populations

### C. Yin, A Foussadier, E Blein, C Chen, N Bournet Ammour, M Khiatti, S Cao

#### BioMerieux (Shanghai) biotech company limited, Pudong, Shanghai, China

##### **Correspondence:** C. Yin (Coco.yin@biomerieux.com) – BioMerieux (Shanghai) biotech company limited, Pudong, Shanghai, China

**Introduction** Nasopharyngeal Cancer (NPC) is also named “Guangdong cancer”, a rare malignant tumour with most incidence in five southern Chinese provinces (population in 2013: 2.4 billion). NPC diagnosis is usually made by nasopharyngoscopy and biopsy at advanced stage, when the cancer seriously impacts the clinical outcome and the patient quality of life. Earlier diagnosis of concerned population in endemic areas is an important unmet need. EBV IgA biomarkers are currently used for screening.

**Aims** The aim of the study was to evaluate the performance of a new prototype automated VIDAS® EBV IgA tests for the screening of the patient with NPC.

**Materials and methods** Characterized samples coming from Ghangzhou, China (NPC patients (N = 150) and controls (N = 150)) and coming from Casablanca, Morocco (NPC patients (N = 67) and controls (N = 100)) are tested with the new Vidas prototype.

**Results and discussion** On characterized sera samples, the sensitivities of VIDAS EBV IgA, on Chinese samples, on Moroccan samples and globally were respectively 93.3 % (88.1 %-96.8 %), 86.6 % (76.0-93.7) and 91.5 % (86.7-94.7) and the specificities of VIDAS EBV IgA on Chinese samples, on Moroccan samples and globally were respectively 91.1 %(85.5-95.0), 92.0 %(84.8- 96.5) and 91.4 % (87.3-94.6). The new Vidas EBV IgA prototype can give a result in 25 minutes using the automated Vidas system. The new VIDAS EBV IgA quick presented good performance in accordance with the patients’ clinical status. VIDAS EBV reagents will be a good alternative enabling single testing as well as series to currently used manual method for NPC diagnosis.

## P13 The expression of EBV-LMP1 and VEGF as predictors and plasma EBV-DNA levels as early marker of distant metastasis after therapy in nasopharyngeal cancer

### Dewi Syafriyetti Soeis Marzaini (smfrad@yahoo.com; dewisoeismarzaini@yahoo.com)

#### Department of Radiotheraphy, Dharmais National Cancer Hospital, Jakarta, Indonesia

**Introduction** Distant metastasis after treatment is common in nasopharyngeal cancer (NPC). There are no pre-treatment predictors and early markers for effective treatment response monitoring.

**Aims** This study aimed to evaluate the expression of Epstein-Barr Virus (EBV) latent membrane protein-1 (LMP1) and vascular endothelial growth factor (VEGF) as predictors and plasma EBV-DNA levels as early markers of distant metastasis after treatment.

**Methods** A case-control study was done on patients who had and did not have distant metastasis after therapy. The study was conducted in Dharmais Cancer Center and Cipto Mangunkusumo Hospitals, Jakarta, between January 2006 and December 2007. The expression of EBV-LMP1 and VEGF were done by immunohistochemistry from nasopharyngeal biopsy specimens at diagnosis, whereas plasma EBV-DNA was assayed from the patients’ blood, six months after therapy and was quantified using RT-PCR technique.

**Results and discussion** A total of 53 patients were enrolled in this study; 40 (75 %) of them were men. Patients’ mean age was 49 years. There were 22 (41.5 %) patients with distant metastasis and 31 (58.5 %) patients without distant metastasis after treatment. Mean score of EBV-LMP1 and VEGF expressions were significantly higher in patients with distant metastasis compared to patient without distant metastasis. Measurement of plasma EBV-DNA levels had 95.2 % sensitivity, 96.6 % specificity, and an AUC of 0.962 (95 % CI 0.902-1.023) to detect early treatment failure.

**Conclusion and suggestion** The expression of EBV-LMP1 immunohistologically has a bigger effect than VEGF expression and can be used as predictor for distant metastasis after treatment and therefore should be evaluated at the time of diagnosis. Plasma EBV-DNA level after therapy is highly sensitive as early detection for distant failure and should be done periodically for monitoring the treatment response.

## P14 Characteristics and factors influencing subjects refusal for blood samples retrieval: lesson from NPC case control study in Yogyakarta – Indonesia

### Dwi Hartati^1^, Baning Rahayujati^2^, Camelia Herdini^3^, Jajah Fachiroh^1,4^

#### ^1^Molecular Biology Laboratory, Faculty of Medicine, Universitas Gadjah Mada, Yogyakarta, Indonesia; ^2^Department of Public Health, Faculty of Medicine Universitas Gadjah Mada, Yogyakarta, Indonesia; ^3^Department of Otorhinolaryngology Head and Neck Surgery, Faculty of Medicine Universitas Gadjah Mada/Dr Sardjito General Hospital, Yogyakarta, Indonesia; ^4^Department of Histology and Cell Biology, Faculty of Medicine Universitas Gadjah Mada, Yogyakarta, Indonesia

##### **Correspondence:** Jajah Fachiroh (jajahfachiroh@ugm.ac.id) – Molecular Biology Laboratory, Faculty of Medicine, Universitas Gadjah Mada, Yogyakarta, Indonesia

**Introduction** In an epidemiology survey, data samples can be obtained through questionnaires as well as biosample retrieval. However, subjects who are willing to participate in surveys often refuse to give their biosample.

**Aim** This research aimed to identify the characteristics and factors influencing subjects’ refusal towards peripheral blood sampling on a hospital-based case-control study in Yogyakarta.

**Methods** Analysis was done using analytical descriptive method with cross sectional design. The subjects were controls from hospital-based NPC case-control study in Dr. Sardjito Hospital Yogyakarta (n = 277) recruited in 2014. Demographic questionnaire and peripheral blood sampling were taken at the same time. The difference of population characteristics were observed using *chi square* with two tails p value ≤ 0.05 regarded as significant.

**Results** All subjects could answer the questionnaire well. Male:female ratio was 3.1:1, with the highest age group of 50-60 years old (23.1 %). The highest level of education was university level (35 %). As much as 37.2 % of the respondents were willing to have their peripheral blood sampled. This group was dominated by males (71.8 %), individuals of 31-40 years (28.2 %), and graduated from university (45.6 %). Among those who refused to have their peripheral blood sampled, only 3.4 % said that they were scared of the procedure. The analysis showed that the lower the level of education and the older the age, the higher the rejection for the peripheral blood sampling (p = 0.008 and p = 0.045). In addition, for the blood sampling methods, people with high level of education tended to choose fingerprick compared to blood drawning from the vein (p = 0.001), as they thought that the result would be the same.

**Future plan** Different approaches among different age group and educational background should be applied in order to educate subjects and avoid rejection of participating in epidemiology survey.

## P15 Expression of microRNA BART-7-3p and mRNA PTEN on blood plasma of patients with nasopharyngeal carcinoma

### L. Gunawan^1^, S. Mubarika Haryana^2^, A. Surono^3^, C. Herawati^4^

#### ^1^Department of Biotechnology, Graduate program, Universitas Gadjah Mada, Yogyakarta, Indonesia; ^2^Department of Histology and Biology Molecular, Faculty of Medicine, Univesitas Gadjah Mada, Yogyakarta, Indonesia; ^3^Department of Otorhinolaryngology Head and Neck Surgery, Faculty of Medicine Universitas Gadjah Mada/Dr Sardjito General Hospital, Yogyakarta, Indonesia; ^4^Department of Otorhinolaryngology Head and Neck Surgery, Dharmais Cancer Hospital, Jakarta, Indonesia

##### **Correspondence:** L. Gunawan (lisgunchan@yahoo.co.id) – Department of Biotechnology, Graduate program, Universitas Gadjah Mada, Yogyakarta, Indonesia

**Introduction** Nasopharyngeal Carcinoma (NPC) is an epithelial associated-Epstein-Barr Virus (EBV) infected cancer which has become an epidemic in the Asia region including Indonesia. Recently, the incidence and mortality of NPC cases increased, and there is tendency towards younger ages. The poor diagnostic method for this cancer is one of the main problems for NPC treatment, thus new efficient and effective diagnostic methods are needed. MicroRNA BART-7 is one of the BART family microRNA that is expressed by EBV. MicroRNA BART-7 is observed as one of the potential biomarkers for the detection of NPC. *Phosphatase and tensin homolog* (PTEN) is a human tumor suppressor protein which associated to developments of various types of cancer, including NPC. Mutation and deletion of PTEN gene cause enzimatic inactivation of PTEN protein and lost of tumor suppresion ability of PTEN. MicroRNA BART-7-3p is one of the miRNA that targeted PTEN.

**Aims** This research aims to analyze the expression of MicroRNA BART-7-3p and mRNA PTEN on NPC patient and healthy control.

**Methods** Analysis was performed on 64 plasma of NPC patients, 10 plasma of healthy control and 4 tissues of NPC patients by isolation of total RNA, cDNA synthesis and quantification by qPCR.

**Result and discussion** The expression of MicroRNA BART-7-3p on the plasma NPC patients were higher than the healthy control. On the NPC tissues, the expression of MicroRNA BART-7-3p were very high compared to the plasma healthy control. Additionally, this research also did the measurement of mRNA PTEN which is targeted by MicroRNA BART-7-3p. The expression of mRNA on the plasma NPC patients were lower than the healthy control. Through statistical analysis it was deemed that MicroRNA BART-7-3p and mRNA PTEN has a negative correlation.

## P16 IgA response to native early antigen (IgA-EAext) of Epstein-Barr virus (EBV) in healthy population and nasopharyngeal carcinoma (NPC) patients: the potential for diagnosis and screening tools

### Michael Hartono^1^, Jajah Fachiroh^2,3^, Umi Intansari^4^, Dewi Kartikawati Paramita^2,3^

#### ^1^Study Program of Medicine, Faculty of Medicine, Universitas Gadjah Mada, Yogyakarta, Indonesia; ^2^Department of Histology and Cell Biology, Faculty of Medicine, Universitas Gadjah Mada, Yogyakarta, Indonesia; ^3^Molecular Biology Laboratory, Faculty of Medicine, Universitas Gadjah Mada, Yogyakarta, Indonesia; ^4^Department of Clinical Pathology, Faculty of Medicine, Universitas Gadjah Mada, Yogyakarta, Indonesia

##### **Correspondence:** Dewi Kartikawati Paramita (dkparamita@ugm.ac.id) - Department of Histology and Cell Biology, Faculty of Medicine, Universitas Gadjah Mada, Yogyakarta, Indonesia

**Introduction** Nasopharyngeal carcinoma (NPC) is one of Epstein-Barr virus (EBV) associated malignancy, as observed in the presence of antibody responses to EBV antigens. Development of EBV-based serological tool is important. However, NPC subjects recognize multiple EBV antigens, which require evaluation upon development of an EBV-based diagnostic method using ELISA.

**Aims** To examine the potential of IgA against native EA protein (IgA-EA_ext_) as diagnostic and screening tool, either single or in combination with IgA-[EBNA1 + VCAp18] response.

**Methods** A hundred and twenty (n = 120) histopathologicaly confirmed NPC patients and healthy population were tested to asses the IgA-EA_ext_ response using ELISA method. The mean of IgA-EA_ext_ responses were tested using independent T-test. Kendall’s concordance test was used to examine the concordance between IgA-EA_ext_ and IgA-[EBNA1 + VCAp18] responses. Cut off value (CoV), sensitivity, specificity were determined by using ROC analysis, and also to calculate the positive predictive value (PPV) and negative predictive value (NPV) of IgA-EA_ext._

**Result and discussion** CoV of IgA-EA_ext_ ELISA was 0.274. Approximately 90.76 % (108/119) of NPC patients showed result above CoV; while only 2.5 % (3/120) of healthy individuals showed positive result. The sensitivity, specificity, PPV and NPV of IgA-EA_ext_ were 90.76 %, 97.50 %, 97.29 % and 91.4 %, respectively. When the IgA-EA_ext_ combined with IgA-[EBNA1 + VCAp18], the sensitivity became lower (86.55 %) but the specificity reached 100 %, with PPV and NPV were 100 %, and 88.23 %, respectively.

**Conclusion** IgA-EA_ext_ ELISA is a potential method for NPC diagnosis/screening. When combined with IgA-[EBNA1 + VCAp18] these method can be used as confirmatory test for NPC.

## P17 IgA responses against Epstein-Barr Virus Early Antigen (EBV-EA) peptides as potential candidates of nasopharyngeal carcinoma detection marker

### Akmal Akbar^1^, Jajah Fachiroh^2^, Dewi Kartikawati Paramita^2^

#### ^1^Study Program of Medicine, Faculty of Medicine, Universitas Gadjah Mada, Yogyakarta, Indonesia; ^2^Department of Histology and Cell Biology, Faculty of Medicine, Universitas Gadjah Mada, Yogyakarta, Indonesia

##### **Correspondence:** Dewi Kartikawati Paramita (dkparamita@ugm.ac.id) – Department of Histology and Cell Biology, Faculty of Medicine, Universitas Gadjah Mada, Yogyakarta, Indonesia

**Introduction** Nasopharyngeal carcinoma (NPC) is the second most common malignancy in Indonesia. Most of the cases are WHO III, of which almost 100 % are related to EBV infection. Most of NPC patients are diagnosed at the late stage (III-IV), with therapy failure as a consequence. Therefore, early diagnosis in high-risk population is needed. In order to develop a reliable test for early diagnosis, EBV-EA synthetic peptides were used as an antigen to detect IgA antibodies using the ELISA method. The use of synthetic peptides was proposed, due to its stability, it being easily produced and relatively more economical compared to natural or recombinant antigen.

**Aims** To evaluate the diagnostic values of IgA responses against two EA-p47/54 derived peptides (EA-1580 and EA-1582) by calculating the sensitivity, specificity, positive predictive value (PPV) and negative predictive value (NPV).

**Methods** Plasma of NPC cases were obtained from archives of NPC study at out institution (n = 100), while plasma from healthy control were collected at regional blood bank in Yogyakarta, Indonesia in 2014 (n = 100). Iga-EA peptides test were done using indirect ELISA. The difference response in both groups were analyzed using Mann-Whitney test. ROC analysis used to determine the cut-off value (CoV) in order to determine the sensitivity, specificity, PPV and NVP.

**Result and discussion** Mann-Whitney test showed that the IgA responses in both groups were significantly different (IgA-EA-1580 p value = 0.008; IgA EA-1582 p value = 0.026). The sensitivity, specificity, PPV and NPV of IgA –EA-1580 and IgA-EA-1582 were 44 %, 98 %, 95.6 %, 36.4 %; and 43 %, 97 %, 93.5 %, 63 %, respectively. These showed that capacity of both IgA-EA peptides were similar for NPC diagnostic tool.

## P18 Association between smoking habit and IgA-EBV titer among healthy individuals in Yogyakarta, Indonesia

### Benny Hermawan^1^, T Baning Rahayudjati^2^, Dewi K Paramita^3,4^, Jajah Fachiroh^3,4^

#### ^1^Study Program of Medicine, Faculty of Medicine, Universitas Gadjah Mada, Yogyakarta, Indonesia; ^2^Department of Public Health, Faculty of Medicine, Universitas Gadjah Mada, Yogyakarta, Indonesia; ^3^Department of Histology and Cell Biology, Faculty of Medicine, Universitas Gadjah Mada, Yogyakarta, Indonesia; ^4^Molecular Biology Laboratory, Faculty of Medicine, Universitas Gadjah Mada, Yogyakarta, Indonesia

##### **Corresponding:** Jajah Fachiroh (jajahfachiroh@ugm.ac.id) – Department of Histology and Cell Biology, Faculty of Medicine, Universitas Gadjah Mada, Yogyakarta

**Introduction** Elevated titer of IgA-EBV is a hallmark of NPC. However, previous studies in Yogyakarta showed that elevated IgA-EBV titer was observed among >20 % healthy individuals either among unaffected NPC families or non NPC familial individuals. Additionally, the elevated IgA-EBV titer might be transient due to immunity or other environmental factors, such as smoking habit.

**Aims.**To observe the association between smoking and IgA-EBV titer among healthy individuals.

**Methods** A cross sectional study was done in 2013, recruiting donors at local red cross unit. A self-filling questionnaire was used to obtain demographic and smoking habit information. The smoking information including smoking status, duration and numbers of cigarette smoked per day were collected. Parallel peripheral blood samples were collected and tested for IgA-[EBNA1 + VCA-p18]-ELISA. Association between smoking habit and IgA-EBV titer was analyzed using logistic regression, adjusted for age group and educational background.

**Result and discussion** Only males were included in this analysis (n = 240), with 47 subjects (19.60 %) had IgA-EBV titer above the ELISA cut-off value (0.35). Ever smoker was associated with high level of IgA-EBV (OR = 1.991; 95 % CI = 1.003-3.953). A dose response relationship was observed in smoking duration (OR = 1.237 for 1-10 years; OR = 1.954 for 11-20 years; OR = 3.637 for >20 years), smoking intensity (OR = 1.733 for 1-10 cigs/d; OR = 2.732 for >10 cigs/d) and cumulative number of cigarettes consumed (OR = 1.712 for 0.01-5.00 pack/year; OR = 1.994 for >5.00 pack/year).

**Conclusion** This study demonstrated the association between smoking habit and level of IgA-EBV titers in terms of duration, intensity, and cumulative number of cigarettes.

## P19 Epstein-Barr virus IgA titer comparison of healthy non-family individuals and healthy first degree family of NPV patients

### Gabriella Argy^1^, Jajah Fachiroh^2^, Dewi Kartikawati Paramita^2^, Susanna Hilda Hutajulu^3^

#### ^1^Study Program of Medicine, Faculty of Medicine, Universitas Gadjah Mada, Yogyakarta, Indonesia; ^2^Department of Histology and Cell Biology, Faculty of Medicine, Universitas Gadjah Mada, Yogyakarta, Indonesia; ^3^Division of Hematology and Medical Oncology, Department of Internal Medicine, Faculty of Medicine, Universitas Gadjah Mada/Dr Sardjito General Hospital, Yogyakarta, Indonesia

##### **Correspondence:** Susanna Hilda Hutajulu (susanna.hutajulu@ugm.ac.id) – Division of Hematology and Medical Oncology, Department of Internal Medicine, Faculty of Medicine, Universitas Gadjah Mada/Dr Sardjito General Hospital, Yogyakarta, Indonesia

**Introduction**: Epstein-Barr virus infection is associated with NPC in endemic populations. A screening method has been developed by detecting IgA-EBV among high risk groups such as healthy people with a high level of IgA-EBV titer or first degree of NPC subjects.

**Aims**: To compare the distribution of IgA-EBV titer in non-family healthy individuals and unaffected first degree family of NPC patients.

**Methods**: A cross sectional study among healthy blood donors (non family) was conducted in 2013. Demographic data and parallel peripheral blood samples were collected and tested by using IgA-[EBNA1 + VCA-p18]-ELISA. Distribution of IgA EBV titer was analyzed according to sex, age, education level and compared to unaffected first degree family of NPC patients (family; secondary data) using chi square with two-tailed p value ≤ 0.05 considered as significant. Cut off value (CoV) of IgA-[EBNA1 + VCA-p18] was determined at 0.35, and the value were divided into four groups of ≤ 0.35; 0.36-0.99; 1.00-1.99; and ≥ 2.00.

**Results and discussions.** We have analyzed 284 subjects from non family and 508 family members. When comparing demographic distribution of non family and family, male : female ratio were 7.2:1 and 1:1; age below 40y were 80 % and 46.7 %; education below university were 70 % and 91 %, respectively. Distribution of IgA-EBV titer were 79.9 %; 13.4 %; 6.7 %; and 0 % for non family and 58.8 %; 26.8 %; 12.6 %; 2.4 % for family (p < 0.001). The differences were determined by age group (p = 0.008) and education (p = 0.010).

## P20 Identification of EBV Early Antigen (EA) derived peptides for NPC diagnosis

### Theodora Caroline Sihotang^1^, Jajah Fachiroh^2,3^, Umi Intansari^4^, Dewi Kartikawati Paramita^2,3^

#### ^1^Study Program of Medicine, Faculty of Medicine, Universitas Gadjah Mada, Yogyakarta, Indonesia; ^2^Department of Histology and Cell Biology, Faculty of Medicine, Universitas Gadjah Mada, Yogyakarta, Indonesia; ^3^Molecular Biology Laboratory, Faculty of Medicine, Universitas Gadjah Mada, Yogyakarta, Indonesia; ^4^Department of Clinical Pathology, Faculty of Medicine, Universitas Gadjah Mada, Yogyakarta, Indonesia

##### **Correspondnece:** Dewi Kartikawati Paramita (dkparamita@ugm.ac.id) – Department of Histology and Cell Biology, Faculty of Medicine, Universitas Gadjah Mada, Yogyakarta, Indonesia

**Introduction** IgA response to EBV antigen has been proposed for nasopharyngeal cancer (NPC) serology based diagnosis and screening, especially Early Antigens (EA). Previous study showed that EA antigen had a high response in NPC patients, and no other EBV-related disease patients. Synthetic peptides have been proposed to replace natural antigens, as they are small molecules, easy to produce, and more stable. Synthetic peptides have to be able to replace EA epitope provided by the natural antigen.

**Aims** To identify six EA synthetic peptides for NPC diagnosis.

**Methods** Twenty (n = 20) NPC and healthy (n = 20) plasma samples were used to observe IgA response to EA-1580 and EA-1582 (derived from p47/54 protein sequence), EA-1579, EA-1101, EA-1102, and EA-1104 (derived from p138 protein sequence) peptides. Statistic analysis were performed using Mann-Whitney U test with P ≤ 0.05 regarded as statistically significance.

**Result and discussion** Mann-Whitney U test on IgA-EA-1579, IgA-EA-1580, IgA-EA-1582, IgA-EA-1101, IgA-EA-1102, and IgA-EA-1104 response showed P value of p < 0.0001 (U = 4), p < 0.0001 (U = 71), p = 0.001 (U = 83), p = 0.253 (U = 157), p = 0.529 (U = 176), and p = 0.002 (U = 9 0), respectively. This indicated that EA peptides derived from p138 protein sequence had the ability to differentiate healthy and NPC population better compared to EA derived from p47/54 protein. This merit further investigation to developed into diagnostic tool for NPC diagnosis.

## P21 Host-pathogen study: relative expression of mRNA BRLF1 Epstein-Barr virus as a potential biomarker for tumor progressivity and polymorphisms of *TCRBC* and *TCRGC2* host genes related to genetic susceptibility on nasopharyngeal carcinoma

### Daniel Joko Wahyono^1^, Purnomo Soeharso^2^, Dwi Anita Suryandari^2^, Lisnawati^3^, Zanil Musa^4^, Bambang Hermani^4^

#### ^1^Laboratory of Genetic, Faculty of Biology, Universitas Jenderal Soedirman, Purwokerto, Indonesia; ^2^Department of Biology Medicine, Faculty of Medicine, Universitas Indonesia, Jakarta, Indonesia; ^3^Department of Anatomic Pathology, Faculty of Medicine, Universitas Indonesia, Jakarta, Indonesia; ^4^Department of Otorhinolaryngology Head and Neck Surgery, Dr Cipto Mangunkusumo Hospital, Faculty of Medicine, Universitas Indonesia, Jakarta, Indonesia

##### **Correspondence:** Daniel Joko Wahyono (danieljokowahyono@yahoo.co.id; daniel.joko@unsoed.ac.id) – Laboratory of Genetic, Faculty of Biology, Universitas Jenderal Soedirman, Purwokerto, Indonesia

**Introduction** Nasopharyngeal carcinoma (NPC) is geographically endemic in the world, with multifactorial causes. EBV infection has been shown to consistently correlate with NPC, and is reflected by the expression of EBV latent and lytic genes. Particularly mRNA LMP1 could be used as a biomarker for EBV latent infection, while mRNA BRLF1 as biomarker for EBV lytic infection. As related to infection, polymorphisms of *TCRB* host gene could be associated with the susceptibility to NPC, particularly in the variable domain of TCR gene.

**Aims** The aims of this study were to determine firstly, the relative expression of mRNA BRLF1 EBV as a potential tumor progressivity biomarker in NPC, secondly, the polymorphisms of *TCRBC* and *TCRGC2* host gene related to the genetic susceptibility of NPC.

**Methods** This reseach designed as cross sectional study. The sample was represented by 82 NPC patients at Tumor Division, ENT Department, Dr. Cipto Mangunkusumo Hospital/Faculty of Medicine Universitas Indonesia, Jakarta. The expression of mRNA LMP1 EBV and that of mRNA BRLF1 EBV in NPC WHO-III patient were determined by RT-PCR technique. The relative expression of mRNA BRLF1 EBV was analysed by RT-qPCR technique and further calculated by 2^-ΔΔCt^ in NPC WHO-III patient who expressed mRNA LMP1 EBV and mRNA BRLF1 EBV.PCR-RFLP technique was done to determine *TCRBC* genotype (using *BglII* restriction site*)*, while *TCRGC2* genotype was identified by PCR-RFLP technique (using *PvuII* restriction site) and *nested* Long-PCR technique (exon II triplication *site existing*). The distribution of *TCRBC* and that of *TCRGC2* alotypes were calculated from the genotype proportion.

**Result and discussion** The positivity of mRNA expression of LMP1 and BRLF1 were 93,1 % and 65,2 %, respectively. The relative expression of BRLF1 EBV mRNA in NPC WHO-III patients in M_1_ status (distant metastases) increased 9.2 times compared to M_o_ status (without distant metastases) (n *=* 21; df = 1; p < 0,005). The relative expression of BRLF1 EBV mRNA in NPC WHO-III patients in stadium IV-C increased 10.2 times.

## P22 *In vitro* efficacy of silvestrol and episilvestrol, isolated from Borneo, on nasopharyngeal carcinoma, a major cancer in Borneo

### Maelinda Daker^1^, Yeo Jiun Tzen^2^, Norhasimah Bakar^1^, Asma’ Saiyidatina Aishah Abdul Rahman^2^, Munirah Ahmad^1^, Yeo Tiong Chia^2^, Alan Khoo Soo Beng^1^

#### ^1^Molecular Pathology Unit, Cancer Research Centre, Institute for Medical Research, 50588, Kuala Lumpur, Malaysia; ^2^Sarawak Biodiversity Centre, KM20, Semengoh, 93990 Kuching, Sarawak, Malaysia

##### **Correspondence:** Maelinda Daker (maelinda@imr.gov.my) - Molecular Pathology Unit, Cancer Research Centre, Institute for Medical Research, 50588, Kuala Lumpur, Malaysia

**Introduction** Current treatments for nasopharyngeal carcinoma (NPC) are often associated with potential toxicities. This has prompted efforts to find alternative sources. *Aglaia*, a genus of plants belonging to the family Meliaceae, can be found primarily in the forests in tropical Asia. Several species within the genus are known for the isolation of cyclopenta[b]benzofuran-flavaglines. Silvestrol and episilvestrol, members of this class of compounds, are isolated from the twig, fruit, and bark of *Aglaia stellatopilosa*, a species endemic to Borneo. In view of the exceptionally high incidence rate of NPC in the East Malaysian state of Sarawak particularly in the Bidayuh ethnic community, we decided to evaluate the role of these compounds, isolated from the natural resources of Borneo, for the treatment of a major cancer in Borneo itself.

**Aim** This work was carried out to study the inhibitory effects of silvestrol and episilvestrol on proliferation of human NPC cells.

**Methods** Silvestrol and episilvestrol were isolated from the bark of *Aglaia stellatopilosa*. Growth kinetics was monitored dynamically using a real-time, impedance-based cell analyzer. Dose-response profiles were generated using a colorimetric cell viability assay. Apoptosis was evaluated by flow cytometry and high content analysis. Flow cytometry was also performed to determine cell cycle distribution. Finally, the effects of combining silvestrol

or episilvestrol with cisplatin on NPC cells were studied.

**Results and discussion** Apoptosis was not observed in silvestrol- and episilvestrol-treated NPC cells but cell cycle perturbation was evident in NPC cells. Treatment with either compounds induced significant increase in the percentage of cells in G2/M phase compared to controls. Silvestrol or episilvestrol showed synergistic effects with cisplatin in inhibiting the proliferation of NPC cells. Our findings suggest that silvestrol and episilvestrol have anti-tumour activity in NPC cells. Further studies should be carried out on the mechanisms of action of these compounds in NPC and their potential as anti-NPC agents, particularly in combination with cisplatin.

## P23 The expression of mir-141 in patients with nasopharyngeal cancer

### Widyandani Sasikirana^1^, Tirta Wardana^1^, Muhammad Radifar^1^, Cita Herawati^2^, Agus Surono^3^, Sofia Mubarika Haryana^4^

#### ^1^Department of Biotechnology, Universitas Gadjah Mada, Yogyakarta, Indonesia; ^2^Dharmais National Cancer Hospital, Jakarta, Indonesia; ^3^Department of Otorhinolaryngology Head and Neck Surgery, Faculty of Medicine, Universitas Gadjah Mada, Yogyakarta, Indonesia; ^2^Department of Histology and Cell Biology, Faculty of Medicine, Universitas Gadjah Mada, Yogyakarta, Indonesia

##### **Correspondence:** Widyandani Sasikirana (widyandani.sasikirana@gmail.com) – Department of Biotechnology, Universitas Gadjah Mada, Yogyakarta, Indonesia

**Introduction** Nasopharyngeal carcinoma (NPC) is the fourth highest malignancy in Indonesia. MicroRNA is a potential biomarker for NPC early diagnosis. Previous studies indicated that miR-141 was oncomir, that target PTEN mRNA. Both acted on cisplatin resistance.

**Aims** To determine the expression profile of miR-141 and mRNA PTEN in blood plasma of nasopharyngeal carcinoma patients compared to healthy controls. Additionally, we wanted to determine the expression profile of miR-141 and PTEN mRNA in NPC patient prior and post theraphy.

**Methods** The study was conducted by in silico prediction to analyze the interaction of miR-141 with PTEN mRNA. Further laboratory research analyzed the expression level of miR-141 and PTEN mRNA compared to healthy controls. Expression analysis was performed using qRT-PCR with miR-16 and beta-actin as reference genes. The analysis of the relationship between miR-141 and PTEN mRNA was done through the chi square test.

**Results and discussion** Twenty-one samples (n = 21) out of 46 samples showed higher expression of miR-141. Expression of miR-141 in the blood plasma of nasopharyngeal carcinoma patients increased (up regulated) 1.49 times (p = 0.075, two-tailed t-test) compared to healthy controls. PTEN mRNA expression decreased (down-regulated) 0.65 times in blood plasma of nasopharyngeal carcinoma patients (p = 0.323, two-tailed t-test) compared to healthy controls. Correlation between miR-141 and PTEN mRNA was detected (p = 0.001). Expression of miR-141 in pre and post therapy patient was lower (0.61 times, p = 0.090) and expression of PTEN mRNA in pre and post therapy is down regulated (0.5, p = 0.090).Based on the results of the study, we concluded that there is a relationship between miR-141 with mRNA PTEN. The role of miR-141 as oncomir can suppress the expression of mRNA PTEN in post-transcriptional processes. Decreasing mRNA PTEN in patient pre and post therapy may be caused by cisplatin resistance.

